# Novel 2D CaCl crystals with metallicity, room-temperature ferromagnetism, heterojunction, piezoelectricity-like property and monovalent calcium ions

**DOI:** 10.1093/nsr/nwaa274

**Published:** 2020-11-07

**Authors:** Lei Zhang, Guosheng Shi, Bingquan Peng, Pengfei Gao, Liang Chen, Ni Zhong, Liuhua Mu, Lijuan Zhang, Peng Zhang, Lu Gou, Yimin Zhao, Shanshan Liang, Jie Jiang, Zejun Zhang, Hongtao Ren, Xiaoling Lei, Ruobing Yi, Yinwei Qiu, Yufeng Zhang, Xing Liu, Minghong Wu, Long Yan, Chungang Duan, Shengli Zhang, Haiping Fang

**Affiliations:** MOE Key Laboratory for Nonequilibrium Synthesis and Modulation of Condensed Matter, School of Physics, Xi’an Jiaotong University, Xi’an 710049, China; Shanghai Applied Radiation Institute, Shanghai University, Shanghai 200444, China; Department of Physics, East China University of Science and Technology, Shanghai 200237, China; MOE Key Laboratory for Nonequilibrium Synthesis and Modulation of Condensed Matter, School of Physics, Xi’an Jiaotong University, Xi’an 710049, China; Shanghai Institute of Applied Physics, Chinese Academy of Sciences, Shanghai 201800, China; MOE Key Laboratory for Nonequilibrium Synthesis and Modulation of Condensed Matter, School of Physics, Xi’an Jiaotong University, Xi’an 710049, China; Department of Optical Engineering, Zhejiang A&F University, Hangzhou 311300, China; Key Laboratory of Polar Materials and Devices of Ministry of Education, East China Normal University, Shanghai 200241, China; Shanghai Institute of Applied Physics, Chinese Academy of Sciences, Shanghai 201800, China; Shanghai Institute of Applied Physics, Chinese Academy of Sciences, Shanghai 201800, China; Zhangjiang Lab, Shanghai Advanced Research Institute, Chinese Academy of Sciences, Shanghai 201210, China; MOE Key Laboratory for Nonequilibrium Synthesis and Modulation of Condensed Matter, School of Physics, Xi’an Jiaotong University, Xi’an 710049, China; MOE Key Laboratory for Nonequilibrium Synthesis and Modulation of Condensed Matter, School of Physics, Xi’an Jiaotong University, Xi’an 710049, China; MOE Key Laboratory for Nonequilibrium Synthesis and Modulation of Condensed Matter, School of Physics, Xi’an Jiaotong University, Xi’an 710049, China; Shanghai Institute of Applied Physics, Chinese Academy of Sciences, Shanghai 201800, China; Shanghai Institute of Applied Physics, Chinese Academy of Sciences, Shanghai 201800, China; Shanghai Institute of Applied Physics, Chinese Academy of Sciences, Shanghai 201800, China; MOE Key Laboratory for Nonequilibrium Synthesis and Modulation of Condensed Matter, School of Physics, Xi’an Jiaotong University, Xi’an 710049, China; Department of Physics, East China University of Science and Technology, Shanghai 200237, China; Shanghai Institute of Applied Physics, Chinese Academy of Sciences, Shanghai 201800, China; Zhangjiang Lab, Shanghai Advanced Research Institute, Chinese Academy of Sciences, Shanghai 201210, China; MOE Key Laboratory for Nonequilibrium Synthesis and Modulation of Condensed Matter, School of Physics, Xi’an Jiaotong University, Xi’an 710049, China; Shanghai Applied Radiation Institute, Shanghai University, Shanghai 200444, China; Department of Optical Engineering, Zhejiang A&F University, Hangzhou 311300, China; College of Physical Science and Technology, Xiamen University, Xiamen 361005, China; Shanghai Applied Radiation Institute, Shanghai University, Shanghai 200444, China; Shanghai Applied Radiation Institute, Shanghai University, Shanghai 200444, China; Shanghai Institute of Applied Physics, Chinese Academy of Sciences, Shanghai 201800, China; Key Laboratory of Polar Materials and Devices of Ministry of Education, East China Normal University, Shanghai 200241, China; MOE Key Laboratory for Nonequilibrium Synthesis and Modulation of Condensed Matter, School of Physics, Xi’an Jiaotong University, Xi’an 710049, China; Department of Physics, East China University of Science and Technology, Shanghai 200237, China; Shanghai Institute of Applied Physics, Chinese Academy of Sciences, Shanghai 201800, China; Zhangjiang Lab, Shanghai Advanced Research Institute, Chinese Academy of Sciences, Shanghai 201210, China

**Keywords:** 2D CaCl crystals, monovalent calcium ions, room-temperature ferromagnetism, heterojunction, coexistence of piezoelectricity-like property and metallicity

## Abstract

Under ambient conditions, the only known valence state of calcium ions is +2, and the corresponding crystals with calcium ions are insulating and nonferromagnetic. Here, using cryo-electron microscopy, we report direct observation of two-dimensional (2D) CaCl crystals on reduced graphene oxide (rGO) membranes, in which the calcium ions are only monovalent (i.e. +1). Remarkably, metallic rather than insulating properties are displayed by those CaCl crystals. More interestingly, room-temperature ferromagnetism, graphene–CaCl heterojunction, coexistence of piezoelectricity-like property and metallicity, as well as the distinct hydrogen storage and release capability of the CaCl crystals in rGO membranes are experimentally demonstrated. We note that such CaCl crystals are obtained by simply incubating rGO membranes in salt solutions below the saturated concentration, under ambient conditions. Theoretical studies suggest that the formation of those abnormal crystals is attributed to the strong cation-π interactions of the Ca cations with the aromatic rings in the graphene surfaces. The findings highlight the realistic potential applications of such abnormal CaCl material with unusual electronic properties in designing novel transistors and magnetic devices, hydrogen storage, catalyzers, high-performance conducting electrodes and sensors, with a size down to atomic scale.

## Teaser

Counter to conventional wisdom, two-dimensional CaCl crystals with +1 calcium ions have been observed, which show unexpected metallicity, room-temperature ferromagnetism, heterojunction, piezoelectricity-like property, and distinct hydrogen storage and release capability.

## INTRODUCTION

Calcium ions are present in rocks, bones, shells, biominerals, geological deposits, ocean sediments and many other important materials [1]. Calcium ions in solution also play major roles in the retention of carbon dioxide in natural waters, water hardness, signal transduction and tissue generation [2–4]. As one of the alkaline earth metals, the calcium atom has two valence electrons according to the octet rule. Up to now, the only known valence state of calcium ions under ambient conditions was +2, and the corresponding crystals with calcium ions are insulating and nonferromagnetic [2]. If the valence of the calcium ion could be modified from divalence, which breaks with conventional theory, it could generate revolutionary new properties for the crystals with calcium ions, inducing a wide range of applications and novel insights.

Since the discovery of C_60_ [5], carbon nanotubes [6] and graphene [7], carbon has continued to surprise us. Up to now, most studies have focused on the morphologies and properties of those carbon-based structures and their complexes with other components having normal stoichiometry. Very recently, Na–Cl and K–Cl systems with abnormal stoichiometries on graphite/graphene under ambient conditions were reported [8]. The crystals are two-dimensional (2D) as they are formed in unsaturated solution on the graphene surfaces. Unfortunately, the atomic structures of these 2D Na–Cl crystals have not been experimentally observed and the corresponding properties have not been explored, partly because of the relatively weak cation-π interaction of the Na cations with the aromatic rings in the graphene surfaces resulting in relatively weak structural stability of the 2D Na–Cl crystals. We note that for divalent cations, the cation-π interactions are much stronger than the interactions for monovalent cations [9]. Here, using cryo-electron microscopy (cryo-EM), we directly observed 2D CaCl crystals on rGO membranes in unsaturated solution under ambient conditions. More importantly, the calcium ions in the CaCl crystals are monovalent (i.e. +1), and consequently the crystals display unexpected metallicity, room-temperature ferromagnetism, and resultant graphene–CaCl heterojunction, coexistence of piezoelectricity-like property and metallicity, together with distinct hydrogen storage and release capability under ambient conditions.

## RESULTS AND DISCUSSION

2D Ca–Cl crystals were obtained by soaking ultra-thin rGO membranes (thickness <10 nm) in CaCl_2_ solution below the saturated concentration under ambient conditions. This is quite similar to preparation of 2D Na–Cl crystals with Na_2_Cl and Na_3_Cl crystals in the rGO membranes in NaCl solution below saturated concentration [8], but the rGO membranes here were ultra-thin. The ultra-thin rGO membranes were fabricated by directly forming ultra-thin GO membranes on suitable supporting substrates, which were treated using glow-discharge and desorption methods, and then the ultra-thin GO membranes were reduced to rGO membranes under vacuum and high-temperature conditions (details in Supplementary data, section PS1). To prevent surface contamination from atmospheric air, the freshly prepared membranes were kept in vacuum and inert gas conditions. The supporting substrates were carbon holey films with hole sizes of ∼1.2 μm and center-to-center distances between neighboring holes of ∼2.5 μm. As the ultra-thin rGO membranes are quite flexible, the holey films support as well as provide substrate-free areas with optimized shapes and sizes for rGO membranes to be imaged by TEM. The ultra-thin membranes were then transferred to CaCl_2_ solution under protection of argon atmosphere conditions, and were incubated in 5.0 mol/L (M) CaCl_2_ solution overnight under ambient conditions. Preparation of Ca–Cl@rGO membranes of such thinness, corresponding to only several layers of the graphene sheets, allows clear characterization of the 2D Ca–Cl crystal structures and minimization of the unfavorable effects of inhomogeneous frozen rates on the membranes during the freezing process.

**Figure 1.**
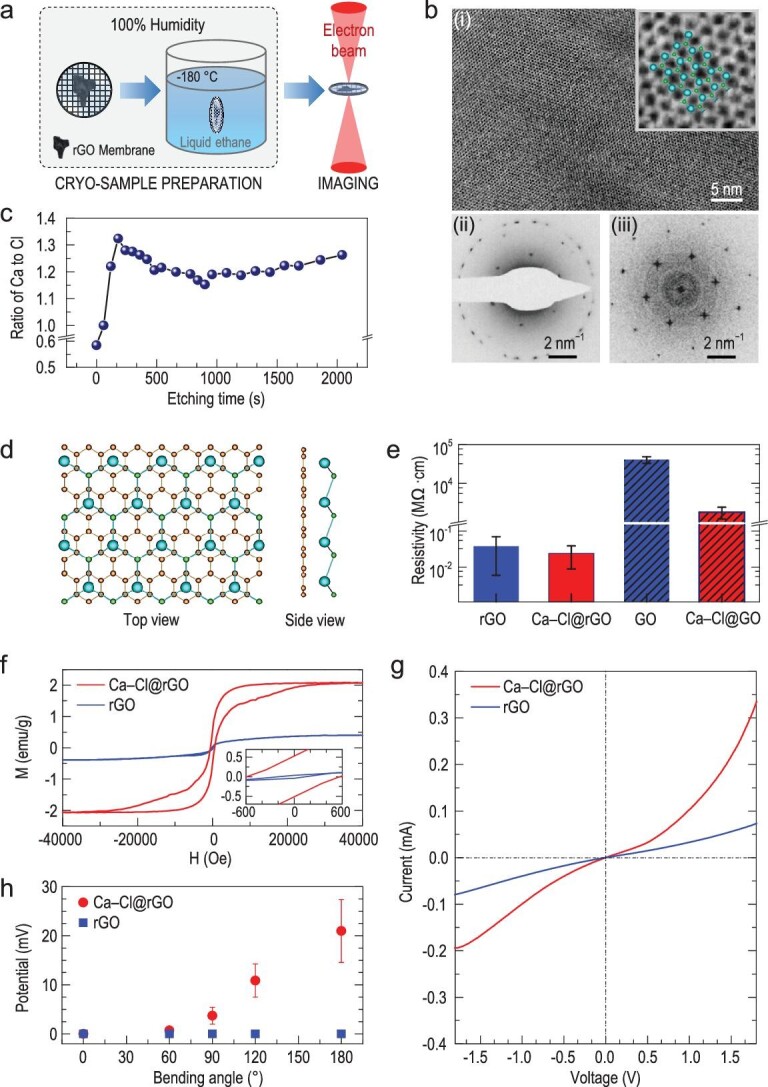
Two-dimensional (2D) Ca–Cl crystals from salt solution below the saturated concentration. (a) Schematic drawings of the sample preparation processes. (b) (i) Cryo-EM image of the Ca–Cl crystals in the ultra-thin reduced graphene oxide (rGO) membrane. Inset shows a zoomed-in area of the high-resolution image together with the CaCl crystal structure from the molecular model I, in which Ca and Cl atoms are shown as larger cyan spheres and smaller green spheres, respectively. (ii) Diffraction pattern of a typical crystal structure by cryo-EM in electron diffraction mode, showing a hexagonal lattice pattern with six first-order maxima points at (1 ± 0.03)/4.29 Å^−1^. (iii) Fourier transform of the entire bright-field image showing the same hexagonal lattice as in (ii). (c) Atomic ratio of Ca to Cl as a function of the etching time measured by XPS during etching by argon ion for a sample of the dried Ca–Cl@rGO membrane. (d) One stable structure from the molecular model I of CaCl crystal modules adsorbed on graphene sheet from theoretical computations. This structure exhibits P3m1 symmetry (space group: 156), with a lattice constant of 4.92 Å, a thickness of 6.65 Å from Cl to graphene, and a bond length of 3.01 Å from Cl to Ca. Ca, Cl and C atoms are in cyan, green and brown, respectively. (e) Electrical resistivity measured using the multimeter with two electrodes connecting with the up and down surfaces of the dried rGO and GO membranes, respectively. (f) Room-temperature ferromagnetism of the dried Ca–Cl@rGO membrane. Magnetization hysteresis loops of the dried rGO and the dried Ca–Cl@rGO membranes were measured at 300 K. The magnetic field is set to be perpendicular to the sample surface. (g) Heterojunction behavior of the dried Ca–Cl@rGO membrane. The current–voltage plot shows typical rectification behavior of the dried Ca–Cl@rGO membrane (red curve) compared with the dried rGO membrane (blue curve) under ambient conditions. (h) Piezoelectricity-like property of the dried Ca–Cl@rGO membrane under ambient conditions. Piezoelectric-like outputs from the dried Ca–Cl@rGO membrane (red dots) and the dried rGO membrane (blue squares) on the magnitude of the applied strains induced at different bending angles were measured.

The ultra-thin membranes coated with original salt solution were then flash-frozen at 20°C with 100% humidity and analyzed by cryo-EM [10,11] to study the *in situ* formation [12–14] of Ca–Cl crystals (Fig. 1a) in unsaturated solution. As the Ca–Cl crystals are 2D, and very sensitive to the high-energy electrons, the transmission electron microscopy (TEM) measurement conditions required to be optimized. We used 80-kV TEM high-tension together with low-dose mode, which has shown the best balance between the electron-beam damage increased at higher high-tensions and the resolution decreased at lower high-tensions. As a result, a stable single-crystal diffraction pattern was observed in the ultra-thin membranes (Figs 1b and S1). High-resolution cryo-EM images show that such crystals have a lattice spacing of 4.29 ± 0.14 Å (Fig. 1bi), corresponding to a graphene-like honeycomb lattice with a side length of 2.86 ± 0.09 Å. Electron diffraction and fast Fourier analyses [15] of the Ca–Cl lattice yielded a hexagonal lattice with first-order maximal points, that is (1−100) reflections, at (1 ± 0.03)/4.29 Å^−1^. Further, double-orientated crystals with the same lattice structure of the single Ca–Cl crystal were also observed both on TEM diffraction patterns and high-resolution images (Fig. S2). In Figs 1bii and S1, we can see that there are about five sets of hexagonal diffraction spots at ∼1/2.13 Å^−1^, too, which correspond to the (1−100) reflections of the graphene. Notably, the (2−200) reflections of the Ca–Cl crystals are coincident with these (1−100) reflections of graphene (Figs 1bii and S1).

Then, we measured the atomic ratio of Ca to Cl and the properties of this Ca–Cl crystal. The ultra-thin Ca–Cl@rGO membranes, which include only several layers of graphene sheets, are too thin to perform elemental analysis and it is too difficult to perform measurements of the electrical, magnetic and hydrogen storage properties. We fabricated thick freestanding rGO membranes (thickness >10 μm) [8,16,17] from an rGO suspension (see Supplementary data, section PS1). The membranes were incubated in 5.0 M CaCl_2_ solutions overnight and then centrifuged to remove free solution and dried at 70°C for 4 hours. We called the resultant membranes the ‘dried Ca–Cl@rGO membranes.’ Double- and multiple-orientated Ca–Cl crystals were observed on conventional TEM images of these dried Ca–Cl@rGO membranes (Fig. S4). The hexagonal (2−200) reflections of the Ca–Cl crystals are also consistent with the (1−100) reflections of the graphene sheets, demonstrating that the structure of the Ca–Cl crystals in the dried Ca–Cl@rGO membranes is consistent with the structure of the Ca–Cl crystals observed above in the ultra-thin Ca–Cl@rGO membranes.

Atomic ratios of Ca to Cl in the dried Ca–Cl@rGO membranes were measured by conventional (non-cryogenic) TEM energy-dispersive X-ray spectroscopy (EDS) analysis and X-ray photoelectron spectroscopy (XPS) (see Supplementary data, section PS1). The dark-field high-angle annular dark field scanning TEM (HADDF-STEM) image shows typical distributions of the Ca–Cl crystals in the membranes (Fig. S7). Interestingly, the corresponding TEM EDS analysis (Fig. S7) shows that, in addition to the formation of regular CaCl_2_ crystals, there are some stable structures with a Ca : Cl ratio of ∼1 : 1, which we denoted as CaCl. To further investigate the stoichiometries of Ca to Cl in the membranes, we performed XPS elemental analysis by etching the membrane with argon ions. The depth profile shows that the Ca : Cl ratios vary from a value below 0.6 : 1 at the membrane top surface to a stable ∼1.2 : 1 in the inner sheets (Fig. 1c). The membrane surface may exhibit regular CaCl_2_ because of evaporation of adsorbing salt solution, but on the inner sheets there are mainly CaCl crystals with a stable Ca : Cl ratio of ∼1 : 1.

To discuss the possible structures of the crystals, we used density-functional theory (DFT) [18,19] combined with CALYPSO and USPEX methods [20,21] (see Supplementary data, sections PS1 and PS3) to obtain a stable structure with a Ca : Cl ratio of 1 : 1, which could be denoted as CaCl crystal (Figs 1d and S11a, model I). Interestingly, as shown in Fig. 1bi, the atomic-resolution TEM images of the CaCl crystal consistent with this structure were experimentally obtained. In this structure, the Ca occupies a plane parallel to the graphene sheet and is located in the center of nonadjacent aromatic rings. Difference charge density shows that there is significant charge transfer between Ca and Cl (Fig. S15). The charge is almost all around the Cl, which shows strong ionic bond characteristics, and the charges of graphene and Ca are assembled to the space between Ca and graphene, demonstrating significant cation-π interaction between Ca and the aromatic rings in graphene. Numerical Bader charge analysis [22] shows that Ca transfers ∼0.85 (close to 1) electron to Cl and shares ∼0.65 electron with graphene (Table S3). However, the charge shared with graphene is not induced by normal electrovalent bond but the electron delocalization via cation-π interaction, resulting in calcium acting as an ion of +1 valence state (see Supplementary data, section PS4). Chemical bonding analyses of Ca in the Ca–Cl@rGO membranes show that there is only one ionic bonding between the Ca cation and the Cl anion, that is the Ca cation is monovalent, and there is no chemical bonding between the Ca cation and the graphene as the cation-π interaction has been named as the non-covalent interaction [23,24] (see Supplementary data, section PS4). Further, there are other possible structures of Ca–Cl, including another CaCl (model II and Fig. S11a) and regular CaCl_2_, Ca_3_Cl_4_, Ca_3_Cl_2_ (Figs S11b and S11c), on the graphene surface or confined between graphene sheets.

**Figure 2.**
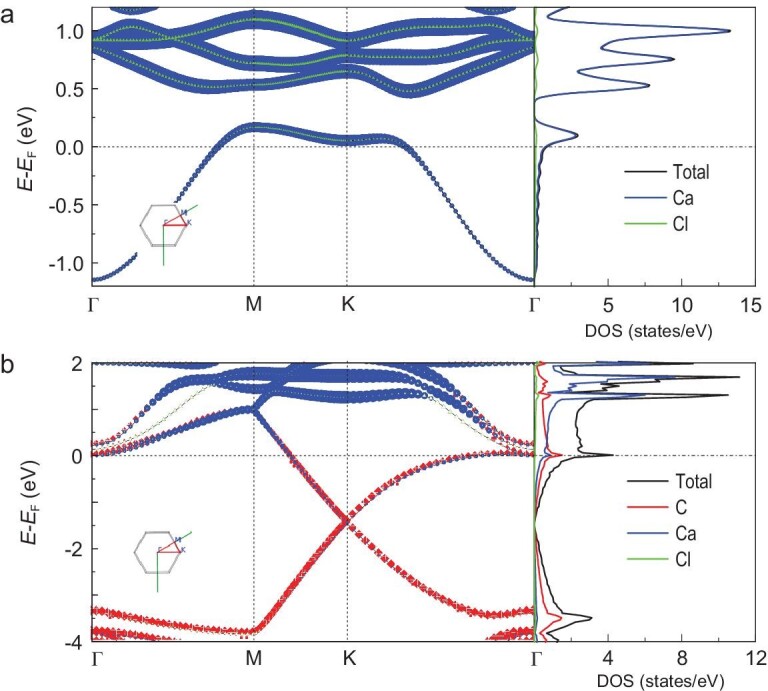
Electronic structure of model I. (a) Projected band structure and projected DOS on each element in the primitive cell of CaCl alone. (b) Projected band structure and projected DOS on each element in the primitive cell of full model. The size of the points in (a) and (b) indicates the weight of each element's contribution. Inset: the high symmetry q point path in the Brillouin Zone. *E_F_*, Fermi energy.

These CaCl crystals with unusual electronic structure of +1 calcium ions exhibit revolutionary new properties for the crystals with calcium ions, such as metallicity, ferromagnetism, heterojunction, coexistence of piezoelectricity-like property and metallicity, and adsorption capacity with hydrogen storage. The projected electronic band structure and density of states (DOS) of model I shows that CaCl crystals have distinct metallicity in which Ca plays a dominant role (Fig. 2a). The complexes of graphene with CaCl crystals also have strong metallicity, and the Ca and Cl together contribute ∼37.7% of the total electronic states near the Fermi level, indicating that CaCl crystals are also metallic in this system (Fig. 2b). More remarkably, compared with pristine 2D graphene and 2D CaCl crystal, the complex shows greatly enhanced electrical conductivity along the horizontal direction, as well as the vertical direction in the case of membrane (Figs S16–18 and Table S5).

**Figure 3.**
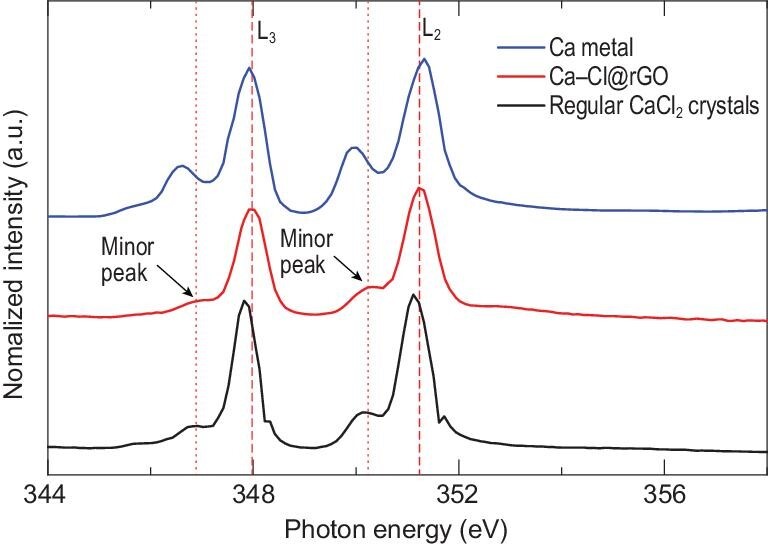
Calcium near-edge X-ray absorption fine structure spectra (NEXAFS) of the regular calcium metal, the regular CaCl_2_ crystal and the CaCl crystals in rGO membranes.

Abnormal valence state change of the CaCl crystals was analyzed and confirmed by soft X-ray absorption spectroscopy (near-edge X-ray absorption fine structure spectra, NEXAFS). In addition to the L_2,3_-edge peaks for regular CaCl_2_ at 347.82 and 351.12 eV, there are new Ca L_2,3_-edge peaks at 348.02 and 351.22 eV (Figs 3, S21 and S22). These new L_2,3_-edge peaks are clearly different from the calcium metal with Ca–Ca bonding and the regular CaCl_2_ crystals with Ca^2+^ (Fig. 3). Importantly, the photo energy of the new L_3_-edge peak of Ca from the dried Ca–Cl@rGO (∼348.02 eV) membrane is larger than both that of pure calcium metal (∼347.92 eV) and of regular CaCl_2_ crystals (∼347.82 eV) (see further discussion in Supplementary data, section PS4). To further verify this phenomenon of valence state change, we performed NEXAFS experiments on the dried Cu–Cl@rGO membrane, which was prepared by incubating the rGO membranes with CuCl_2_ solutions. The L_2,3_-edge peaks for Cu in the dried Cu–Cl@rGO membrane are consistent with the L_2,3_-edge peaks in the regular CuCl crystal, demonstrating that the valence state of Cu has been changed from +2 to +1 in the dried Cu–Cl@rGO membrane (see further discussion in Supplementary data, section PS5).

Metallic properties of CaCl crystals were confirmed experimentally by measuring the electrical resistivity (see Supplementary data, sections PS1 and PS6). Figure 1e shows that the resistivities of pure dried rGO membranes and Ca–Cl@rGO membranes are both less than ∼4.0 × 10^−2^ MΩ·cm, indicating their good conductivity. We note that our result on rGO membranes is consistent with metallic properties reported in the literature [25]. In contrast, for dried GO membranes with high resistivity, the average resistivity of dried Ca–Cl@GO membranes was reduced by at least one order of magnitude (Fig. 1e). The metallicity of CaCl crystals was further demonstrated using conductive atomic force microscopy (AFM) on the graphene sheets (see Supplementary data, sections PS1 and PS6).

Room-temperature ferromagnetism of the CaCl crystals in rGO membrane was also experimentally observed. Figure 1f shows that the saturation magnetic moment (*M_s_*) of the dried CaCl@rGO membrane is ∼2.1 emu/g at 300 K, whereas *M_s_* = ∼0.4 emu/g for the rGO membrane at the same temperature, showing a ∼400% enhancement of magnetism by introducing only ∼5% (wt, Fig. S8) calcium to the membrane. For a system with periodic model I configuration, Stoner instability analysis and polarized DFT calculations [26] do not suggest magnetism. Further DFT calculations revealed that the possible origin of such strong ferromagnetism is the edge or defect effects of the CaCl crystals where there is an unpaired valence electron in Ca^+^, and the saturation magnetic moment computed is consistent with the experimental observations (see Supplementary data, section PS9).

A graphene–CaCl heterojunction was experimentally demonstrated. The current–voltage curve of the dried Ca–Cl@rGO junction membrane under positive and negative gate voltages is obviously asymmetric, indicating typical rectification behavior (Fig. 1g). Further, the +1 valence in these CaCl crystals provides distinct adsorption capacity with hydrogen storage, which not only can fill the insufficient adsorption of metallic calcium atoms, but also overcome the exorbitant absorption of +2 calcium ions (see Supplementary data, section PS10).

The metallic CaCl crystals in rGO membrane possess unexpected piezoelectricity-like property. DFT calculations show that, for 5% geometric elongation along the x direction, the charge distribution of the Ca, Cl and C atoms in model I has significant relocation (Fig. S29). Our experiment, where a dried Ca–Cl@rGO membrane was connected by two Cu foil electrodes (Figs 1h and S33a), shows a maximal output voltage of ∼4 mV when the membrane is bent with an angle of 90° (Fig. S33c) to generate large enough strains in the membrane, and the voltage decreases, reaches zero and may even overshoot to negative values when we remove the bending force. In contrast, we cannot detect a visible output voltage for the rGO membrane with the same bending angles. The voltage of the Ca–Cl@rGO membrane increases with increasing bending degree (Fig. 1h). Although our theoretical calculations strongly support the origin of such phenomenon being the piezoelectricity of the Ca–Cl@rGO membrane, we cannot exclude the possibility that the visible voltage outputs may also be partly induced by other effects during sample bending. Therefore, we refer to this phenomenon as piezoelectricity-like property based on current experimental observations.

## CONCLUSION

Nature continuously produces surprises, and our results demonstrate another one. 2D Na–Cl crystals with unconventional stoichiometries on graphite or graphene [8] and at extreme conditions, such as high pressure [27] have been reported. Here we observed 2D CaCl crystals with abnormal stoichiometries directly on rGO membranes in unsaturated solution, and remarkably, demonstrate experimentally the +1 valence of Ca ions together with various unexpected properties and applications of the crystals, including metallicity, room-temperature ferromagnetism, and resultant graphene–CaCl heterojunction, coexistence of piezoelectricity-like property and metallicity, as well as distinct hydrogen storage and release capability.

Such abnormal CaCl crystals are attributed to the strong cation-π interactions between Ca cations and aromatic rings in the graphene surfaces. As strong cation-π interactions also exist between other metal cations (such as Mg^2+^, Fe^2+^, Co^2+^, Cu^2+^, Cd^2+^, Cr^2+^ and Pb^2+^) and graphitic surfaces [8,9,23,24,28–32], similar 2D crystals with abnormal valence state of the metal cations on graphitic surface and corresponding abnormal properties as well as novel applications are highly expected. In fact under ambient conditions, CuCl crystals with +1 copper ions can exist stably for several days (see Supplementary data, section PS5) and have room-temperature ferromagnetism (see Supplementary data, section PS9). This would help to overcome the difficulty of the short lifetime in application as a catalyzer for the +1 copper ion [33]. We note that it is expected that every metal element has room-temperature ferromagnetism via formation of the corresponding abnormal 2D crystals with an unpaired valence electron in the metal ions. Further, considering the wide distribution of metallic cations and carbon on Earth, such nanoscale ‘special’ compounds with previously unrecognized properties may be ubiquitous in nature.

## METHODS

See Supplementary section PS1 for details.

## Supplementary Material

nwaa274_Supplemental_File

## References

[1] Gebauer D , Volkel A, Colfen H. Stable prenucleation calcium carbonate clusters. Science 2008; 322: 1819–22. 10.1126/science.1164271 19095936

[2] Greenwood NN , Earnshaw A. Chemistry of the Elements (Second Edition). Boston: Butterworth-Heinemann, 1997.

[3] Sigel A , Sigel H, Sigel RKO. Interrelations Between Essential Metal Ions and Human Diseases. New York: Springer, 2013.

[4] Berridge MJ , Bootman MD, Lipp P. Calcium—a life and death signal. Nature 1998; 395: 645–8. 10.1038/27094 9790183

[5] Kroto HW , Allaf AW, Balm SP. C60—Buckminsterfullerene. Chem Rev 1991; 91: 1213–35. 10.1021/cr00006a005

[6] Iijima S . Helical microtubules of graphitic carbon. Nature 1991; 354: 56–8. 10.1038/354056a0

[7] Novoselov KS , Geim AK, Morozov SV et al. Electric field effect in atomically thin carbon films. Science 2004; 306: 666–9. 10.1126/science.1102896 15499015

[8] Shi G , Chen L, Yang Y et al. Two-dimensional Na-Cl crystals of unconventional stoichiometries on graphene surface from dilute solution at ambient conditions. Nat Chem 2018; 10: 776–9. 10.1038/s41557-018-0061-4 29736004

[9] Shi G , Liu J, Wang C et al. Ion enrichment on the hydrophobic carbon-based surface in aqueous salt solutions due to cation-π interactions. Sci Rep 2013; 3: 3436. 10.1038/srep03436 24310448 PMC3853681

[10] Rao Q , Liu M, Tian Y et al. Cryo-EM structure of human ATR-ATRIP complex. Cell Res 2018; 28: 143–56. 10.1038/cr.2017.158 29271416 PMC5799817

[11] Zhang L , Yan F, Zhang SL et al. Structural basis of transfer between lipoproteins by cholesteryl ester transfer protein. Nat Chem Biol 2012; 8: 342–9. 10.1038/nchembio.796 22344176 PMC3792710

[12] De Yoreo JJ , Sommerdijk N. Investigating materials formation with liquid-phase and cryogenic TEM. Nat Rev Mater 2016; 1: 16035. 10.1038/natrevmats.2016.35

[13] Li Y , Li Y, Pei A et al. Atomic structure of sensitive battery materials and interfaces revealed by cryo-electron microscopy. Science 2017; 358: 506–10. 10.1126/science.aam6014 29074771

[14] Pantelic RS , Suk JW, Magnuson CW et al. Graphene: Substrate preparation and introduction. J Struct Biol 2011; 174: 234–8. 10.1016/j.jsb.2010.10.002 20937392

[15] Yuk JM , Park J, Ercius P et al. High-resolution EM of colloidal nanocrystal growth using graphene liquid cells. Science 2012; 336: 61–4. 10.1126/science.1217654 22491849

[16] Eda G , Fanchini G, Chhowalla M. Large-area ultrathin films of reduced graphene oxide as a transparent and flexible electronic material. Nat Nanotechnol 2008; 3: 270–4. 10.1038/nnano.2008.83 18654522

[17] Kim HW , Yoon HW, Yoon SM et al. Selective gas transport through few-layered graphene and graphene oxide membranes. Science 2013; 342: 91–5. 10.1126/science.1236098 24092738

[18] Serr A , Netz RR. Polarizabilities of hydrated and free ions derived from DFT calculations. Int J Quantum Chem 2006; 106: 2960–74. 10.1002/qua.21121

[19] Xu LC , Wang RZ, Miao MS et al. Two dimensional Dirac carbon allotropes from graphene. Nanoscale 2014; 6: 1113–8. 10.1039/C3NR04463G 24296630

[20] Oganov AR , Glass CW. Crystal structure prediction using ab initio evolutionary techniques: principles and applications. J Chem Phys 2006; 124: 244704. 10.1063/1.2210932 16821993

[21] Wang YC , Lv JA, Zhu L et al. Crystal structure prediction via particle-swarm optimization. Phys Rev B 2010; 82: 094116. 10.1103/PhysRevB.82.094116

[22] Bader RFW . A quantum-theory of molecular-structure and its applications. Chem Rev 1991; 91: 893–928. 10.1021/cr00005a013

[23] Ma JC , Dougherty DA. The cation-π interaction. Chem Rev 1997; 97: 1303–24. 10.1021/cr9603744 11851453

[24] Mahadevi AS , Sastry GN. Cation-π interaction: its role and relevance in chemistry, biology, and material science. Chem Rev 2013; 113: 2100–38. 10.1021/cr300222d 23145968

[25] Pei SF , Cheng HM. The reduction of graphene oxide. Carbon 2012; 50: 3210–28. 10.1016/j.carbon.2011.11.010

[26] Perdew JP , Zunger A. Self-interaction correction to density-functional approximations for many-electron systems. Phys Rev B 1981; 23: 5048–79. 10.1103/PhysRevB.23.5048

[27] Zhang WW , Oganov AR, Goncharov AF et al. Unexpected stable stoichiometries of sodium chlorides. Science 2013; 342: 1502–5. 10.1126/science.1244989 24357316

[28] Chen L , Shi GS, Shen J et al. Ion sieving in graphene oxide membranes via cationic control of interlayer spacing. Nature 2017; 550: 415–8. 10.1038/nature24035 28992630

[29] Gebbie MA , Wei W, Schrader AM et al. Tuning underwater adhesion with cation-π interactions. Nat Chem 2017; 9: 473–9. 10.1038/nchem.2720 28430190

[30] Liu J , Shi G, Guo P et al. Blockage of water flow in carbon nanotubes by ions due to interactions between cations and aromatic rings. Phys Rev Lett 2015; 115: 164502. 10.1103/PhysRevLett.115.164502 26550880

[31] Shi G , Dang Y, Pan T et al. Unexpectedly enhanced solubility of aromatic amino acids and peptides in an aqueous solution of divalent transition-metal cations. Phys Rev Lett 2016; 117: 238102. 10.1103/PhysRevLett.117.238102 27982649

[32] Xiu X , Puskar NL, Shanata JA et al. Nicotine binding to brain receptors requires a strong cation-π interaction. Nature 2009; 458: 534–7. 10.1038/nature07768 19252481 PMC2755585

[33] Gawande MB , Goswami A, Felpin FX et al. Cu and Cu-based nanoparticles: synthesis and applications in review catalysis. Chem Rev 2016; 116: 3722–811. 10.1021/acs.chemrev.5b00482 26935812

