# Supplementary material for: Novel 2D CaCl crystals with metallicity, room-temperature ferromagnetism, heterojunction, piezoelectricity-like property and monovalent calcium ions
Source: Natl Sci Rev. 2020 Nov 7;8(7):nwaa274. doi: 10.1093/nsr/nwaa274 (PMC8310769; doi:10.1093/nsr/nwaa274)
Supplement: nwaa274_Supplemental_File [file nwaa274_supplemental_file.docx]

**SUPPLEMENTARY DATA FOR**

**Novel 2D CaCl crystals with metallicity, room-temperature ferromagnetism, heterojunction, piezoelectricity-like property, and monovalent calcium ions**

**Authors:** Lei Zhang^1^†, Guosheng Shi^2,3^†, Bingquan Peng^1,4^†, Pengfei Gao^1^†, Liang Chen^5^, Ni Zhong^6^, Liuhua Mu^4^, Lijuan Zhang^4,7^, Peng Zhang^1^, Lu Gou^1^, Yimin Zhao^1^, Shanshan Liang^4^, Jie Jiang^4^, Zejun Zhang^4^, Hongtao Ren^1^, Xiaoling Lei^3,4,7^, Ruobing Yi^1,2^, Yinwei Qiu^5^, Yufeng Zhang^8^, Xing Liu^2^, Minghong Wu^2^, Long Yan^4*^, Chungang Duan^6*^, Shengli Zhang^1*^ & Haiping Fang^3,4,7*^

**Affiliations:**

^1^MOE Key Laboratory for Nonequilibrium Synthesis and Modulation of Condensed Matter, School of Physics, Xi'an Jiaotong University, Xi'an 710049, China

^2^Shanghai Applied Radiation Institute, Shanghai University, Shanghai 200444, China

^3^Department of Physics, East China University of Science and Technology, Shanghai 200237, China

^4^Shanghai Institute of Applied Physics, Chinese Academy of Sciences, Shanghai 201800, China

^5^Department of Optical Engineering, Zhejiang A&F University, Hangzhou 311300, China

^6^Key Laboratory of Polar Materials and Devices, Ministry of Education, East China Normal University, Shanghai 200241, China

^7^Zhangjiang Lab, Shanghai Advanced Research Institute, Chinese Academy of Sciences, Shanghai 201210, China

^8^College of Physical Science and Technology, Xiamen University, Xiamen 361005, China

*Correspondence to: fanghaiping@sinap.ac.cn (H.F.); zhangsl@xjtu.edu.cn (S.Z.); cgduan@clpm.ecnu.edu.cn (C.D.); yanlong@sinap.ac.cn (L.Y.)

†These authors contributed equally to this work.

**This PDF file includes:**

Materials and Methods

Supplementary Text

Figs. S1 to S43

Tables S1 to S8

**Table of contents**

[**PS1: Materials and methods** 3](#_Toc54717318)

[**Experimental materials and methods:** 3](#_Toc54717319)

[**Fabrication of the ultra-thin rGO membranes** 3](#_Toc54717320)

[**Preparation of the frozen wet ultra-thin Ca–Cl@rGO membranes** 3](#_Toc54717321)

[**Fabrication of thick freestanding GO and rGO membranes** 3](#_Toc54717322)

[**TEM data collection** 4](#_Toc54717323)

[**XPS data collection** 4](#_Toc54717324)

[**ICP data collection** 4](#_Toc54717325)

[**NEXAFS data collection** 4](#_Toc54717326)

[**Conductive AFM data collection** 5](#_Toc54717327)

[**Electrical resistivity measurement** 5](#_Toc54717328)

[**Heterojunction behavior measurement** 5](#_Toc54717329)

[**Piezoelectricity-like property measurement** 5](#_Toc54717330)

[**Room-temperature ferromagnetism measurement** 5](#_Toc54717331)

[**Hydrogen storage capacity measurement** 6](#_Toc54717332)

[**Theoretical computation methods:** 6](#_Toc54717333)

[**Crystal structure prediction, optimization and electronic property calculations** 6](#_Toc54717334)

[**Thermodynamic stability analysis of the Ca–Cl crystal structures** 7](#_Toc54717335)

[**Bader charge analysis** 7](#_Toc54717336)

[**Electrical conductivity calculation** 7](#_Toc54717337)

[**Piezoelectricity-like property calculation** 8](#_Toc54717338)

[**Ferromagnetism and antiferromagenetism calculation** 8](#_Toc54717339)

[**Hydrogen storage capacity calculation** 8](#_Toc54717340)

[**PS2: Structure determination of Ca–Cl crystals by cryo-EM, conventional TEM and XPS** 8](#_Toc54717341)

[**PS3: Structure modelling of the Ca–Cl crystals by DFT and USPEX** 16](#_Toc54717342)

[**PS4: Valence analysis of the Ca–Cl crystals** 28](#_Toc54717343)

[**PS5: Valence analysis of the Cu–Cl crystals** 32](#_Toc54717344)

[**PS6: Electrical resistivity measurement and calculation** 33](#_Toc54717345)

[**PS7: Graphene–CaCl heterojunction behavior measurement** 36](#_Toc54717346)

[**PS8: Piezoelectricity-like property calculation and measurement** 37](#_Toc54717347)

[**PS9: Room-temperature ferromagnetism measurement and calculation** 42](#_Toc54717348)

[**PS10: Hydrogen storage capacity calculation and measurement** 48](#_Toc54717349)

**PS1: Materials and methods**

**Experimental materials and methods:**

**Fabrication of the ultra-thin rGO membranes**

The ultra-thin rGO (thickness < 10 nm, size > 2×10^3^ μm) membranes were fabricated by dropping 2.5 μL of dialyzed GO suspension with a concentration of 0.04 mg/mL on a holey carbon-film coated TEM grid, which has circular and orthogonal arranged holes with hole sizes of ~1.2 μm and center-to-center distances of ~2.5 μm. This grid was heated at 453 K for 3 h under vacuum to convert the GO into rGO membranes on the grid.

**Preparation of the frozen wet ultra-thin Ca–Cl@rGO membranes**

The **frozen wet** ultra-thin Ca–Cl@rGO membranes with large-area CaCl crystals were prepared by incubating the ultra-thin rGO membranes in 5.0 M CaCl_2_ solution and then plunge freezing. In brief, once the ultra-thin rGO membranes were obtained from the ultra-thin GO membranes under vacuum condition at 453 K, the temperature was decreased to room temperature with a step of 0.2 K/s while the vacuum condition was remained. As soon as the temperature reached 300 K, the membranes were transferred into CaCl_2_ solution within 1 minute with the protection of argon atmosphere condition. The membranes were then immersed in 5.0 M CaCl_2_ solution for incubation under ambient conditions. After incubated with CaCl_2_ solution for overnight, the **frozen wet** samples were prepared by using a FEI Vitrobot rapid-plunging device with conditions set at 100% humidity and room temperature, and the sample was blotted with filter paper (#595, Schleicher & Schuell) for 2 s and flash-frozen in liquid ethane. The frozen-hydrated specimen was transferred to liquid nitrogen and placed onto a Gatan 626 cryo-TEM holder while immersed in liquid nitrogen and isolated from the environment by a tightly closed shutter. During insertion into the TEM column, the temperature of the sample was kept below −170 °C.

**Fabrication of thick freestanding GO and rGO membranes**

The freestanding the pure dried GO and rGO membranes were prepared from natural graphite powders via a modified Hummer method as described previously [8,16,17]. The prepared GO and rGO membranes were stored in dry and clean containers before usage.

**The dried Ca–Cl@rGO and Ca–Cl@GO membranes for** **conventional (non-cryogenic) TEM, XPS, NEXAFS, electrical resistivity and hydrogen storage measurement** were prepared by first incubating the pure dried rGO or GO membranes in 5.0 M CaCl_2_ solutions overnight under ambient conditions and then centrifuging to remove the free solution and drying at 70 °C for more than 4 hours. For conventional TEM imaging, the dried Ca–Cl@rGO membranes were manually split into small pieces, and one piece of the membrane was transferred and deposited directly onto a film-free Cu TEM grid.

**The dried Ca–Cl@graphene sheets for conductive AFM** were prepared by transferring graphene sheets on Au coated quartz substrates. In brief, graphene sheets were exfoliated on Au coated quartz substrates from the highly oriented pyrolytic graphites (GRAPHENE SUPERMARKET, Grade 50) by micromechanical cleavage [7]. Some Au coated quartz substrates with graphene sheets were soaked in 5.0 M CaCl_2_ solution for 12 hours under ambient conditions. The specimens were taken out and rinsed with DI water for 5–10 seconds to remove the free solution of CaCl_2_, and then dried at 70 °C for 6 hours.

**TEM data collection**

**Cryo-EM micrographs** were acquired at −180 °C on a FEI CETA 4k × 4k CMOS camera by a FEI F200C transmission electron microscope (TEM) operating at 200-kV or 80-kV and low-electron-dose conditions. Selected-area electron diffraction (SAED) images were taken with a ~350 nm diameter selected-area (SA) aperture. The exposure time was varied between 0.5 and 2 s, and binning 1 was adopted for 80-kV high-tension, binning 2 was adopted for 200-kV high-tension, for acquiring the images.

**Conventional (non-cryogenic) high-resolution TEM micrographs and SAED images** were acquired at room temperature by the same FEI F200C TEM operating at 200-kV. High-angle annular dark field scanning TEM (HADDF-STEM) and energy-dispersive X-ray spectroscopy (EDS) were performed at room temperature on a JEOL 4k × 4k CMOS camera by a JEOL JEM-F200 (HR) TEM operating at 200-kV.

**XPS data collection**

The dried Ca–Cl@rGO membranes were characterized by X-ray photoelectron spectroscopy (XPS), which was performed on a Thermo Fisher ESCALAB Xi^+^ system. The atomic percentage of each element in different depth of dried membranes was obtained by XPS analysis with argon ion etching. The instrument conditions were as follows: source gun type, Al K Alpha; ion energy, 3000 eV; current, 2.73 μA; raster size, 2.5 mm; spot size, 650 μm.

**ICP data collection**

The dried Ca–Cl@rGO and pure dried rGO membranes were incubated with 9.0 mL concentrated HNO_3_ (70%) and 1.0 mL HClO_4_ catalyst in a 300 mm long Pyrex glass digestion tube for digesting overnight, during when the temperature was increased gradually starting from 100 °C to 180 °C in a heating block. Then the solution was left in room temperature to be cooled down and transferred to a volumetric flask to be diluted with DI water to a volume of 50 mL. Finally the diluted solution was filtered and analyzed by using a PS7800 inductively coupled plasma optical emission spectrometer (ICP-OES).

**NEXAFS data collection**

NEXAFS spectra of Ca and Cu compounds near the L-edge were collected on the beam line 08U1A of Shanghai Synchrotron Radiation Facility (SSRF Shanghai, China). The electron energy in the storage ring was about 3.5 GeV with a current of about 230 mA. Spectra were recorded at the selected energies through the Ca L-edge (340–360 eV) and the Cu L-edge (910–950 eV) with an energy step of 0.1 eV (for Ca) and 0.2 eV (for Cu). The 99.5% pure salt crystals (CaCl_2_, CuCl_2_ and CuCl) and 99.9% pure Ca metal were used as the reference compounds to compare with those dried Ca–Cl@rGO and Cu–Cl@rGO membranes in NEXAFS experiments. Due to its high-sensitivity to oxygen, the pure Ca metal was carefully handled in a glove box filled with highly pure Ar gas (O_2_ and H_2_O concentration were less than 0.1 ppm) to minimize oxidation.

**Conductive AFM data collection**

Topography and conductive measurements of the pure graphene sheets and dried Ca–Cl@graphene sheets were obtained with a commercial atomic force microscopy (AFM) system (Asylum Research Cypher of Oxford). Here, a conductive Pt coated probe was used as a conductive AFM probe. Current was measured at bias voltage rang of 0.05–2 V applied to the Au coated quartz substrates.

**Electrical resistivity measurement**

Three dried GO, rGO, Ca–Cl@GO, and Ca–Cl@rGO membranes with the thickness of ~0.1 mm and diameter of ~15 mm were prepared, respectively. Then the electric resistivities of five selected positions of each dried membrane were measured by using the multimeter with two electrodes (~24 mm^2^) connecting with the up and down surfaces of the membranes under ambient conditions. During the measurement, each position was exerted the same pressure in the perpendicular direction of the membranes. All the measurements were performed in a glove box filled with highly pure Ar gas (O_2_ and H_2_O concentration were less than 0.1 ppm).

**Heterojunction behavior measurement**

The rectification behavior of the dried Ca–Cl@rGO and pure dried rGO membranes was measured with a SourceMeter system (part number 2636B, Keithley). Corresponding current–voltage was measured at a bias voltage of 5V applied to two Cu electrodes (size of ~16 mm^2^) connecting with the up and down surface of the membranes under ambient conditions.

**Piezoelectricity-like property measurement**

For each measurement, the dried Ca–Cl@rGO membrane or pure dried rGO membrane was connected by two Cu foil electrodes and fixed by two flexible plastic plates. The voltages between the top and bottom of the membranes was measured by a SourceMeter system (part number 2636B, Keithley) when the two plastic plates were bent to a certain angle.

**Room-temperature ferromagnetism measurement**

The magnetic properties of the dried Ca–Cl@rGO membrane and pure dried rGO membranes with respect to temperature and field were measured using a quantum design MPMS-SQUID VSM-094 magnetometer (sensitivity ~1×10^−8^ emu). Before membrane samples were tested, the membranes were cut into rectangular pieces (about 3 mm × 2 mm), which were weighed by using a microbalance. Then the samples were loaded into the quartz holder (provided by Quantum Design) and wrapped, after which the quartz holder was attached to the sample rod and inserted into the magnetometer. Magnetic hysteresis curves of the pure dried rGO membranes and dried Ca–Cl@rGO membranes were measured at room-temperature (300 K) and low-temperature (5 K) in the field range of −40 kOe < H < +40 kOe.

**Hydrogen storage capacity measurement**

Before membrane samples were tested, the pure dried rGO membranes and dried Ca–Cl@rGO membranes were cut into small pieces respectively. The hydrogen adsorption and desorption isotherms were measured using a surface area and porosity analyzer (ASAP 2020) while sample cell was submerged in liquid nitrogen bath (77 K). The testing relative pressure (p/p_0_) was set to a range of 0 to 1.

The dried testing cell with samples was degassed at 343 K for 6 h under dynamic vacuum to remove surface-adsorbed gases. The weight of the sample according to the difference between the net weight of the sample cell and the total weight of the sample cell after degassing is approximately 100 mg. When the sample cell cooled down to room temperature, nitrogen was injected into the cell. Subsequently, the pure hydrogen (purity > 99.999%) was injected in the test system to measure the hydrogen adsorption capacity at a constant temperature of ~77 K with increasing relative pressure (p/p_0_) from 0 to 1. Similarly, desorption of absorbed hydrogen was allowed to occur with the declining pressure maintained at 77 K. Each data of weight is tested three times and then averaged.

**Theoretical computation methods:**

**Crystal structure prediction, optimization and electronic property calculations**

To predict and search stable structures of the 2D CaCl crystals, the evolutionary algorithm implemented in the USPEX package [21] were explored. During structure searing, the atomic ratio between calcium, chlorine and carbon is set to be 1:1:8 suggested by the experimental element analyses. Each supercell contains a vacuum layer of 10–15 Å. 40 populations were generated randomly in the initial generation, then the energetically worst structures (40%) were discarded, and a new generation was created including 30% randomly generated initial structures and 70% from the lowest-enthalpy structures through the evolution operations. At last, 22 generations and more than 456 structures are explored in the entire search. Structure relaxations were performed in the VASP code (See the VASP section for details).

First principles calculations based on the spin-polarized DFT were performed with the Vienna ab initio Simulation Package (VASP) code [34]. The Projector Augmented Wave (PAW) pseudopotentials were applied [35,36]. In order to investigate the structural properties, the generalized gradient approximation (GGA) of the Perdew–Burke–Ernzerhof (PBE) was used to treat the exchange-correlation interaction between electrons [37]. A plane-wave basis set with a kinetic-energy cut-off of 520 eV was used to expand the wave function of valence electrons (2s^2^p^2^ for C, 3s^2^p^5^ for Cl and 3p^6^4s^2^ for Ca). Three-dimensional periodic boundary conditions were applied to simulate the infinite systems. A 20 Å vacuum space between sheets was set to prevent the interaction between two layers. The structural relaxations were performed by computing the Hellmann-Feynman forces within total energy and force convergences of 10^−5^ eV and 10^−3^ eV/Å, respectively. The van der Waals interactions were introduced in the calculating, which was described by a correction through the Grimme’s zero damping D-3 method [38]. Gamma-centered Monkhorst-Pack grids of 15 × 15 × 1, 15 × 15 × 11 and 3 × 3 × 1 were used for models I/II, stacked model I and models III-IX, respectively [39]. When investigating the electronic properties (band structures, density of states and charge density), more dense grids of 21 × 21 ×1, 21 × 21 ×15 and 5 × 5 × 1 were employed. In band structures calculations, the Gaussian smearing with broadening of 0.1 eV was used for partial occupancy, while the density of states and charge density were calculated by the tetrahedron method with Blöchl corrections.

**Thermodynamic stability analysis of the Ca–Cl crystal structures**

All ab initio molecular dynamics (AIMD) simulations were performed using VASP code [34], starting from the relaxed structures obtained by DFT calculations, with no initial velocities assigned. The size of supercells used in simulations are 14.77 × 17.07 Å^2^ for model I/II and 12.35 × 12.82 Å^2^ for model III–IX. A 20 Å vacuum space between sheets is set to prevent the interaction between two layers. The generalized gradient approximation (GGA) of the Perdew-Wang 91 (PW91) was used to treat the exchange-correlation interaction of electrons [40]. Monkhorst-Pack grids of 3 × 3 × 1 was used to sample the Brillouin zone. The simulations were carried out in a canonical ensemble (NVT) with a Nosé thermostat for temperature control [41]. The temperature was constant at 300 K by lasting for 15 ps with time step of 1 fs.

**Bader charge analysis**

Population analysis was performed using the Bader Charge Analysis code developed by Henkelman’s group [42-44]. Most charge analysis methods such as Mulliken population analysis were based on electron wave functions and thus sensitive to the type and cutoﬀ of basis sets. Bader analysis [20], on the other hand, makes use of zero ﬂux surfaces and distinguish the ownership of electrons. The charge enclosed within the Bader volume is a good approximation to the total electronic charge of an atom and the analysis was merely dependent on charge density distribution [20]. In this work, charge density distribution was calculated from VASP [34].

**Electrical conductivity calculation**

The all-electron LAPW (linearized augmented plane wave) WIEN2K package [45] was used for the electrical conductivity calculations, with the PBE-GGA exchange-correlation potential. All simulations are at 42 × 42 × 1 k-grids for 2D models and at 42 × 42 × 27 k-grids for 3D models. The cut-off energy to separate the core electrons from the valence electrons is −6.0 Ry. RmtKmax was set to 7.0 (where Rmt is the smallest atomic sphere radius and Kmax is the maximum number of wave vectors being used) and GMAX was set to 12.0. After converged DFT iterations, the electrical conductivity was derived from the linear optical conductivity by random-phase approximation [46]. The convergence of plasma frequencies had been checked for all 2D and 3D models.

**Piezoelectricity-like property calculation**

DFT computations were performed using the VASP code^6^ to estimate the piezoelectricity-like property of the system for the strain perpendicular to the graphene plane induced by external stress during bending process. For the selected crystal model, Cl was moved with a step size of Δz_0_ = 0.033 Å along the *z*-direction to simulate strain perpendicular to the graphene plane, and the *z*-coordinate of C atoms was fixed. The pseudopotentials, approximation, basis set, vacuum space and convergence accuracy of energy and force are the same as we used in the calculations of electronic properties. Rectangular cell (2 fold supercells of model I) was selected and Monkhorst-Pack grids were set as 15 × 11 × 1.

**Ferromagnetism and antiferromagenetism calculation**

The spin polarized calculations of CaCl (up and down) of CaCl crystals with model I configuration were performed by VASP to explore the magnetic property of the system. In addition, 2×2×1 and 1×2×1 supercells of model I were constructed to investigate the antiferromagnetism by assigning opposite initial magnetic moments in adjacent unit cells. The pseudopotentials, approximation, basis set, vacuum space and convergence accuracy of energy and force are same as *electronic property calculations.* Monkhorst-Pack grid of 15 × 15 × 1, 7 × 7 × 1 and 15 × 7 × 1 were used to sample the Brillouin zone of unit cell, 2×2×1 and 1×2×1 supercells, respectively.

Edge effect induced magnetism was calculated for CaCl clusters on periodic graphene substrates. Brillouin zone is sampled using only the Γ k-point. In addition, clusters on graphene substrate is used to simulate the one-dimensional periodic condition. Monkhorst-Pack grids of 9 × 3 × 1 and 5 × 3 × 1 were used to sample the Brillouin zone of single cell (4.95 × 12.86 Å^2^) and supercell (9.90 × 12.86 Å^2^). Defect-induced magnetism was calculated in a 14.85 × 17.14 Å^2^ supercell with model I configuration, and Monkhorst-Pack grid of 3 × 3 × 1 was used to sample the Brillouin zone. The structural relaxations were performed by computing the Hellmann-Feynman forces within total energy and force convergences of 10^−4^ eV and 0.05 eV/Å, respectively.

**Hydrogen storage capacity calculation**

DFT computations were performed with the VASP to explore the potential capacity of the system for hydrogen storage. The calculations of adsorption energy were performed on a 2 × 2 supercell of model I. Monkhorst-Pack grid of 5 × 5 × 1 was used to sample the Brillouin zone. While for the system of hydrogen storage in stacked model I, grid of 9 × 9 × 7 was employed. In the AIMD calculations, a 2 × 2 × 2 supercell of stacked model I was used. Monkhorst-Pack grid of 5 × 5 × 3 was used to sample the Brillouin zone. The temperature was increased linearly from 0 to 300 K in 6 ps with time step of 2 fs. In the intermediate period a micro-canonical ensemble was simulated.

**PS2: Structure determination of Ca–Cl crystals by cryo-EM, conventional TEM and XPS**

A summary of models we studied with their properties are listed in the following table together with corresponding experimental observations.

**Table S1. A summary of crystal models and their electronic properties, together with corresponding experimental observations.**

| Models | TEM  Observations | Atomic Structure | Band Structure | Charge Transfer | Metallic |
| --- | --- | --- | --- | --- | --- |
| I (CaCl)* | Fig. 1  Figs. S1/S2/S3 | Fig. 1  Fig. S11a | Fig. 1  Fig. S14/S18 | Fig. S15 and Table S3 | Y |
| II (CaCl) | Fig. 1  Figs. S1/S2/S3 | Fig. S11a | Fig. S14 | Fig. S15 and Table S3 | Y |
| III (CaCl) | NA | Fig. S11a | Fig. S19 | NA | Y |
| IV (CaCl_4/3_) | NA | Fig. S11b | Fig. S19 | NA | Y |
| V/VI (CaCl_2/3_) | NA | Fig. S11b | Fig. S19 | NA | Y |
| VII-IX (CaCl_2_) | NA | Fig. S11c | Fig. S20 | NA | N |

* Including stacked model I.

Fig. S1a shows the survey TEM view of the frozen wet ultra-thin Ca–Cl@rGO membrane, in which the ultra-thin membrane is stably supported by the holey carbon film. In the ultra-thin Ca–Cl@rGO membranes, a stable single-crystal diffraction pattern was observed (Fig. S1b). High-resolution cryo-EM images show that such crystals have a lattice spacing of 4.29±0.11 Å (Fig. S1c), corresponding to a graphene-like honeycomb lattice with a side length of 2.86±0.13 Å. Electron diffraction and fast Fourier analyses [13] of the Ca–Cl lattice yielded a hexagonal lattice with first-order maximal points, i.e., the (1−100) reflections, at (1±0.02)/4.29 Å^−1^. In Fig. S1b, we can also see that there are about five sets of hexagonal diffraction spots at ~1/2.13 Å^−1^, which correspond to the (1−100) reflections of the graphene. Notably, the (2−200) reflections of the Ca–Cl crystals are coincident with these (1−100) reflections of graphene (Fig. S1b).

Double-orientated Ca–Cl crystals together with the graphene sheets with several orientations were also observed (Fig. S2). We can see that the lattice spacing of the six first-order maxima of Ca–Cl crystals at ~1/0.43 nm^−1^ are twice the lattice spacing of the six first-order maxima of the graphene (~1/0.21 nm^−1^). The high-resolution cryo-EM image of the double-orientated Ca–Cl crystals (Fig. S2b) shows a clear lattice structure with a Moiré pattern, which is induced by these two differently orientated Ca–Cl crystals. The double orientations of Ca–Cl crystals can be further observed by the corresponding FFT pattern (inset of Fig. S2b). Further, Fig. S3a shows a high-resolution cryo-EM image of the Ca–Cl crystals with multiple orientations in an ultra-thin Ca–Cl@rGO membrane. The FFT pattern of the high-resolution cryo-EM image (Fig. S3b) displays six first-order maxima of the honeycomb lattice at ~1/0.43 nm^−1^, which is consistent well with twice the lattice spacing of the graphene.


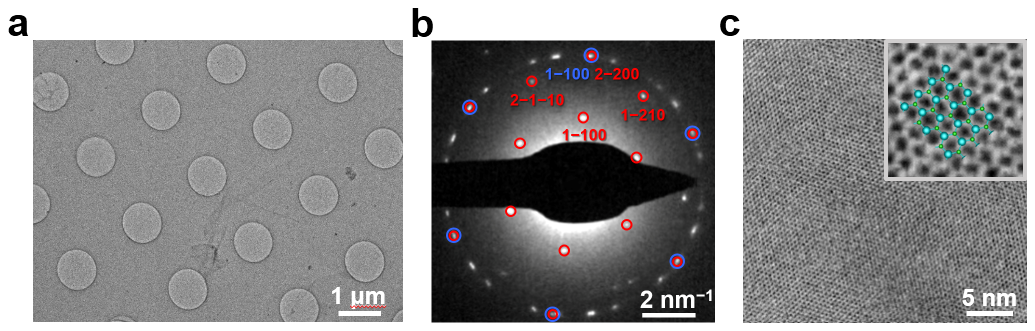


**Fig. S1. Cryo-EM images of the Ca–Cl crystal in the ultra-thin Ca–Cl@rGO membrane at 80-kV high-tension. a**, Survey view of the ultra-thin Ca–Cl@rGO membrane on holey carbon film. **b**, A typical diffraction pattern of the frozen wet ultra-thin rGO membrane. The (1−100) reflections of graphene (blue) coincide with the ring formed by the (2−200) reflections of the CaCl crystal (red), indicating that the single-orientated CaCl structure coincides with twice the lattice spacing of the graphene. **c**, A typical high-resolution cryo-EM image of the CaCl crystals in the target area. The inset shows a zoomed-in area of the high-resolution image with the CaCl crystal structure from the molecular model I, in which Ca and Cl atoms are in cyan and green, respectively.


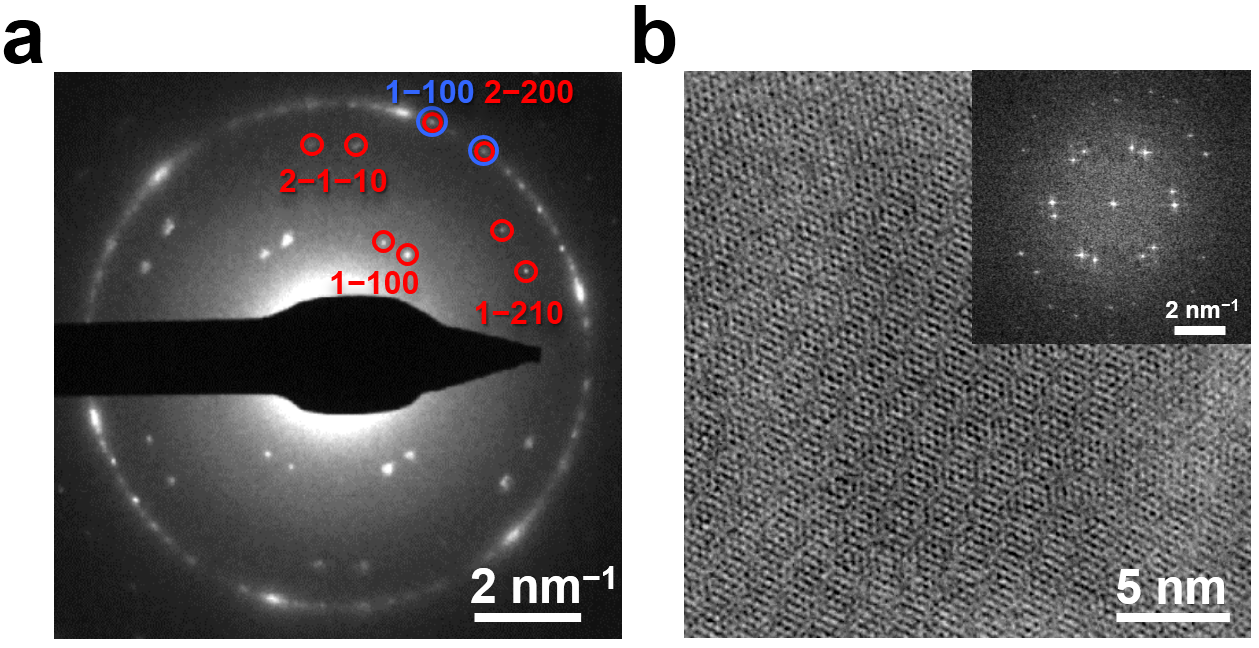


**Fig. S2. Diffraction pattern and high-resolution cryo-EM image of the Ca–Cl crystals with double orientations in a frozen wet ultra-thin Ca–Cl@rGO membrane at 80-kV high-tension. a**, A typical diffraction pattern of the frozen ultra-thin rGO membrane. The (1−100) reflections of the graphene sheets and the Ca–Cl crystals are indicated by the blue and red dashed lines, respectively. **b,** A typical high-resolution cryo-EM image of the Ca–Cl crystals with double orientations in the target area, showing a typical Moiré pattern. The FFT pattern of the high-resolution cryo-EM image (inset) shows that the first-order maxima are at ~1/0.43 nm^−1^.


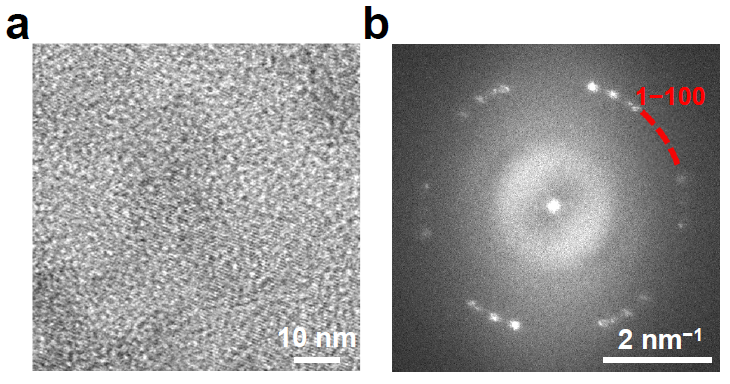


**Fig. S3. Cryo-EM images of the Ca–Cl crystals with multiple orientations in a frozen wet ultra-thin Ca–Cl@rGO membrane at 80-kV high-tension. a,** A typical high-resolution images of the Ca–Cl@rGO membrane. **b,** The corresponding FFT pattern of the high-resolution cryo-EM image. The first-order maxima are at ~1/0.43 nm^−1^, corresponding to the structure of a Ca–Cl crystal with six first-order maxima of the honeycomb lattice at ~4.26 Å. The (1−100) reflections of the Ca–Cl crystal are indicated by a red dashed line.

To further confirm the Ca–Cl crystals observed by cryo-EM, the dried thick Ca–Cl@rGO membrane was also examined by conventional (non-cryogenic) TEM. As shown in Fig. S4, same honeycomb lattice with first-order maxima at ~4.3 Å can be observed in the areas having few-layer and multi-layer rGO sheets of this sample. This suggests that such Ca–Cl crystals are not induced by the flash-frozen process. Fig. S4 also shows that the (2−200) reflections of those six typical Ca–Cl crystals are consistent well with the (1−100) reflections of the graphene sheets. We have also note that other crystals could also be imaged though they have much smaller chances to be detected. Two typical such other ones are displayed in Figs. S5 and S6, respectively.


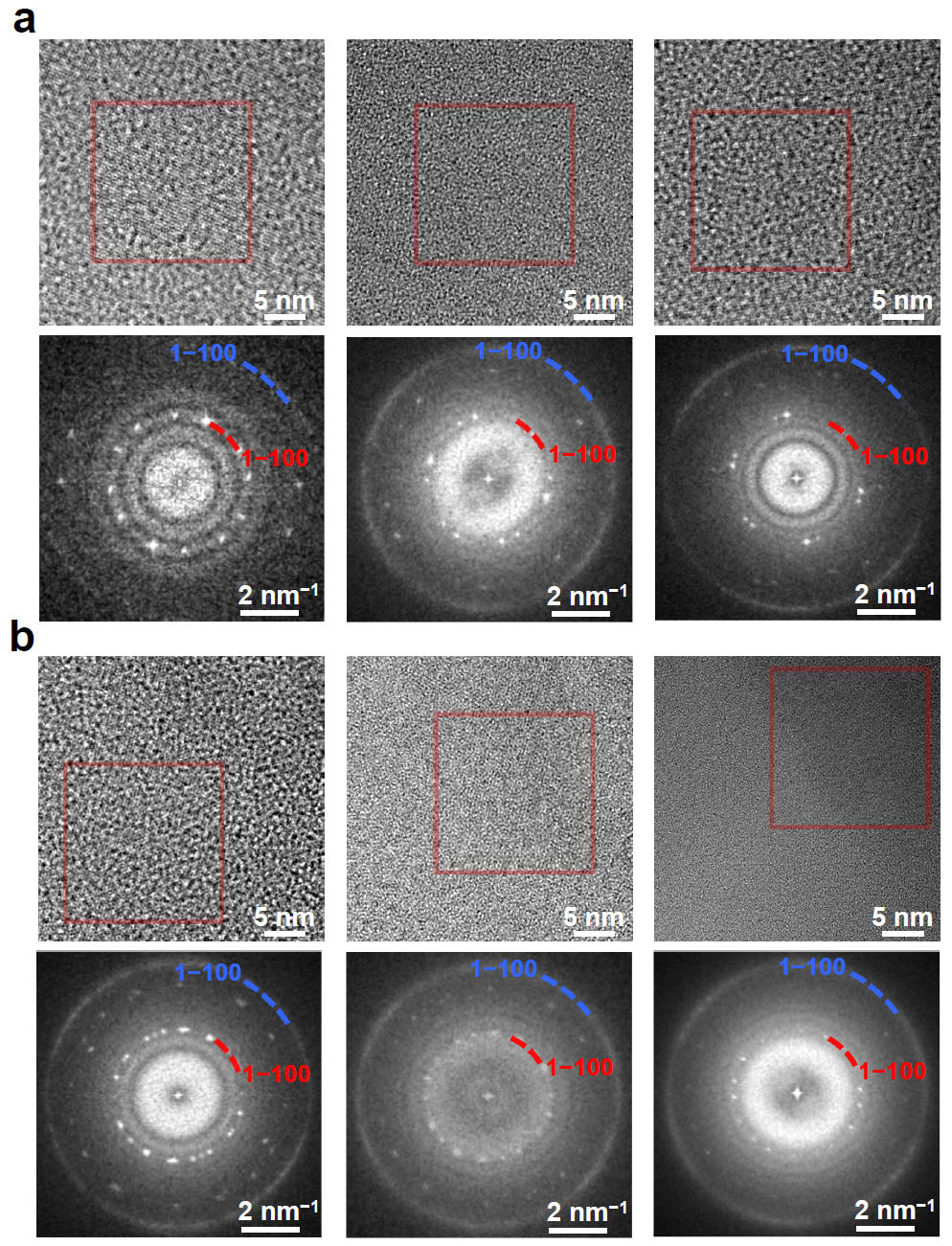


**Fig. S4. Conventional TEM images of the Ca–Cl crystals with double and multiple orientations at the edge of the dried Ca–Cl@rGO membrane without frozen process at 200-kV high-tension. a**, Areas of the CaCl crystals with double orientations, as indicated by the two hexagonal lattice patterns with six first-order maxima points at ~1/0.43 nm^−1^ and different in-plane rotation angles. **b**, Areas of the CaCl crystals with multiple orientations, as indicated by the multiple hexagonal lattice patterns with six first-order maxima points at ~1/0.43 nm^−1^ and different in-plane rotation angles. The top images are the raw images, and the bottom images are the corresponding FFT patterns. The FFT patterns show the lattice relation between the graphene (blue) and the CaCl crystals (red).


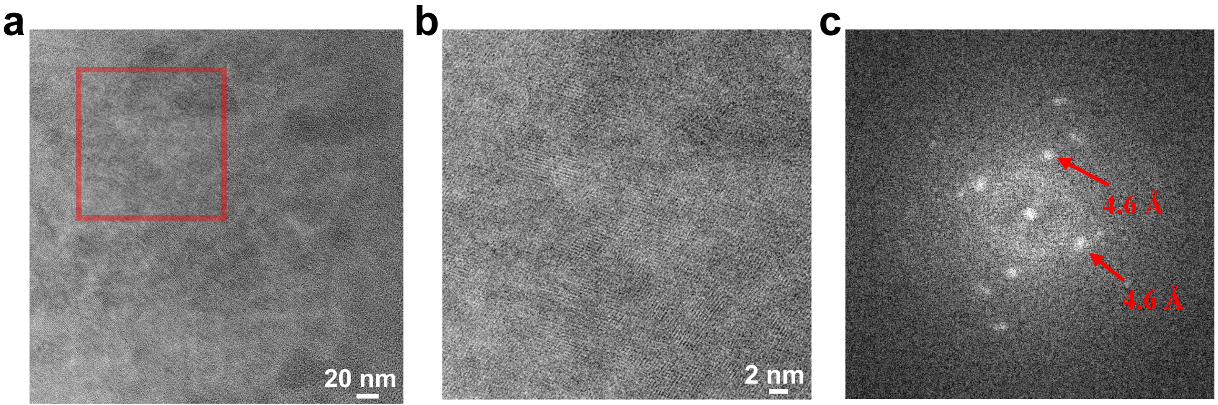


**Fig. S5. Conventional TEM images of another typical Ca–Cl crystal at the edge of the dried Ca–Cl@rGO membrane without frozen process at 200-kV high-tension. a**, Survey view of the edge region and selected area for high-magnification imaging. **b**, High-magnification image of the selected area. **c**, The FFT of the high-magnification image, showing first-order maxima of a square lattice at ~4.6 Å.


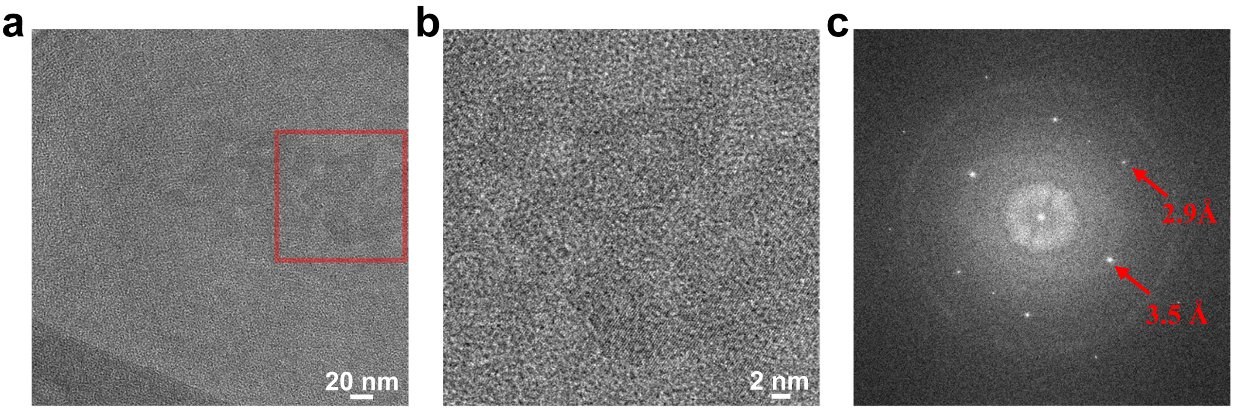


**Fig. S6. Conventional TEM images of a third typical Ca–Cl crystal at the edge of the dried Ca–Cl@rGO membrane without frozen process at 200-kV high-tension. a**, Survey view of the edge region and selected area for high-magnification imaging. **b**, High-magnification image of the selected area. **c**, The FFT of the high-magnification image, showing first-order maxima of lattice at ~3.5 Å and ~2.9 Å.

We used energy-dispersive X-ray spectroscopy with transmission electron microscopy (EDS–TEM) to further investigate the element contents in the dried Ca–Cl@rGO membranes. As shown in the dark-field HADDF-STEM image in Fig. S7, the atomic ratios of Ca to Cl are from ~1:2 to 1:1, where the ratios in bright areas are close to 1:2 while in dark areas are close to 1:1. EDS result also suggests that there are no other elements than C, O, Ca and Cl in such areas (Fig. S7c). Here an area is called bright (dark) when the image intensity in this area is higher (lower) than the average intensity of the whole membrane region. Generally, the higher the intensity, the thicker the membrane. Thus, the atomic ratios of Ca to Cl are close to 1:2 in the thick membrane while close to ~1:1 in the thin membrane. We think that there are more regular CaCl_2_ crystals in the thick membrane due to solution evaporation so that the ratios are close to 1:2. In contrast, in the thinner areas there are less CaCl_2_ crystals and most of the crystals are the abnormal Ca–Cl crystals, which have the ratio close to 1:1.

To further investigate the elemental contents in larger areas, we used X-ray photoelectron spectroscopy (XPS, with an analysis area ~0.36 mm^2^, of the dried Ca–Cl@rGO membrane) and inductively coupled plasma (ICP, for multiple dried Ca–Cl@rGO membranes) to investigate the full elemental contents in the dried membranes. As shown in Fig. S8, XPS analysis shows that the atomic percentages of the main elements inside the dried Ca–Cl@rGO membrane are 83.3%, 6.9%, 4.4%, and 4.8% for C, O, Cl, and Ca respectively, while the only other detectable element is S (<0.7%). And by etching with argon ions, the depth profile shows that the Ca:Cl ratios vary from a value below 0.6:1 at the membrane top surface to ~1.1:1 in inner sheets. ICP analysis of multiple membranes shows that in the dried Ca–Cl@rGO membranes the mass percentage of Ca, Fe, and Mn are ~5.5%, ~0.2%, <0.1%, respectively, and there is negligible value of Ni (Fig. S9). In the pure dried rGO membranes very close mass percentages of Fe (0.2%) and Mn (<0.1%) as those in the dried Ca–Cl@rGO membrane. We think that the small amount of calcium (~1.1%) appeared in the pure rGO membranes were introduced by the added solvents while performing ICP measurements. As we have used the same protocols for both of the dried Ca–Cl@rGO and dried rGO membranes, the difference of the calcium content should be the mass percentage of calcium in the dried Ca–Cl@rGO membrane, i.e., the dried Ca–Cl@rGO membrane has ~4.7% calcium in weight.

Regular CaCl_2_ crystals could be observed in the dried Ca–Cl@rGO membranes which may be resulted by the evaporation process of CaCl_2_ solution coated on the membrane surface (Fig. S10) since the ratios of the Ca and Cl elements in the solution are always comparable (~1:2) due to the electronic balance.


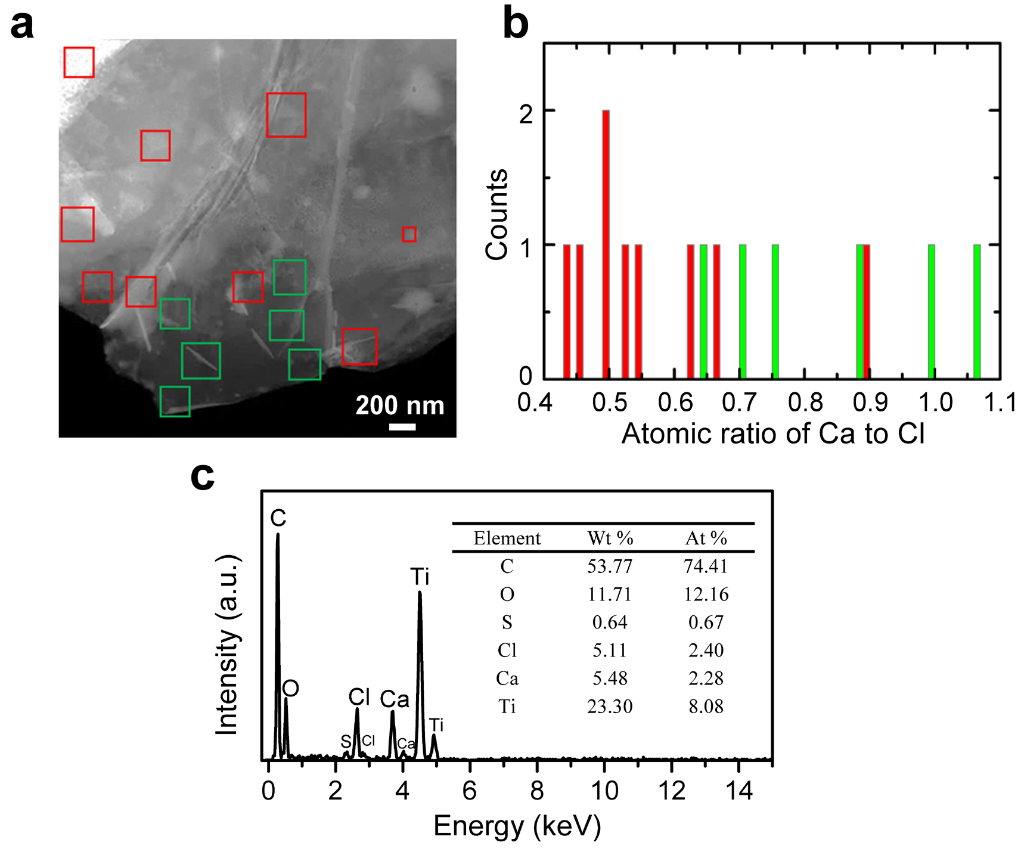


**Fig. S7. Elemental analysis of a region from the dried Ca–Cl@rGO membrane by EDS. a**, HADDF-STEM image of this region and selected areas for quantitatively elemental analysis by using EDS. Thicker areas (image intensity higher than the average intensity of the whole membrane region) are labeled in red, while thinner areas (image intensity lower than the average intensity of the whole membrane region) are labeled in green. **b**, Histogram of the atomic ratio of Ca to Cl measured in these 15 areas. **c**, Full elemental analysis of a region (~0.2 μm^2^) in the dried Ca–Cl@rGO membrane. The Ti peaks come from the Ti grid.


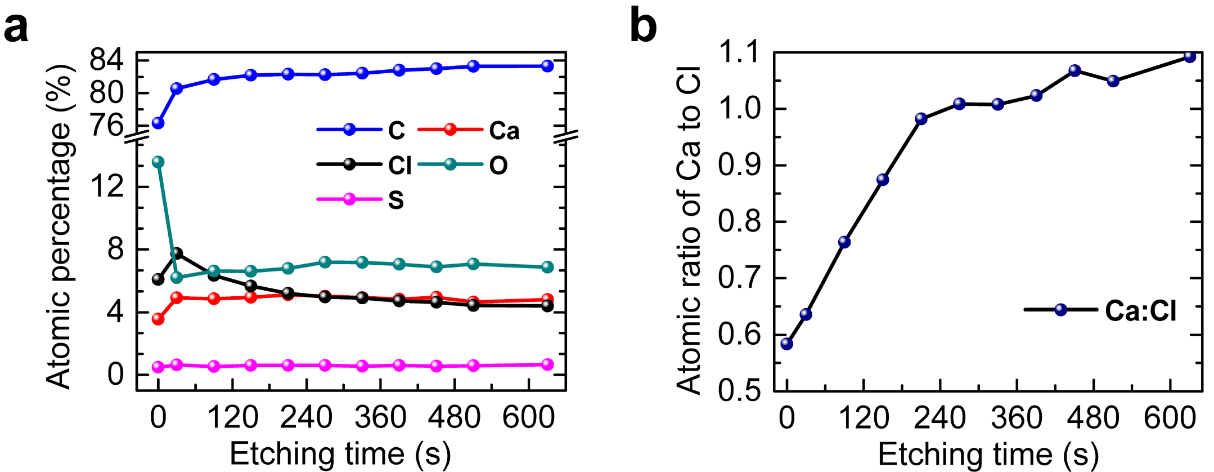


**Fig. S8. Full elemental analysis of a region (~0.36 mm^2^) from the dried rGO membrane by XPS.** **a**, XPS analysis showing that the atomic percentages of the main elements inside the Ca–Cl@rGO membrane are 83.3% (C), 6.9% (O), 4.4% (Cl), and 4.8% (Ca), respectively, while the only other detectable element is S (<0.7%). **b**, The atomic ratio of Ca to Cl as a function of the etching time. By etching with argon ions, the depth profile shows that the Ca:Cl ratios vary from a value below 0.6:1 at the membrane top surface to ~1.1:1 in inner sheets.


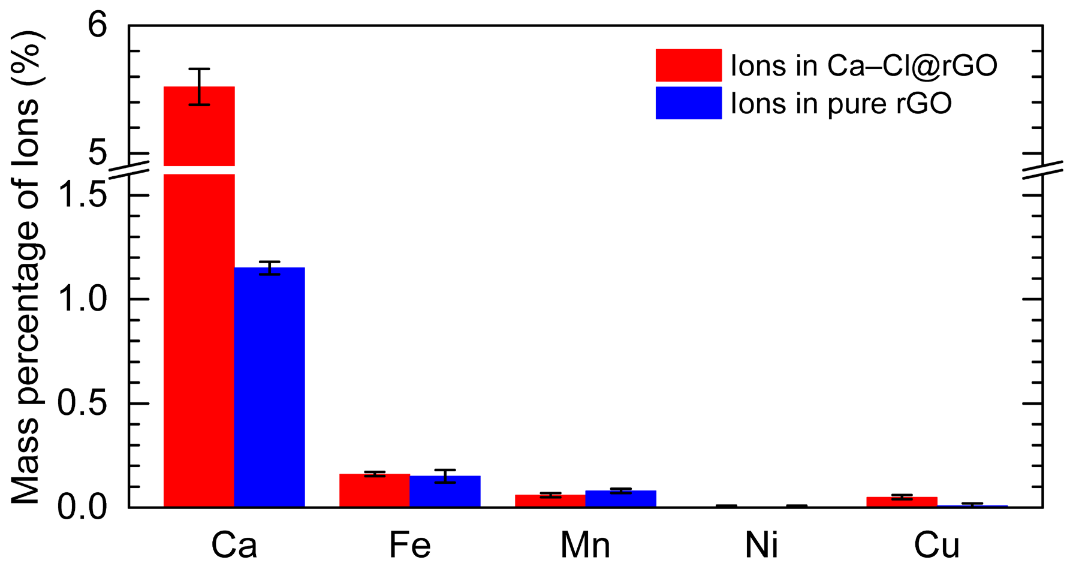


**Fig. S9. Full elemental analysis of the dried rGO membranes (~66.6 mg) and pure dried rGO membranes (~8.2 mg) by ICP.** In the dried Ca–Cl@rGO membranes, the mass percentage of Ca is ~5.8%, that of Fe is ~0.4%, that of Mn is <0.1%, and there is negligible Ni. The pure dried rGO membranes have very close mass percentages of Fe (0.2%) and Mn (<0.1%) as those in the dried Ca–Cl@rGO membrane.

**
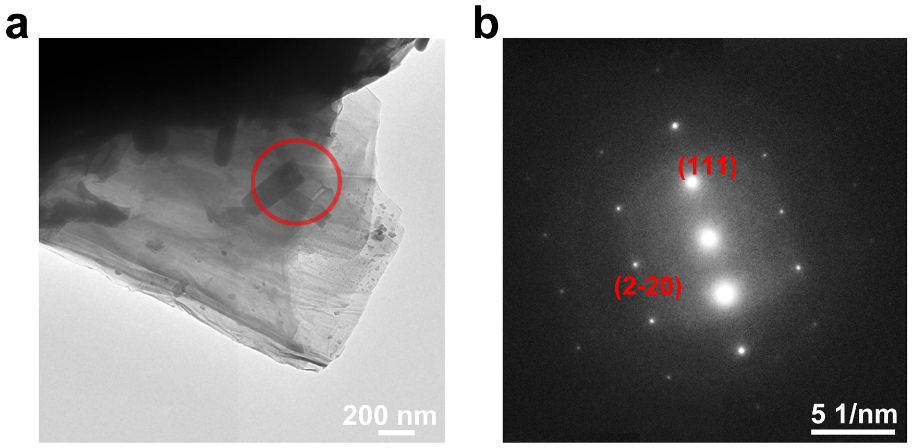
**

**Fig. S10. Conventional TEM images and SAED of a typical CaCl_2_ monocrystal in the dried rGO membrane observed at 200-kV high-tension.** **a**, Survey view of the edge region. **b**, SAED pattern of the selected area covered by the red circle in (a) shows a regular CaCl_2_ monocrystal.

**PS3: Structure modelling of the Ca–Cl crystals by DFT and USPEX**

We built 21 initial Ca–Cl crystal modules adsorbed on a graphene sheet or confined between two graphene sheets. After geometric optimization, we found 9 stable crystal structures, as shown in Fig. S11.


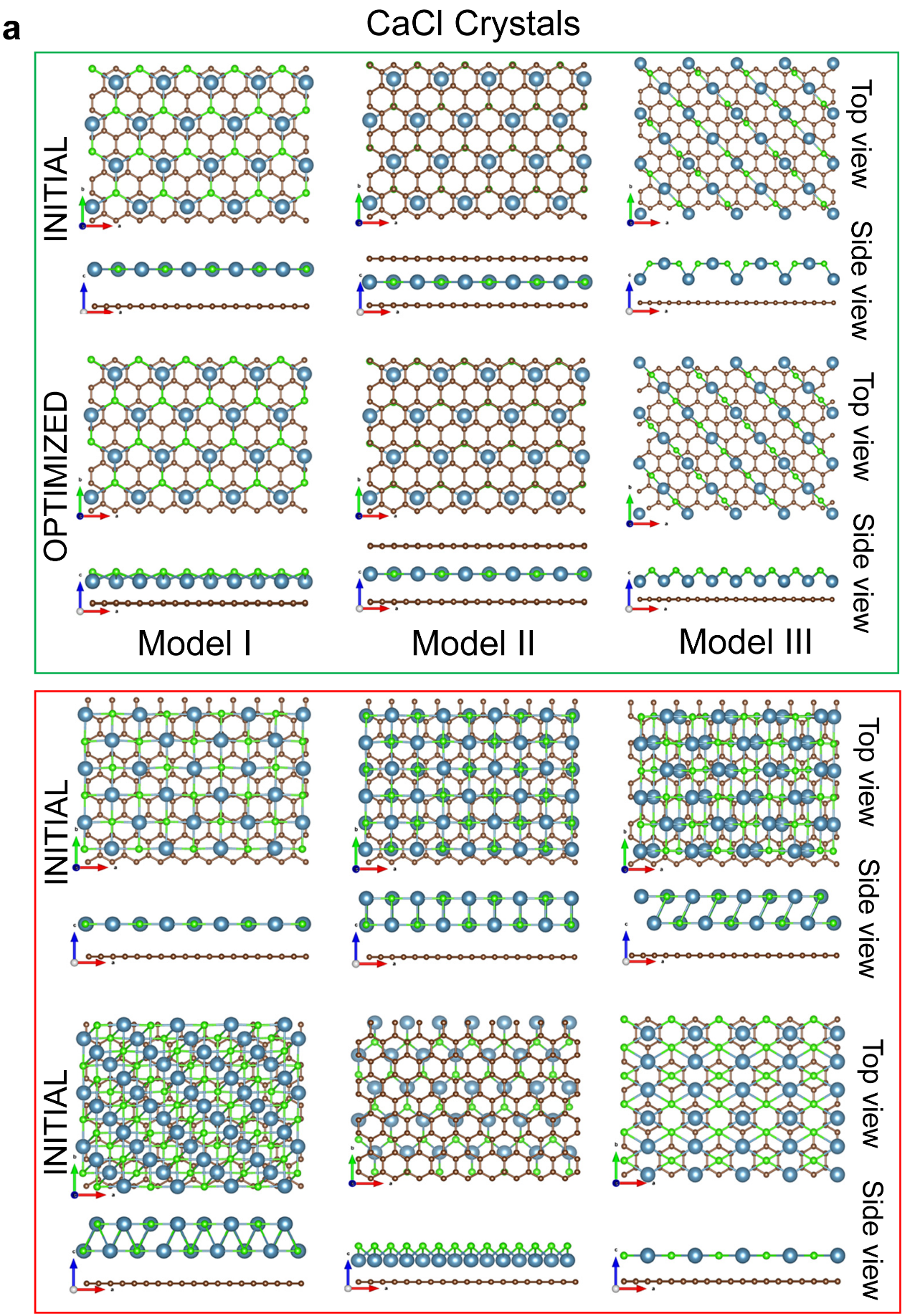


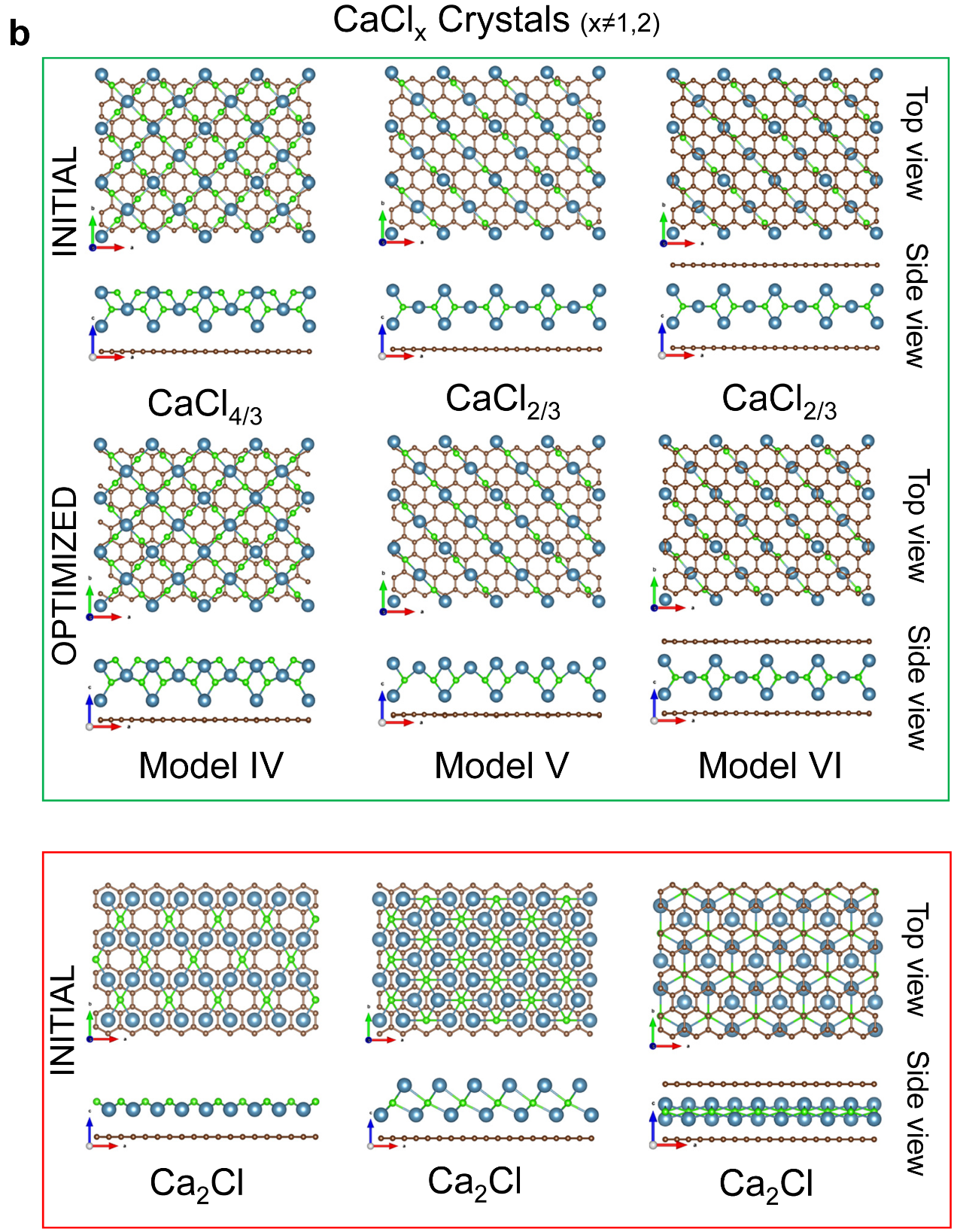


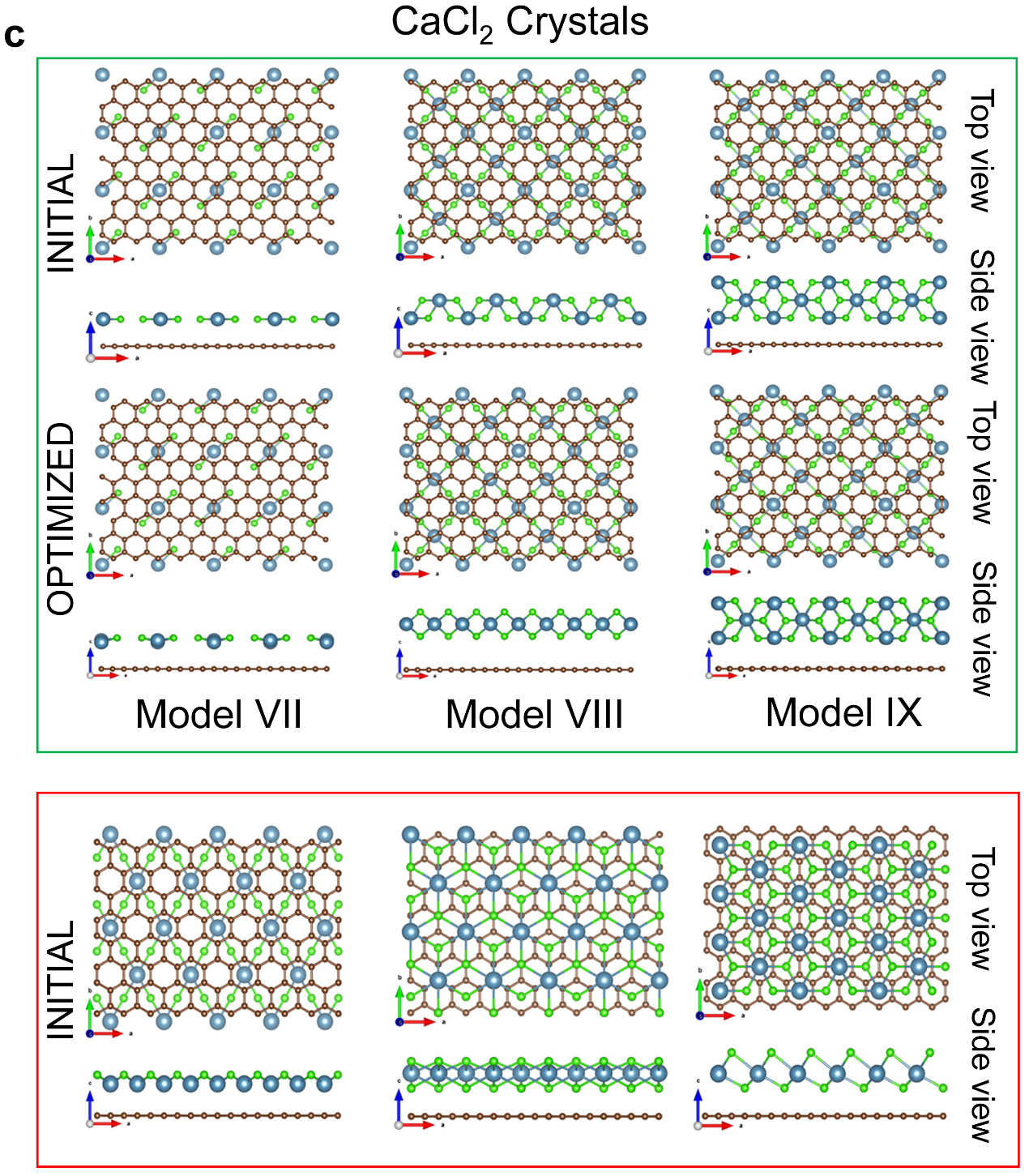


**Fig. S11.** **a**-**c**, Initial Ca–Cl crystal modules and optimized geometries of these Ca–Cl crystal structures. In the green boxes the initial structures are shown at top, and the optimized stable structures are shown at bottom, while for all of the initial structures in the red boxes there are no stable structures found after geometry optimization.

To further validate these models for the observed structures, we have used the USPEX [47] algorithm (a common method in the field of crystal structure prediction) incorporated with density-functional theory (DFT) calculations to predict the stable structures of materials. The five most stable structures are shown in Fig. S12. The most stable structure (structure 1) from 456 initial structures is just the model I, which is consistent with our experimental observations that the most frequently observed structure in TEM experiments having the same lattice constant is model I. Structure 2 has the same crystal constant as structure 1, and our AIMD simulation shows that this structure can easily transform to structure 1 by thermal fluctuations under ambient conditions (Fig. S13). Structures 3–5 correspond to the irregular graphene configurations of pentaheptite, pentahexoctite, and T-graphene, respectively. We emphasize that the carbon substrates of structures 1 and 2 are still the regular graphene configuration. Hence, structures 1 and 2 predicted by USPEX are consistent with the structures obtained by the DFT method, in which only the pristine graphene configuration was considered as the substrate.


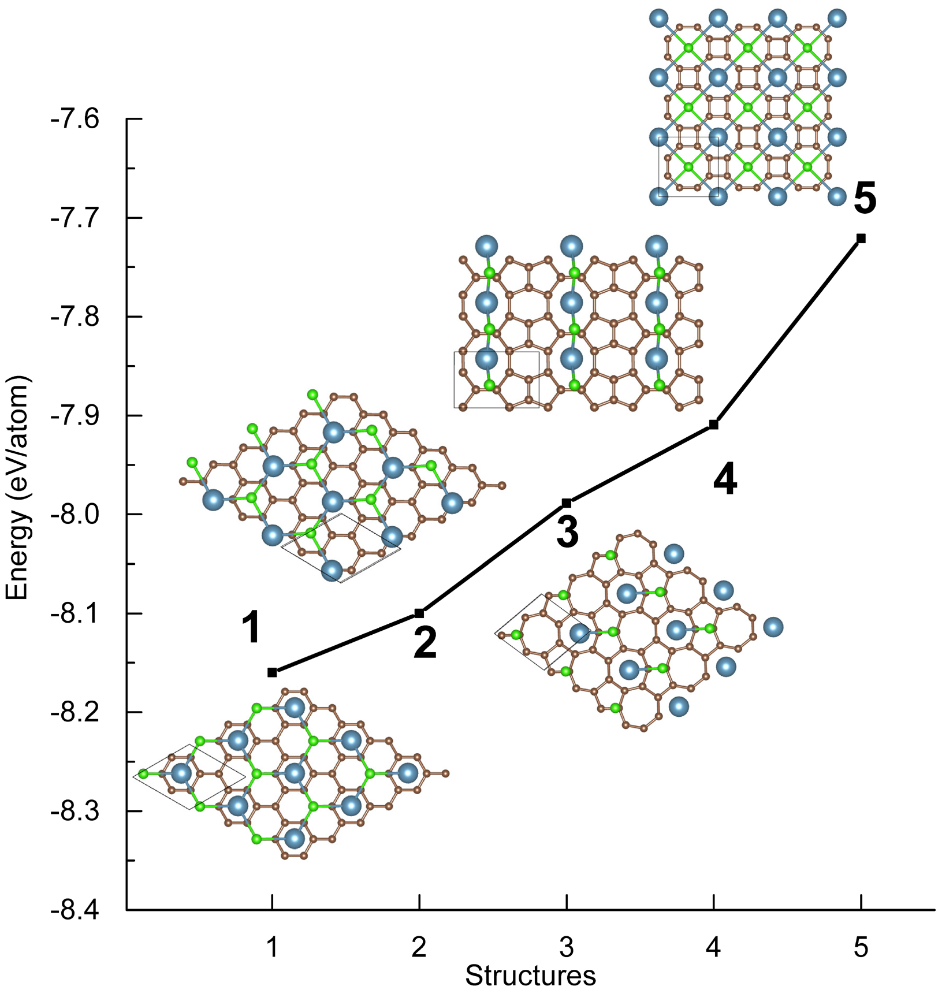


**Fig. S12. Five most stable structures and the corresponding total energies from 456 random initial structures predicted by USPEX.** Structures 1 and 2 have the same lattice constants (*a* = 4.92 Å, *b* = 4.92 Å, and α_ab_ = 60.0°) but different Ca and Cl positions and the carbon substrate has the standard graphene configuration. The lattice parameters of structure 3 are *a* = 4.79 Å, *b* = 4.79 Å, and α_ab_ = 76.5°, and the carbon substrate is the pentaheptite configuration. The lattice parameters of structure 4 are *a* = 5.85 Å, *b* = 3.87 Å, and α_ab_ = 90.0°, and the carbon substrate is the pentaheptite configuration. The lattice parameters of structure 5 are *a* = 4.88 Å, *b* = 4.88 Å, and α_ab_ = 90.0°, and the carbon substrate is the T-graphene configuration.


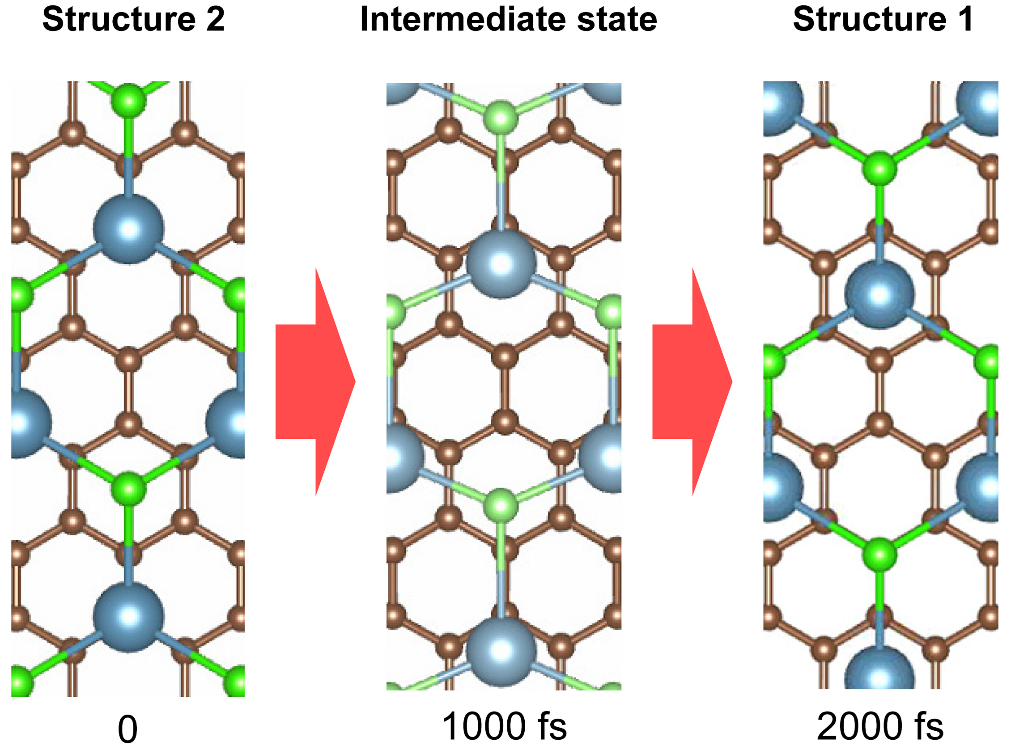


**Fig. S13. Spontaneous transition of structure 2 to structure 1 revealed by an ab initio molecular dynamic (AIMD) simulation performed at room temperature (300 K).** At 300 K, structure 2 transforms to structure 1 within 2000 fs and is then stable. From left to right, the simulated times are 0, 1000, and 2000 fs, respectively.

We note that model I (a single-layer of Ca–Cl adsorbed on a graphene sheet) and model II (a single-layer of Ca–Cl confined between two graphene sheets) have very similar in-plane lattice spacing (~4.3 Å) and lattice side length (~2.84 Å) as the Ca–Cl crystals observed by cryo-EM (Fig. 1 and Figs. S1–S3) and conventional TEM (Fig. S4). By comparing their theoretical diffraction spots with experimental diffraction patterns, the result shows very well consistency (Fig. S14a). Further ab initio molecular dynamic (AIMD) analysis was performed to study the stability of these Ca–Cl crystals in models I and II, and the Radial Distribution Functions show that there are no structural reorganization within 15 ps (Fig. S14b), suggesting these two models are stable under simulated ambient conditions. As the atomic ratio of Ca to Cl in models I and II is 1:1, we denote such crystals as 2D CaCl crystals.

To further study the stability of 2D CaCl crystals, we calculate their formation energy (eV/atom), which is defined as:

E_f_ = [E(A*_x_*B*_y_*) – *x*E(A) – *y*E(B)]/(*x+y*)

In which, E(A*_x_*B*_y_*) is DFT total energy of the compound A*_x_*B*_y_*, E(A)/E(B) is the chemical potential of element A/B, *x/y* is the quantity of element A/B in the compound. The standard convention is to take the chemical potential of each species to be the DFT total energy of the elemental ground state. Here, the Ca of Fm-3m and Cl of Cmca were selected. With this choice, the computed formation energy of CaCl from model I and II compared with several other calcium chloride compounds are listed in Table S2. All the structures of Ca, Cl, CaCl_2_, Ca_2_Cl, Ca_3_Cl and data with suffix * are from the Open Quantum Materials Database (OQMD). CaCl_2_ is the most stable in these compounds, while CaCl crystal from model I is more stable than Ca_2_Cl and Ca_3_Cl.

**Table S2. The formation energy of several calcium chloride compounds.**

| Compounds | CaCl from model I | CaCl from model II | CaCl_2_ | Ca_2_Cl | Ca_3_Cl |
| --- | --- | --- | --- | --- | --- |
| Formation energy per atom (eV/atom) | −1.138 | −1.061 | −2.436  −2.453* | −0.966  −1.041* | −0.242  −0.308* |

The electronic properties of models I and II were studied by calculating their band structures and density of states (DOS). As shown in Fig. S14c, both models exhibit distinct metallic properties, in which CaCl plays a significant role.


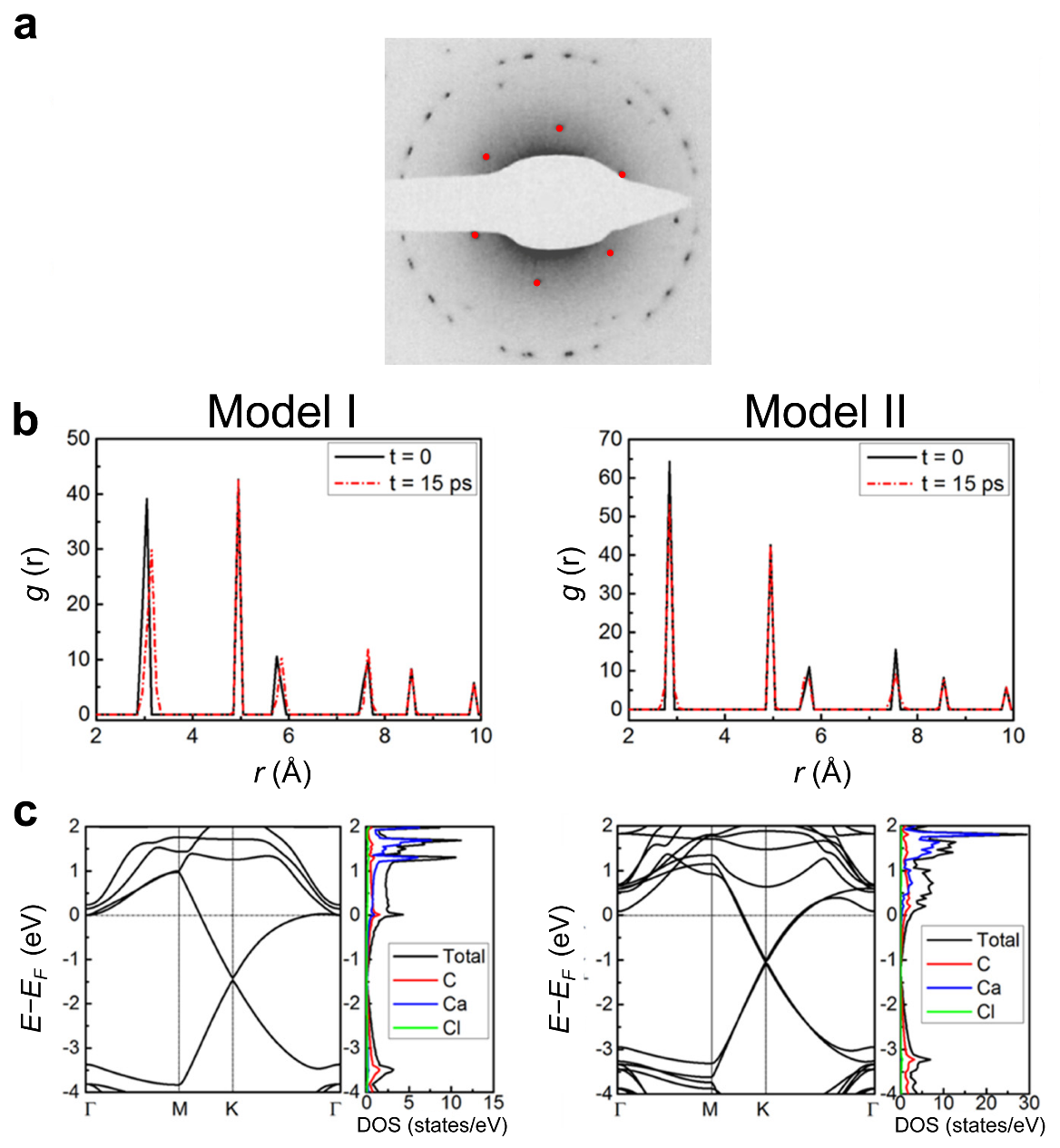


**Fig. S14. Geometric and electronic structure analysis of models I and II. a**, Theoretical diffraction spots of the CaCl crystals in model I and II, together with the experimental SAED pattern, are shown. **b** and **c**, Radial Distribution Functions and electronic band structures and DOS of model I (left) and model II (right). In partial (b), only Ca and Cl atoms are counted.

To further analyze the partially occupied conduction bands of models I and II in real space, the band decomposed charge densities around Fermi energy are calculated and plotted in Fig. S15a, from which we can find that the charges are distributed mainly around C and Ca. To explore the origin of metallicity, we also calculated the deformation charge density (Δ_1_ρ) and difference charge density (Δ_2_ρ), which are defined as:

Δ_1_ρ = ρ_total_ – ρ_C_ – ρ_Ca_ – ρ_Cl_,

Δ_2_ρ = ρ_total_ – ρ_C_ – ρ_CaCl_

where ρ_total_, ρ_CaCl_, and ρ_M_ are the density of the whole system, CaCl module and M atoms (M = C, Ca and Cl), respectively. Figs. S15b and S15c show that there is significant charge transfer between Ca and Cl. The charge is almost all around the Cl showing strong ionic bond characteristics, and the charges of graphene and Ca are assembled to the position between Ca and graphene, demonstrating significant cation–π interaction between Ca and the aromatic rings in graphene.


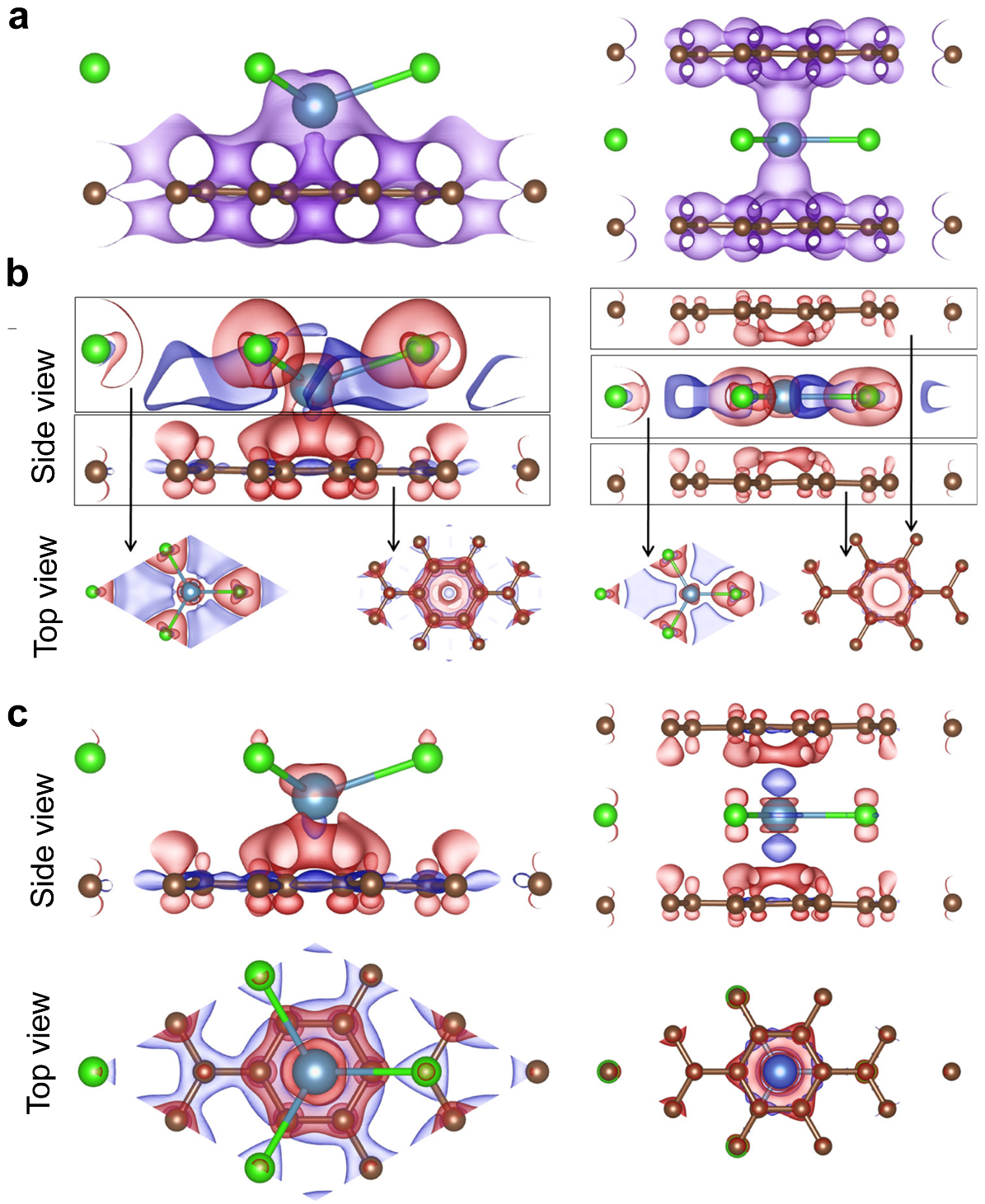


**Fig. S15. Charge analysis of models I and II. a**, Band decomposed charge densities around E_F_. **b** and **c**, deformation charge density (Δ_1_ρ) and difference charge density (Δ_2_ρ) of model I (Left) and model II (Right). In partial (b) and (c), the iso-surfaces of red and blue represent charge accumulation and loss, respectively. Dashed lines in (a) show unit cells. The iso-surface values are 0.0002 e/bohr^3^ in (a) and 0.002 e/bohr^3^ in (b) and (c).

To reveal the electronic properties of such CaCl crystals, the electronic structures of CaCl crystals without graphene in model I were studied. Fig. S16 shows that CaCl alone also presents distinct metallicity because of the dominant contribution of Ca. By comparing the DOS of each component of model I near Fermi energy level, it is interestingly revealed that the metallicity of model I is much stronger than both CaCl alone in model I and single-layer graphene (Fig. S17).


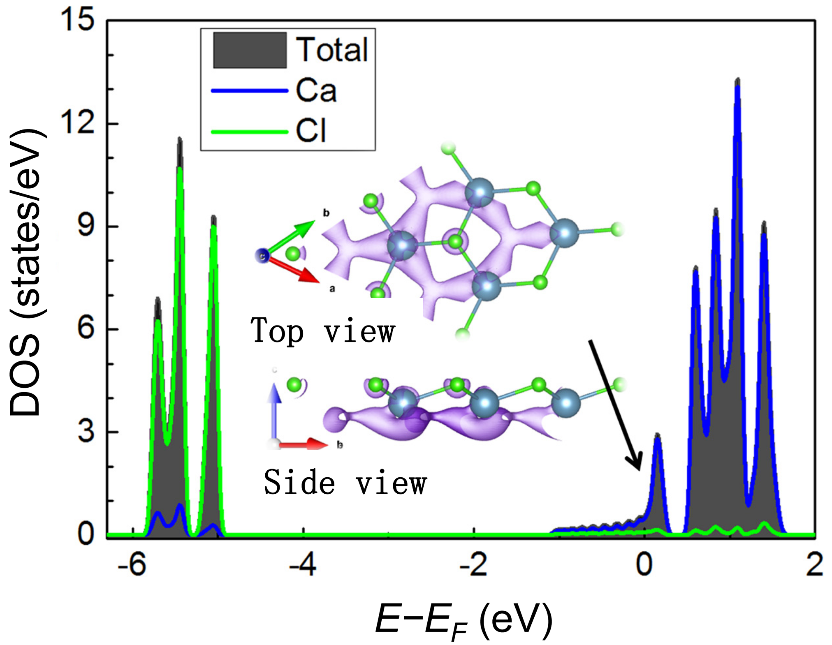


**Fig. S16. Density of states (DOS) of CaCl alone in model I**. Inset: band decomposed charge densities around Fermi energy. The value of iso-surface (purple) is 0.0005 e/bohr^3^.


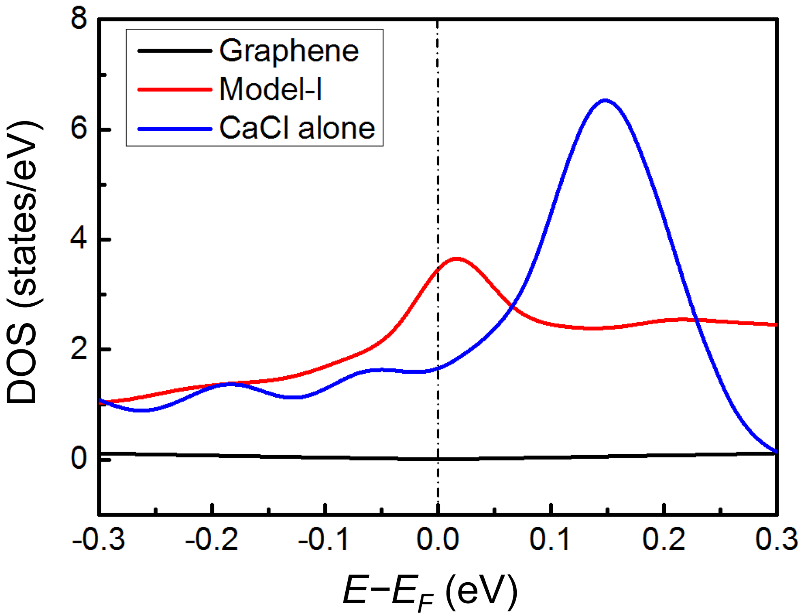


**Fig. S17. DOS of each components in models I around Fermi energy level.** The DOS of CaCl alone in model I, single-layer graphene and model I are represented in blue, black and red.

Further quantitative results of Bader charge analysis of models I and II are listed in Table S3. For both models I and II, Ca transfers ~0.8 (close to 1) electron to Cl, and shares ~0.7 electron with graphene by electron delocalization via cation–π interactions. The Bader charge analysis of CaCl alone in models I and II shows that Ca transfers ~1.0 e^−^ to Cl, shown as Ca^+^, indicating Ca transfer one 4s electron to Cl and share the other one with graphene. In addition, we integrated the DOS of two models from the Dirac point to Fermi energy to approximate the carrier charge, and refer to it as integration charge Δq. For CaCl alone, the lower limit of integral is set to −2 eV (empty band). The results are also shown in Table S3. The Δq equal approximately to the number of Ca atoms, while are almost irrelevant with the quantity or even absence of C atoms. This further confirms our point that the charge involved in conduction is originate from the second 4s valence electron of calcium.

**Table S3.** **The Bader charge ΔQ and integration charge Δq of hexagonal CaCl in model I and II as well as CaCl alone in model I.**

| System | ΔQ of Ca transferred to Cl (e^−^) | ΔQ of Ca shared with C (e^−^) | Δq (e^−^) |
| --- | --- | --- | --- |
| Model I | −0.85 | +0.65 | 1.01 |
| Model II | −0.83 | +0.72 | 0.95 |
| CaCl alone in model I | −1.07 | +1.06 | 0.97 |

To reveal the influence of interlayer interaction, modules containing two layers of model I, three layers of model I, and a 3D structure with periodic boundary along the direction vertical of graphene plane were built and studied. After geometric optimization and AIMD, their energy band structures and DOS are shown in Fig. S18. Similar to models I and II, these three modules also show obvious DOS peaks around Fermi energy, indicating that stacked model I have strong metallicity too.


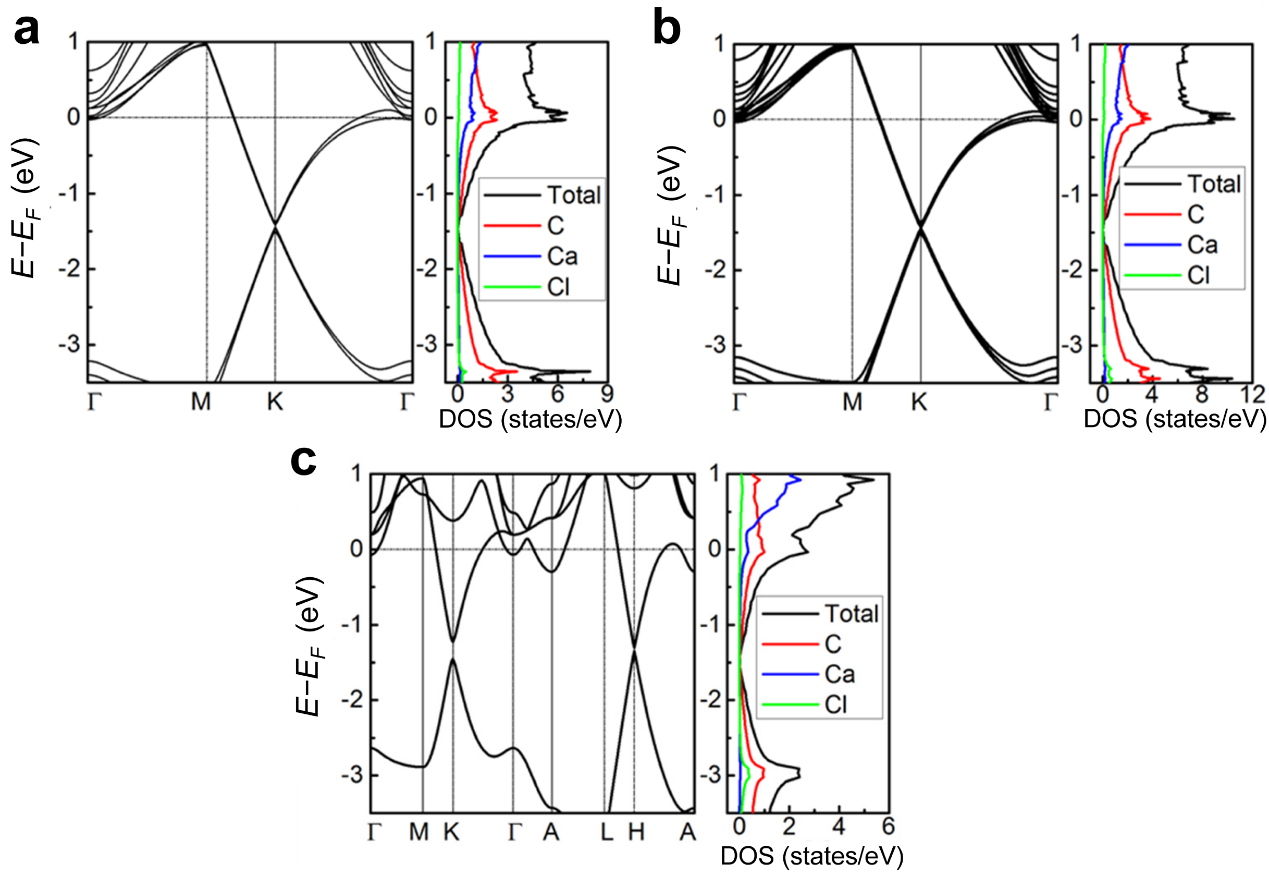


**Fig. S18. Electronic structures of stacked model I.** Energy band structures and DOS of (**a**) two-layer, (**b**) three-layer and (**c**) periodic layers of model I.

To investigate the thermodynamic stabilities and electronic properties of other optimized Ca–Cl crystals of abnormal stoichiometries, we performed further DFT computations for models III–VI (model III, CaCl; model IV, CaCl_4/3_; models V and VI, CaCl_2/3_) which could be formed by a thin slab of conventional CaCl_2_ crystals within a few atomic layers. AIMD simulations show that they have good stabilities (Fig. S19a). The electronic structures clearly show that they have distinct metallicity too (Fig. S19b), similar with other models that have abnormal Ca–Cl atomic ratios.


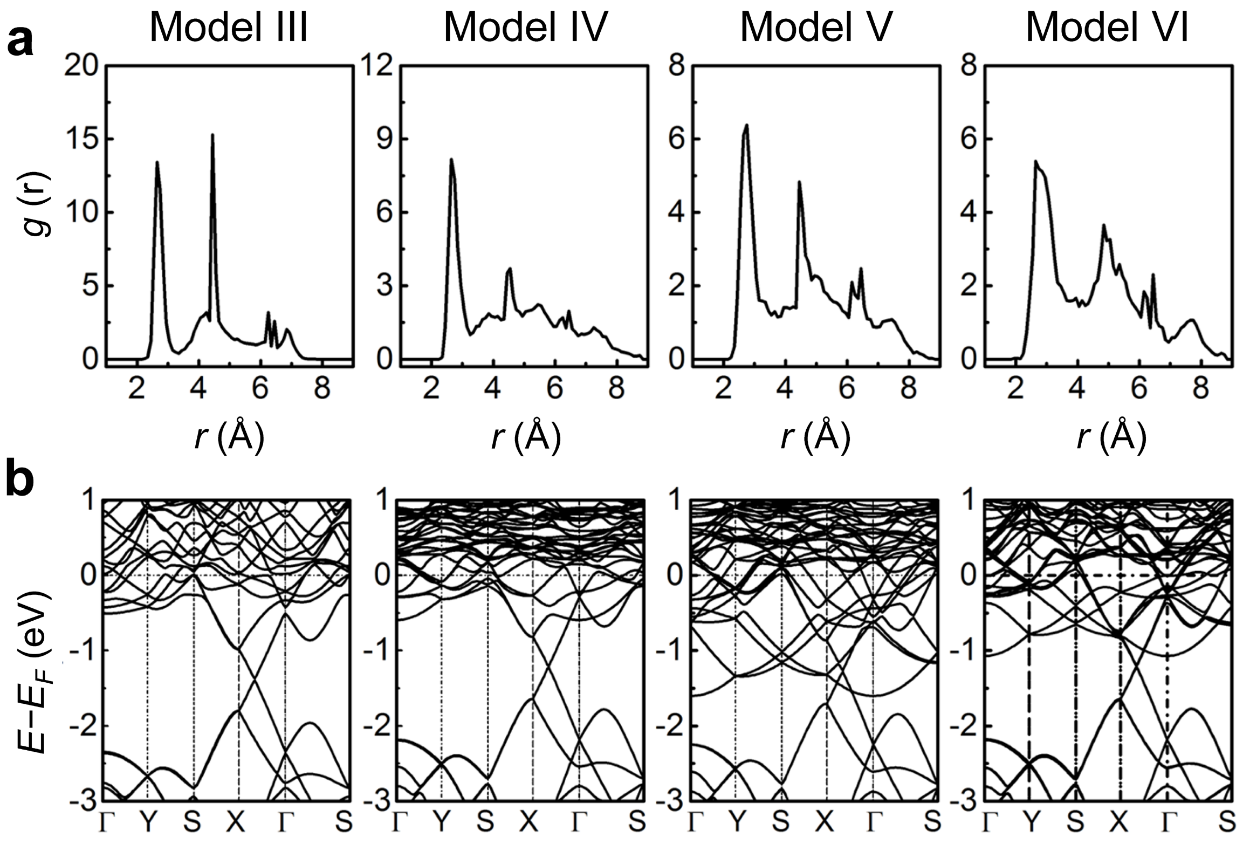


**Fig. S19. Thermodynamic stabilities and electronic structures of models III–VI. a** and **b**, Radial distribution functions and electronic band structures of models III (CaCl), IV (CaCl_4/3_), V (CaCl_2/3_), and VI (CaCl_2/3_). In partial (a), only Ca and Cl atoms are counted, and the functions are statistical average within the whole simulation time (20 ps).

DFT optimized structures (Fig. S11c) also suggest that on a graphene sheet or confined tween two graphene sheets, 2D Ca–Cl crystals with regular stoichiometries (i.e., CaCl_2_) could exist. To further investigate the structural stabilities and electronic properties of such optimized CaCl_2_ modules, we performed theoretical computations for models VII–IX. AIMD simulations show they have good thermodynamic stabilities too (Fig. S20a). However, their electronic structures show small band gaps near Fermi energy levels (Fig. S20b), suggesting such complexes of graphene and CaCl_2_ crystals are not metallic due to the influence of insulating CaCl_2_.


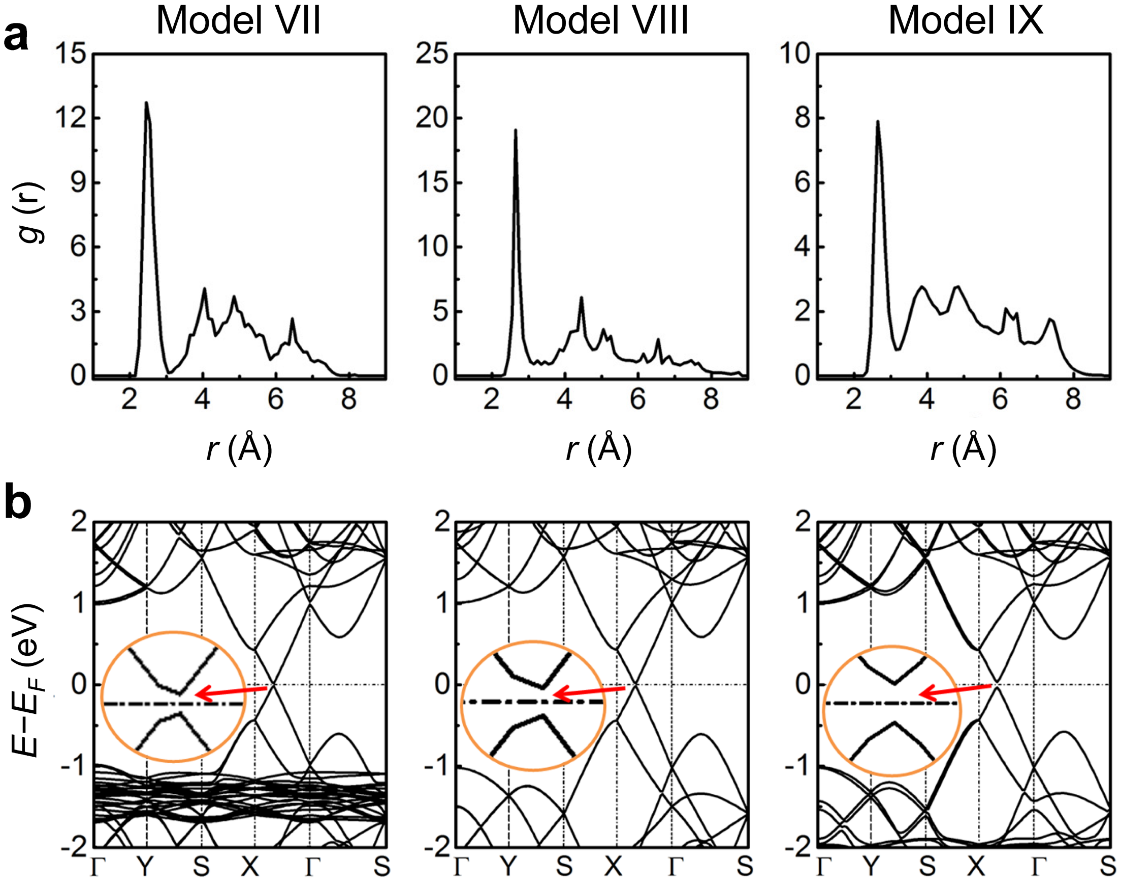


**Fig. S20. Thermodynamic stabilities and electronic structures of models VII–IX. a** and **b**, Radial Distribution Functions and electronic band structure of models VII–IX respectively. In partial (a), only Ca and Cl atoms are counted, and the functions are statistical average within the whole simulation time (20 ps).

**PS4: Valence analysis of the Ca–Cl crystals**

To analyze the valence state of calcium in the dried Ca–Cl@rGO membrane, we have performed NEXAFS (near-edge X-ray absorption fine structure) spectroscopies of pure calcium metal, CaCl_2_ crystals, and Ca–Cl crystals in the dried rGO membranes. Fig. S21 shows that the new L_2,3_-edge peaks of Ca from Ca–Cl crystals in the dried rGO membranes are clearly different from those from the calcium metal with Ca–Ca bonding and the regular CaCl_2_ crystals with Ca^2+^. Importantly, the photon energy of the new L_3_-edge peak of Ca from Ca–Cl@rGO (~348.02 eV) is larger than both those of pure calcium metal (~347.92 eV) and regular CaCl_2_ crystals (~347.82 eV). This shows that this peak from Ca–Cl@rGO cannot be a simple 1:1 combination of the peaks from Ca^0^ and Ca^2+^, and the peak corresponds to a new bonding different from the Ca–Ca bonding. Further, there are clear differences between the relative intensities and energies of the minor peaks in the Ca L-edge NEXAFS spectrum of the Ca–Cl crystals in the rGO membrane (about 346.92 eV and 350.22 eV) and those (about 346.62 eV and 349.92 eV) in the spectrum of calcium metal with Ca–Ca bonding. We note that these minor peaks are related to the symmetry and indirect information about the coordination number of the Ca–Cl system [48]. These differences indicate that the coordination number of Ca in the Ca–Cl crystals in the rGO membrane is different to that of Ca in calcium metal with Ca–Ca bonding.


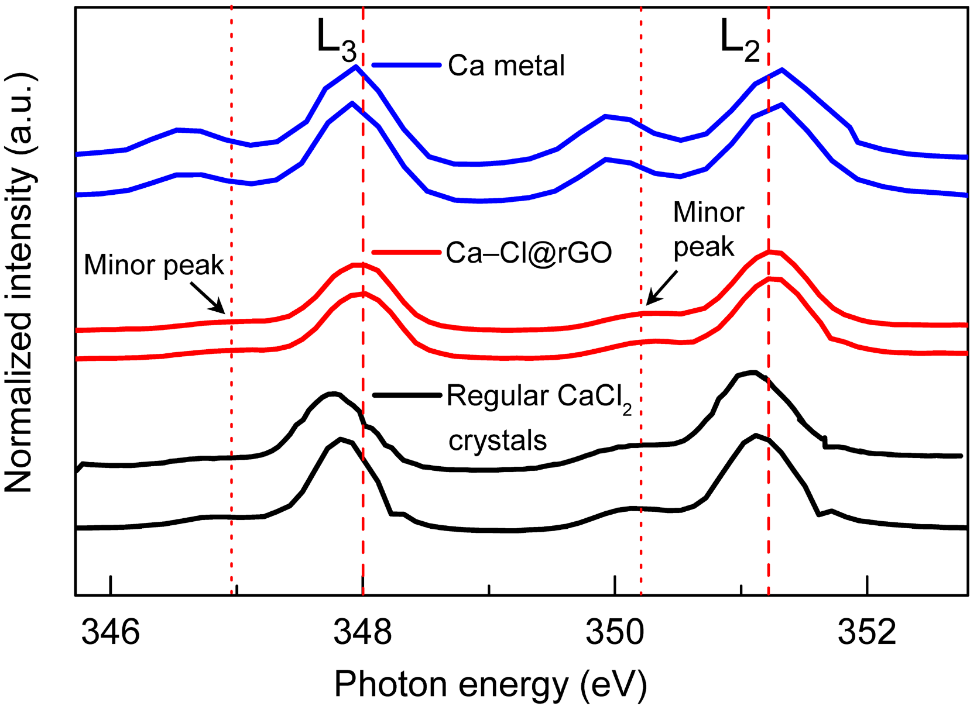


**Fig. S21.** Calcium near-edge X-ray absorption fine structure spectra (NEXAFS) of two sampling areas of the regular calcium metal, the regular CaCl_2_ crystals and the Ca–Cl crystals in rGO membrane.

To further illustrate whether the L_2,3_-edge peaks of the Ca–Cl@rGO in the NEXAFS data are new peaks or the regular divalent Ca^2+^ peaks, we have analyzed the NEXAFS spectra using three Voigt functions (a combination of Gaussian and Lorentzian functions) and a constant peak width for all functions, as shown in Fig. S22b. The raw data, overall fitting, and three Voigt functions are plotted using dots, a red line as well as blue, olive, and magenta lines, respectively, and the residual is marked as the shaded area.

The NEXAFS spectrum of the Ca–Cl@rGO clearly shows the characteristic features of the Ca 2p spin-orbital doublet with an energy splitting at ~3.4 eV (Fig. S22a). Fig. S22b shows that the L_3_-edge peak of the full-width-half-maximum (FWHM) of the main feature (feature 2, ~348.0 eV) for the Ca–Cl@rGO is larger than the FWHM of the regular CaCl_2_ crystals. We note that the increase of the FWHM is mainly attributed to the decrease of lifetime broadening of the electronic states under the same beamline test conditions [49]. This decrease of lifetime indicates that the electronic state becomes less stable and/or the corresponding electrons in the Ca–Cl@rGO become more delocalized (i.e., less tightly bound to nuclei) than the electrons in the regular CaCl_2_ crystals. Furthermore, the spectral intensity of feature 1 (~346.9 eV) for the Ca–Cl@rGO is much smaller than that for Ca metal (Fig. S22b). Thus, these differences in the features (1 and 2) between the Ca–Cl@rGO and regular CaCl_2_ crystal or Ca metal show that the L_3_-edge peak of the Ca–Cl@rGO in the NEXAFS data are neither the peak of the regular divalent Ca^2+^ nor the peak of the Ca metal.

The L_3_-edge peak of the Ca–Cl@rGO in the NEXAFS data is also not a combination of the peaks of Ca^2+^ in the regular CaCl_2_ crystals and Ca^0^ in the metal Ca. The best fitting for the combination ratios of Ca^2+^ and Ca^0^ (i.e., the least standard deviation between Ca–Cl@rGO and the combination) were determined to be 21.5% and 78.5%, respectively (Fig. S22c), using the software fityk 0.98 version [50]. A major deviation remains because of the shifts of the features on the energetic scale. Superposition of the spectra of the CaCl_2_ crystals and Ca metal is not able to yield a good match with the spectra of the Ca–Cl@rGO. In another word, the L_3_-edge peak of the Ca–Cl@rGO in the NEXAFS data is a new peak and does not originate from the peak of the regular divalent Ca^2+^, the peak of the Ca metal, or a combination of these two peaks.


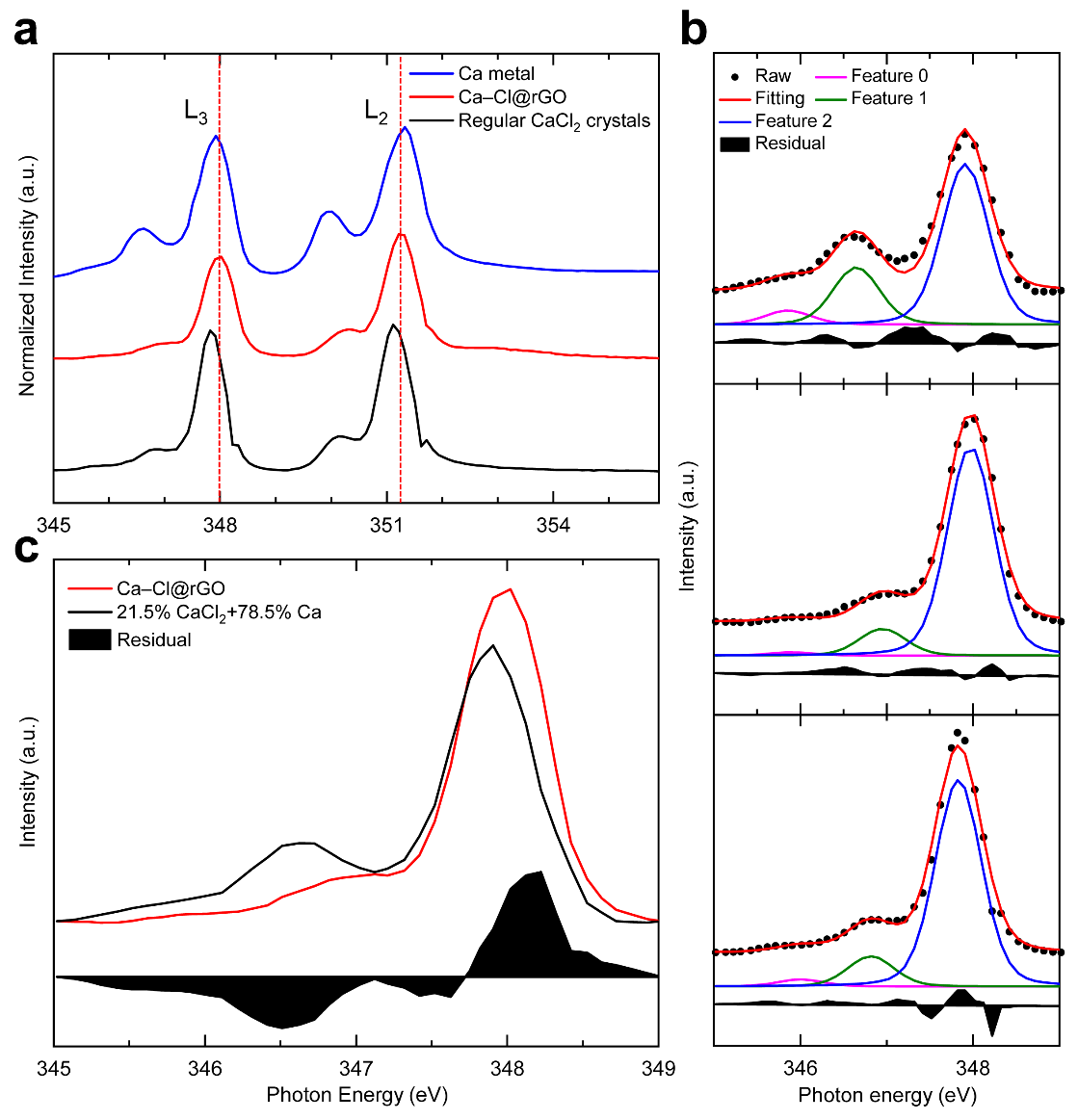


**Fig. S22. Data analyses for Ca metal, regular CaCl_2_ crystals, and Ca–Cl@rGO with three Voigt functions. a**, Calcium NEXAFS spectra of Ca metal, regular CaCl_2_ crystals, and Ca–Cl@rGO. **b**, Data analyses for L_3_-edge features with three Voigt functions. **c**, The best combination of CaCl_2_ and Ca metal (black residual: standard deviation between Ca–I and the combination).

It should be pointed out that we only focused on the fitting of the L_3_-edge features for the following two reasons. i) L_2_-edge features are generally broader and overlap with the tail extended from the L_3_-edge, which makes the quantitative interpretation of the results very complex. ii) As indicated by reference [51], the fitting of the L_2_-edge features does not provide additional information.

Moreover, considering that the valence is the number of electrons participating in chemical bonding, thus we have analyzed the chemical bonding and corresponding electrons participating in the bonding in the Ca–Cl@rGO to illustrate the valence of Ca in the Ca–Cl@rGO. There are two types of interactions of Ca in the Ca–Cl@rGO: i) Ca–Cl interaction and ii) Ca–graphene interaction. Clearly, the ionic bonding is the strong electrostatic interaction between the Ca cation and Cl anion, showing that one electron of the Ca cations participates in the Ca–Cl ionic bonding. Thus, the valence (monovalence or divalence) of Ca in the Ca–Cl@rGO is determined by whether there is a chemical bonding between the Ca and graphene. We note that there are three common types of chemical bonding: covalent, ionic, and metallic bonding. The main interaction between the Ca and graphene is the cation–π interaction. It has been reported in the literature [23] that the cation–π interactions are noncovalent interactions, indicating noncovalent bonding. Difference charge density analyses (Fig. S15) and numerical Bader charge analyses show that the electrons in graphene do not freely delocalize to Ca, and the electrons in Ca do not freely delocalize to graphene either. This behavior indicates that the interaction between the Ca and graphene is not metallic bonding because the electrons in the metal formed by metallic bonding are freely delocalized. The interaction between Ca and rGO is only −42.42 kcal/mol (Table S4), indicating that this interaction is not ionic bonding because the interaction energy (−533.73 kcal/mol) of ionic bonding between Ca and Cl in regular CaCl_2_ is far higher than this value. Furthermore, we have also performed energy decomposition analyses (EDA) to further illustrate the interaction between Ca cations and graphene. Table R1 shows that the interactions between Ca (Ca^+^ and Ca^2+^) and graphene are orbital and electrostatic interactions, which are consistent with the constitution of the cation–π interactions, for example, Na^+^-benzene interaction (Table S4) [52,53]. This finding further confirms that the interaction between the Ca and rGO in the Ca–Cl@rGO is not chemical bonding. In other words, there is only one type of chemical bonding, i.e., the ionic bonding between the Ca cation and Cl anion, indicating that the number of electrons for each Ca in Ca–Cl@rGO participating in chemical bonding is one and thus the Ca is monovalent. This conclusion is further supported by the L_2,3_-edge peaks of Ca from the Ca–Cl crystals in the dried rGO membrane, which are new peaks and distinct from the peaks for the regular CaCl_2_ crystals with Ca^2+^. Therefore, we think that Ca in the rGO membrane has a unique valence state of +1.

**Table S4. Energy decomposition of interactions between Ca^x+^ (x =1, 2) and graphene.**

| Structures | Methods | E (kcal/mol) | Energy decomposition analyses (EDA) | | | |
| --- | --- | --- | --- | --- | --- | --- |
|  |  |  | Orb. | Pauli. | Elst. | Disp. |
| Ca^+^@Graphene | ADF | −42.42 | −60.50 | 74.41 | −51.69 | −4.64 |
| Ca^2+^@Graphene | ADF | −142.88 | −158.36 | 46.76 | −26.65 | −4.64 |
| Na^+^@Benzene | Ref. [52] | −25.71 | −17.92 | 7.57 | −15.37 | / |
|  | Ref. [53] | −31.7 | −19.0 | 11.0 | −14.8 | −8.8 |
|  | ADF | −24.85 | −15.62 | 6.30 | −14.10 | −4.79 |

**PS5: Valence analysis of the Cu–Cl crystals**

For the copper cation, which not only has divalent state Cu^2+^ but also monovalent state Cu^+^, we also performed NEXAFS experiments of the dried Cu–Cl@rGO membrane, regular CuCl_2_ crystals, and regular CuCl crystals, in which the Cu ions are monovalent. The NEXAFS spectra at the Cu L_3,2_ edges are shown in Fig. S23. For Cu in the dried Cu–Cl@rGO membrane, there are Cu L_2,3_-edge peaks for Cu in the dried Cu–Cl@rGO membrane at 925.39 and 945.19 eV (red line), which are consistent with those in the regular CuCl crystal (maroon line), demonstrating the existence of Cu with +1 valence in the Cu–Cl crystals in the dried rGO membrane. We think that this helps to the confirmation of the existence of Ca with a valence state of around +1 in the rGO membrane.


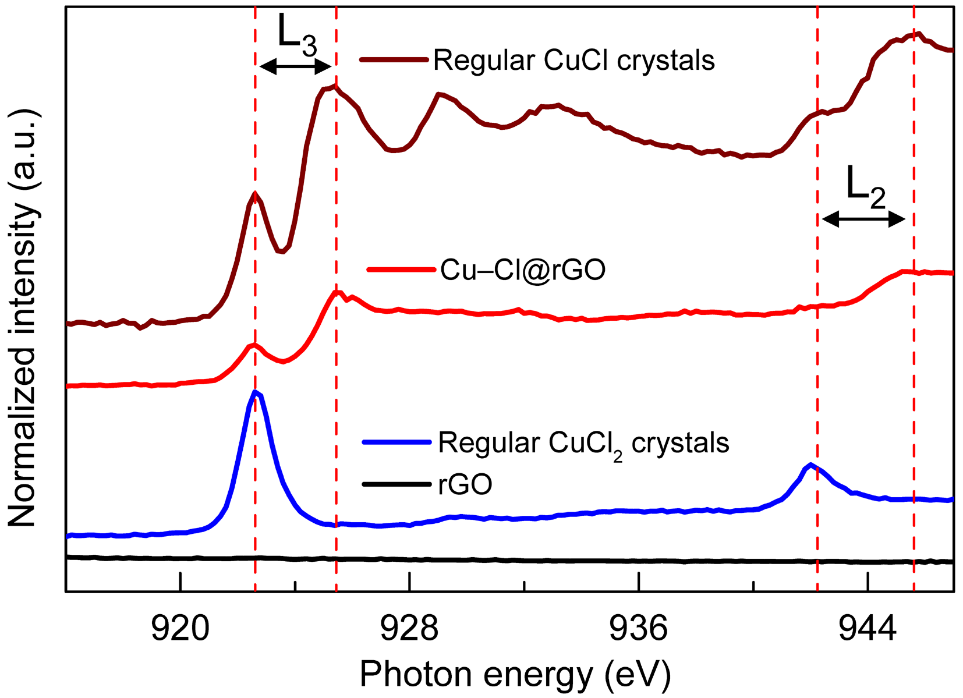


**Fig. S23. NEXAFS spectra of four Cu–Cl compounds at the Cu L_3,2_ edges.** Black: the rGO membrane; blue: regular CuCl_2_ crystals; red: the Cu–Cl crystals in the dried Ca–Cl@rGO membrane; maroon: regular CuCl crystals.

We also used XPS to detect the valence change and further illustrate the stability of Cu^+^ in the dried rGO membranes since it is difficult for us to obtain the enough beam times to perform NEXAFS experiment in the Synchrotron Radiation Facilities (SSRF) for the measurements of XANES near the Cu L edge for tens of days. For the Cu–Cl crystals in dried rGO membranes after incubation with CuCl_2_ solution, the peak of Cu2p_3/2_ at ~932.0 eV (red line and green line: prepared for 10 days and 3days, respectively). Compared with the CuCl (~932.0 eV) and CuCl_2_ (~934.7 eV) crystals, it indicates that there is valence electron change of the Cu ion from Cu^2+^ to Cu^+^ in these crystals, which are consistent with the results of the NEXAFS experiments. Moreover, the peak of Cu^+^ could be clearly observed in the dried rGO membranes after they had been prepared for 3 and 10 days (Fig. S24). Considering that the unstably property of +1 copper ions in the natural surroundings [54] this would help to overcome the difficulty of the application for its unstably in catalyzers based on the +1 copper ions [55].


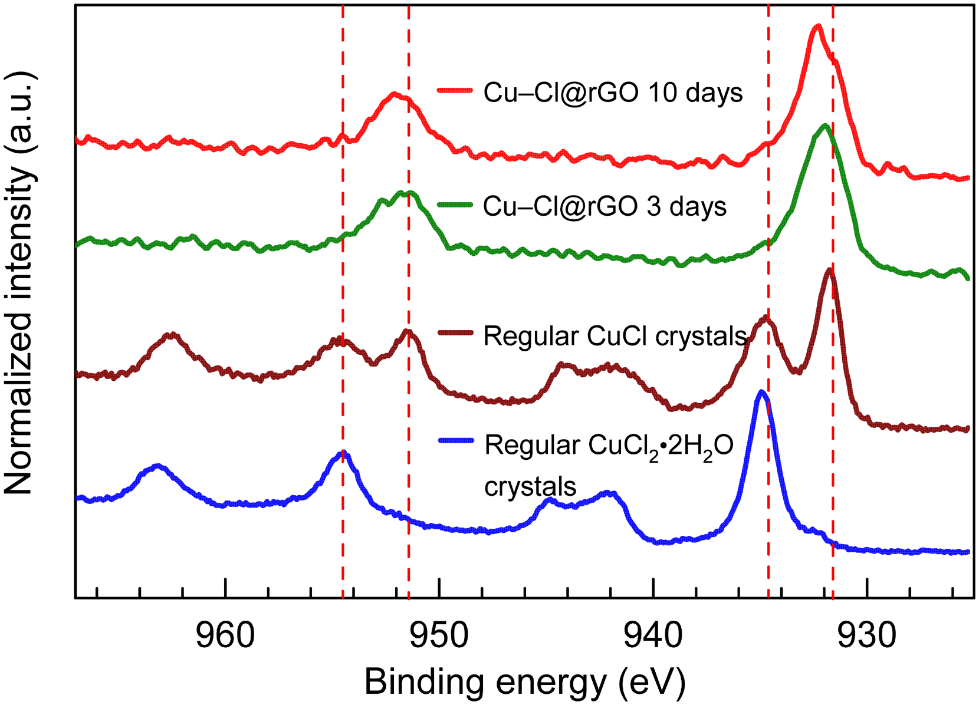


**Fig. S24. X-ray photoelectron spectroscopy (XPS) of Cu–Cl crystals in the dried rGO membranes.** Red and green: the dried rGO membranes with Cu–Cl crystals had been prepared for 10 days and 3 days, respectively. Maroon: regular CuCl crystals; blue: regular CuCl_2_ crystals.

**PS6: Electrical resistivity measurement and calculation**

To confirm the metallic properties of the Ca–Cl crystals in the dried rGO membranes, the electrical resistivity of the whole membrane was experimentally measured.


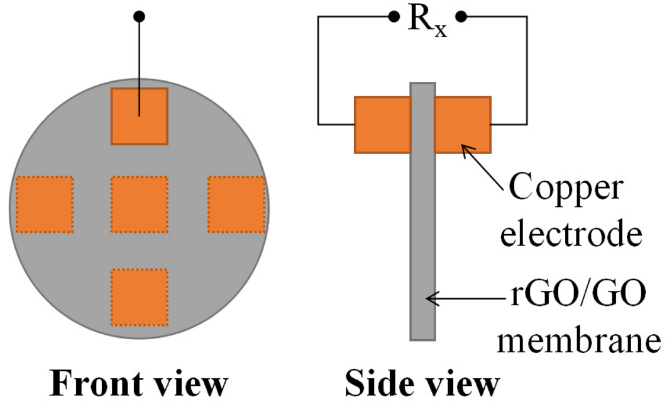


**Fig. S25. Schematic of the experimental setup for electrical resistivity measurement of the dried rGO or GO membranes.** Electrical resistivity of 5 positions measured by applying the multimeter with two copper electrodes connecting on the two side surfaces of the membranes.

The electrical resistivity *ρ* is approximatively calculated as:

*ρ*= *RS* /*L*

where *R* is the electrical resistance, *S* is the contact area of copper electrode, and *L* is thickness of membrane between two copper electrodes.

The average and standard deviation of electrical resistivity were calculated for each membrane and listed in Table S4, it can be seen that the electrical resistivities of dried Ca–Cl@GO membranes are over 10 times less than those of pure dried GO membranes. For dried rGO membranes the electrical resistivities almost keep the same. Considering regular CaCl_2_ crystals are insulating, these results suggest that the Ca–Cl crystals in the dried Ca–Cl@GO and Ca–Cl@rGO membranes are electrically conductive. It should be noted that, since the contact area of the electrodes are less than that of the membranes, the *ρ* calculated here has a certain deviation from the actual value. However, this does not affect our conclusion on the metallic properties of the Ca–Cl crystals.

**Table S5 | Electrical resistivities of rGO and GO membranes**

| Membranes | 5 positions of electric resistivity (MΩ.cm) | | | | | Average  (MΩ·cm) | Std. Dev.  (MΩ·cm) |
| --- | --- | --- | --- | --- | --- | --- | --- |
|  | (1) | (2) | (3) | (4) | (5) |  |  |
| GO-1 | 2.4×10^4^ | 4.1×10^4^ | 5.9×10^4^ | 4.2×10^4^ | 3.0×10^4^ | 4.1×10^4^ | 0.8×10^4^ |
| GO-2 | 3.2×10^4^ | 3.8×10^4^ | 4.3×10^4^ | 4.8×10^4^ | 5.3×10^4^ |  |  |
| GO-3 | 4.6×10^4^ | 3.4×10^4^ | 5.8×10^4^ | 3.9×10^4^ | 3.4×10^4^ |  |  |
| Ca–Cl@GO-1 | 1.5×10^3^ | 1.5×10^3^ | 2.3×10^3^ | 1.1×10^3^ | 9.5×10^2^ | 1.8×10^3^ | 0.6×10^3^ |
| Ca–Cl@GO-2 | 7.0×10^2^ | 1.6×10^3^ | 2.7×10^3^ | 6.1×10^2^ | 1.8×10^3^ |  |  |
| Ca–Cl@GO-3 | 2.3×10^3^ | 3.0×10^3^ | 2.6×10^3^ | 2.5×10^3^ | 1.8×10^3^ |  |  |
| rGO-1 | 9.2×10^−2^ | 1.2×10^−1^ | 6.7×10^−2^ | 5.7×10^−2^ | 8.4×10^−2^ | 3.8×10^−2^ | 3.2×10^−2^ |
| rGO-2 | 6.5×10^−3^ | 4.3×10^−3^ | 2.4×10^−3^ | 2.6×10^−3^ | 3.4×10^−3^ |  |  |
| rGO-3 | 1.7×10^−2^ | 4.6×10^−2^ | 4.4×10^−2^ | 1.1×10^−2^ | 2.1×10^−2^ |  |  |
| Ca–Cl@rGO-1 | 3.1×10^−2^ | 2.4×10^−2^ | 3.1×10^−2^ | 3.7×10^−2^ | 4.2×10^−2^ | 2.4×10^−2^ | 1.5×10^−2^ |
| Ca–Cl@rGO-2 | 4.3×10^−2^ | 2.0×10^−2^ | 4.7×10^−2^ | 3.1×10^−2^ | 4.0×10^−2^ |  |  |
| Ca–Cl@rGO-3 | 2.9×10^−3^ | 1.9×10^−3^ | 1.4×10^−3^ | 1.4×10^−3^ | 1.7×10^−3^ |  |  |

**Conductive AFM** was also used to measure the electrical properties of graphene sheets[56]. The sizes of graphene sheets measured are varied in the range of 4–10 μm.

Figs. S26a and S26b shows the AFM images of the pure graphene sheets and salt solution soaked graphene sheets on the Au coated quartz substrates, respectively. The thicknesses of the graphene sheets covered by the white circles are 2–8 nm and 5–10 nm, respectively. The insets in Figs. S26a and S26b are the current–voltage (I–V) curves of the graphene sheets measured at the red crosses, respectively. It can be seen that the electrical resistance of the pure graphene flake is 486 Ω. And the electrical resistance of the dried graphene sheet with Ca and Cl is below the resistance detection limit of conductive AFM (100 Ω).

**
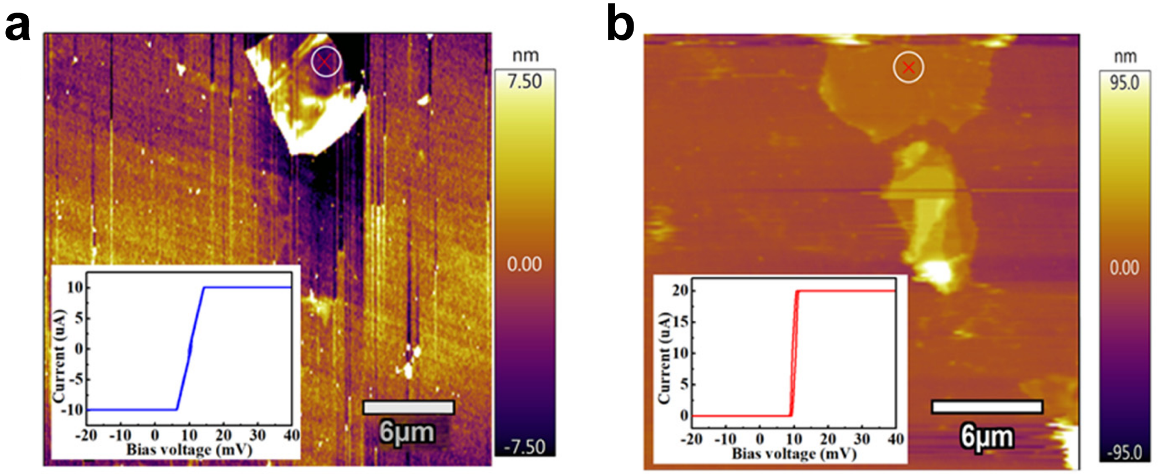
**

**Fig. S26.** AFM images and I–V characteristics of (**a**) the dried pure graphene sheets and (**b**) dried graphene sheets with Ca and Cl on Au coated quartz substrates.

The electrical resistances for the dried pure graphene sheets and dried Ca–Cl@graphene sheets with different thickness are displayed in Fig. S27. The electrical resistances of dried graphene sheets with Ca–Cl crystals are decreased at least 2–6 times over the electrical resistance of the pure graphene sheets with similar thickness, again suggesting the dried Ca–Cl@graphene sheets are metallic.

**
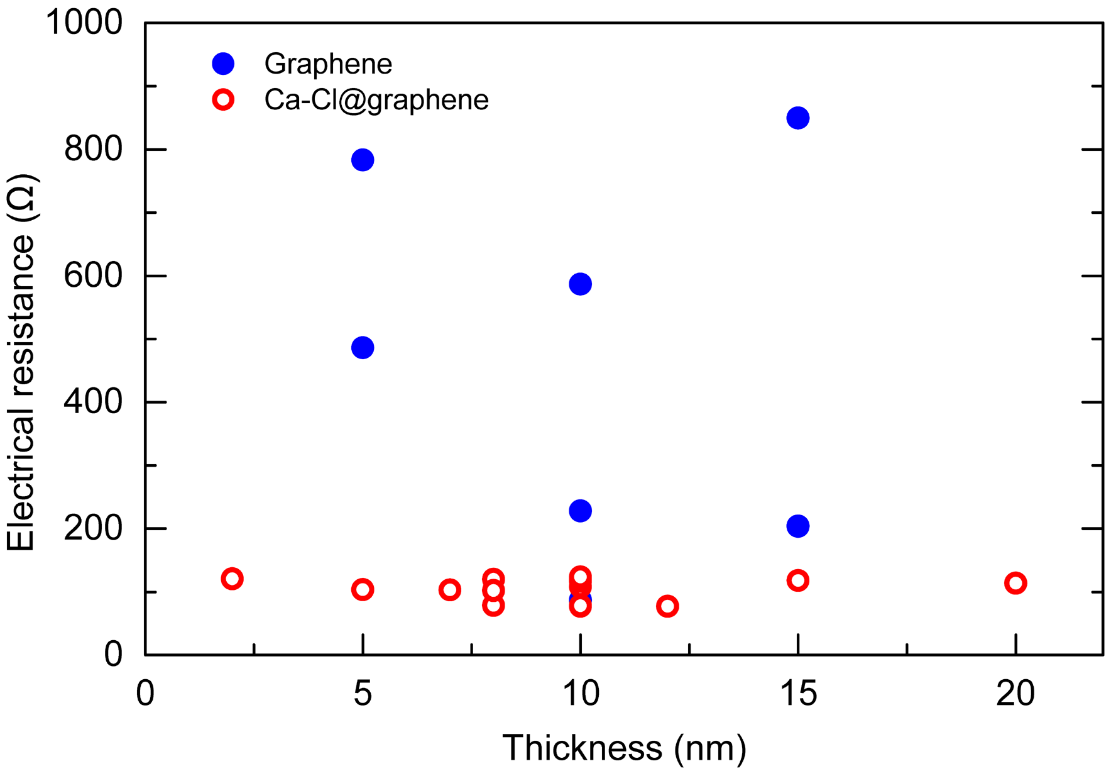
**

**Fig. S27. Electrical resistances of the dried pure graphene sheets (blue dots) and dried Ca–Cl@graphene sheets (red circles) with different thickness.**

To quantitatively evaluate the electrical properties of CaCl crystals, the electrical conductivity of each component of model I as well as model I itself and stacked model I were theoretically calculated. In Table S6, we can find that the electrical conductivity in graphene plane, σ_//_, of model I is doubled relative to double-layer graphene, and the electrical conductivity vertical to graphene plane, σ_⊥_**_,_** also increased in the case of membrane. Despite the uncertainty of stacking mode, here we only present the results of AA stacking as an example. For stacked model I, the in plane electrical conductivity σ_//_ is about 10 times that of AA stacking graphite. It indicates the formation of CaCl crystals could significantly increase the complex’s electrical conductivity compared to pure graphene with similar thickness at nanometer scale.

**Table S6 | Electrical conductivity.** σ_//_ is the electrical conductivity in graphene plane, and σ_⊥_ is the electrical conductivity vertical of graphene plane. The structures of stacked model I are optimized by DFT methods.

| Structure | σ_//_ (Ω^−1^•cm^−1^) | σ_⊥_ (Ω^−1^•cm^−1^) |
| --- | --- | --- |
| Model I | 8.76×10^3^ | / |
| CaCl alone in model I | 2.60×10^3^ | / |
| Double-Layer graphene (AA stacking) | 3.41×10^3^ | 1.01×10**^−^**^2^ |
| Double-Layer model I (AA stacking) | 1.17×10^4^ | 1.15 |
| Periodic graphite (AA stacking) | 9.86×10^3^ | 1.10×10^3^ |
| Periodic model I (AA stacking) | 9.70×10^4^ | 1.14×10^3^ |

**PS7: Graphene–CaCl heterojunction behavior measurement**

To demonstrate the existence of this proposed “graphene–CaCl” junction, we have performed new experiments. The current–voltage curve of the dried Ca–Cl@rGO junction membrane under positive and negative gate voltages is obviously nonlinear and asymmetric, indicating a typical rectification behavior (Fig. S28).

The dried Ca–Cl@rGO membrane samples were prepared by depositing 5.0 M CaCl_2_ solution on the top surface of the membrane under ambient conditions. With the permeation of the solution, metallic Ca–Cl crystals were formed in the inner sheets with vertically decreased concentrations, finally forming a *metallic CaCl–graphene* (top to bottom) heterojunction configuration in the membrane after drying.


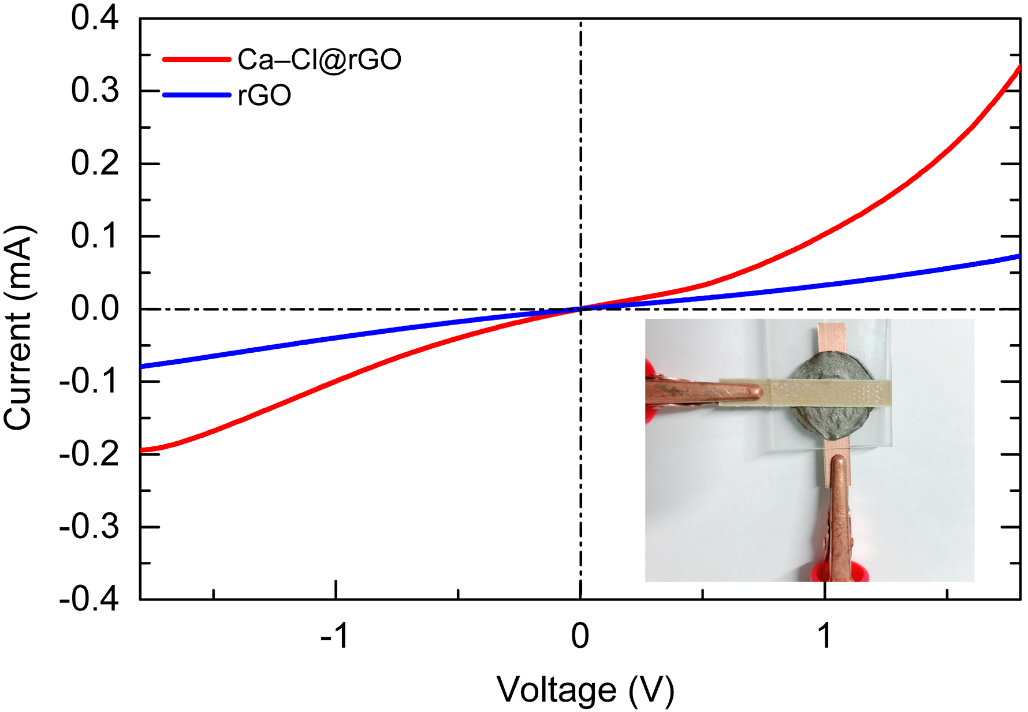


**Fig. S28. Current–voltage curves for a dried Ca–Cl@rGO membrane (red) and a dried rGO membrane (blue).** The inset shows the photo of the dried membrane connected by two Cu foil electrodes for current–voltage measurement.

**PS8: Piezoelectricity-like property calculation and measurement**

Because the 2D metallic Ca–Cl crystals have two elements (Ca and Cl) that are alternately distributed, we speculate that the material containing these Ca–Cl crystals should show piezoelectricity since the electric effects of the two different elements are different under compressive or tensile strain. DFT calculations show that the charge distribution is different after tensile deformation. For 5% geometric elongation along the *x* direction, the charge distributions of all the Ca, Cl, and C atoms in model I show significant relocation (Fig. S29), indicating that the dried Ca–Cl@rGO membrane shows a relatively strong piezoelectricity.

**
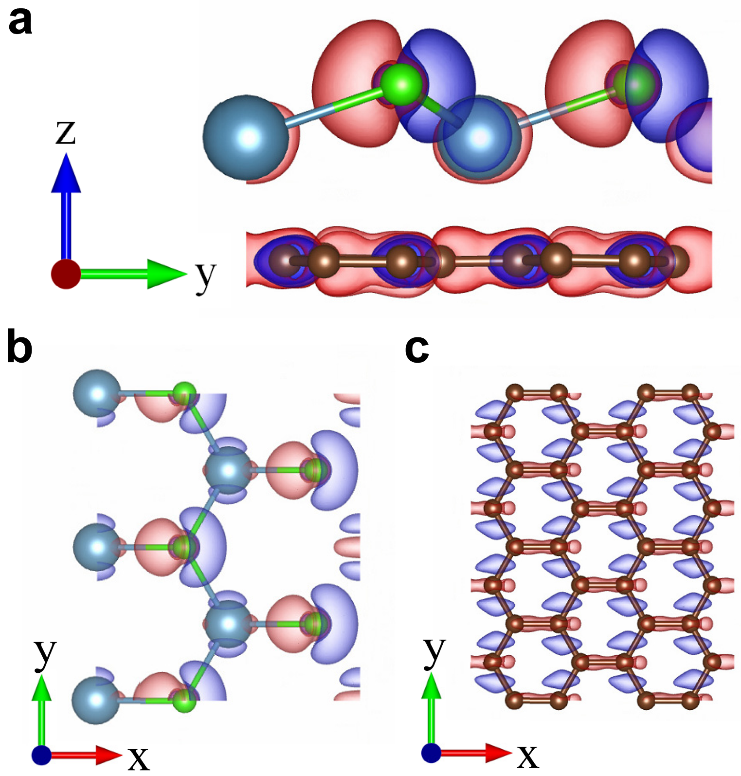
**

**Fig. S29. Difference in the charge distributions of the Ca, Cl, and C elements after recentering the position of each atom in model I under 5% structural elongation along the *x* direction. a**, Side view and **b** top view of the CaCl layer. **c**, Graphene layer. The blue and red isosurfaces show charge gain and loss, respectively.

Further analyses of the anisotropic Ca–Cl@rGO structure have revealed the coexistence of conductive charges confined in a narrow layer parallelly attached to the graphene plane and the electric dipoles formed by Ca and Cl in the normal directions of the graphene plane, and the latter induce the piezoelectricity in the Ca–Cl@rGO. The electronic structure at the Fermi level is mainly derived from orbital states, which are decoupled from the ions undergoing polar displacements so that there is no conflict between the properties of metallicity and piezoelectricity in our system.

The conductive charge in model I is mainly distributed in the narrow layer around the graphene plane, as indicated in the DFT calculation described in our previous response. The electrical conductivity is mainly contributed by the π-electron of graphene and the one 4*s* electron of each Ca. As shown in Fig. S30, the charge around the Fermi level distributes on both sides of the graphene plane and is almost independent of the energy range from 0.1 to 4.0 eV below the Fermi level. The distribution is more delocalized in the plane parallel to graphene rather than in the vertical direction (*z* direction) and at a distance from Ca^+^ and Cl^−^ ions, indicating that the conductive electrons are located and conduct within the narrow layer around the graphene plane and thus have a limited effect on screening the polarizations of Ca^+^–Cl^−^ dipoles along the *z*-direction.


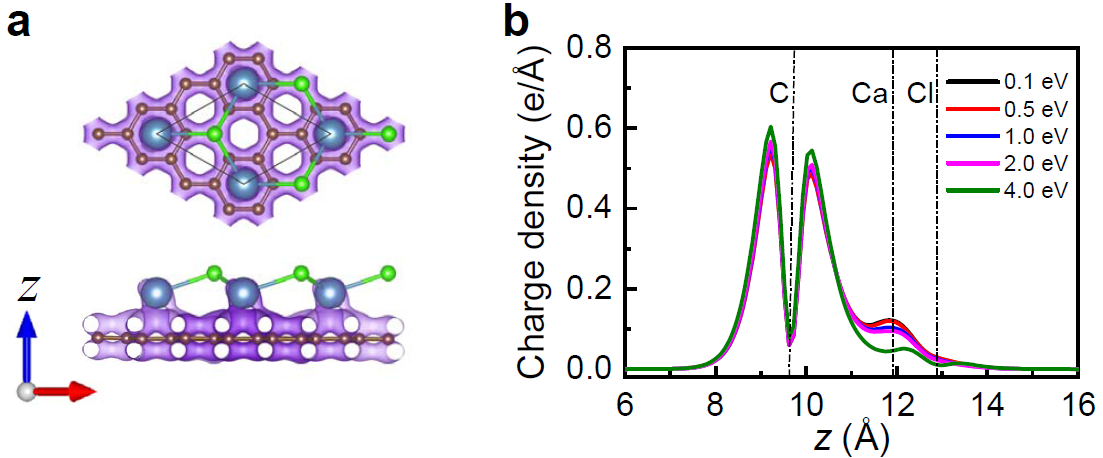


**Fig. S30. Conductive charge distribution in the system with model I configuration. a**, Distribution of charge between *E_F_* −1.0 eV to *E_F_*. The Ca, Cl, and C atoms are shown in blue, green and brown, respectively. **b**, Conductive electron (around Fermi level) along the *z*-direction. The solid lines of different colors represent different energy ranges below the Fermi level, and the dashed lines denote the atomic positions in the *z*-direction.

The electric dipoles are mainly derived from the CaCl structure, which can be approximated as Ca^+^ and Cl^−^ ion pairs (one *4s* electron of Ca transfers to Cl). The polarization in the system Ca–Cl@rGO mainly originates from the dipoles since there is a height difference between Ca^+^ and Cl^−^ in the *z*-direction. This results in a net electrical dipole moment along the *z*-direction, which induces piezoelectricity.

Furthermore, we estimated the value of the piezoelectricity in model I. To simulate the strain perpendicular to the graphene plane induced by external stress during bending process, we moved the Cl along the *z*-direction from its original equilibrium position. The relative strain Δ*S* can be defined as

Δ*S* = $\frac{\Delta Z_{\mathrm{Cl}}}{h}$, (1.1)

where Δ*Z*_Cl_ is the displacement of Cl in *z*-direction from its original equilibrium position, and *h* is the thickness of the graphene with Ca and Cl (*h* can be calculated as the distance from Cl to graphene since Cl is on the top of the carbon atoms in graphene (Fig. 31a)). The piezoelectric polarizability *D* can be calculated by

$D=\frac{\Delta P}{\Delta S}$, (1.2)

where $\Delta P$ is the corresponding change of the electrical dipole moment density under Δ*S* within the volume of the unit cell of model I. $\Delta P$ mainly includes three parts, $P_{\mathrm{Ca}}$, $P_{\mathrm{Cl}}$, and $P_{e}$, corresponding to the changes of dipole moment density contributed from Ca^+^, Cl^–^, and conductive electrons, respectively. Explicitly, $\Delta P$ can be calculated by

${P=P_{\mathrm{Ca}}+P}_{\mathrm{Cl}}+P_{e}$, (1.3)

where $P_{\mathrm{Cl}}$ is determined as

$P_{\mathrm{Cl}}=\frac{Q_{\mathrm{Cl}}Z_{\mathrm{Cl}}}{V}$, (1.4)

in which *Q*_Cl_ is the ionic charge of Cl^–^, and *V* is the volume of the unit cell.

$P_{\mathrm{Ca}}$ is determined as

$P_{\mathrm{Ca}}=\frac{Q_{\mathrm{Ca}}Z_{\mathrm{Ca}}}{V}$, (1.5)

where *Q*_Ca_ is the ionic charge of Ca^+^.

DFT computation shows that the *z* coordinate of Ca is linearly changed with the displacement of Cl (Fig. S31b). The slope, *k*, of the displacements between Cl and Ca is ~0.147.


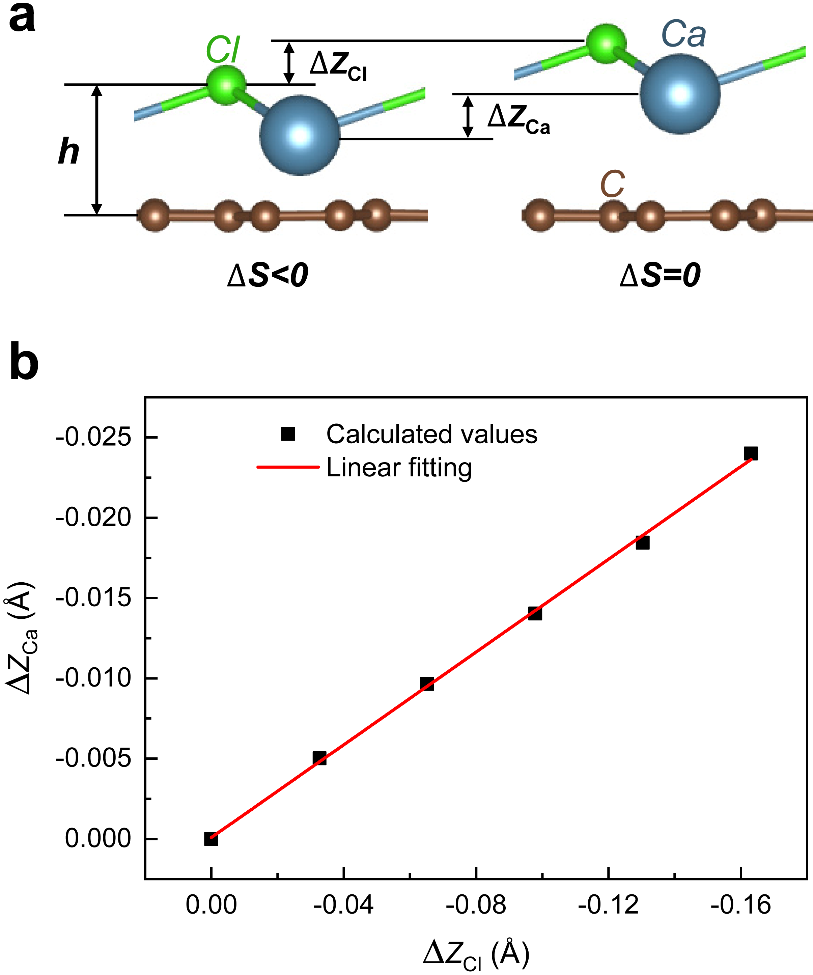


**Fig. S31. Displacement of Ca and Cl with different strains.** **a**, Illustration of the strain in model I perpendicular to the graphene plane induced by external stress during bending process. Δ*Z*_Ca_ and Δ*Z*_Cl_ are the displacements of Ca and Cl in *z*-direction from their original equilibrium positions, respectively; *h* is the thickness of model I calculated as the distance from Cl to the graphene plane. **b**, Δ*Z*_Ca_ versus corresponding applied Δ*Z*_Cl_. The black squares are the computation results. The red line is the linear fitting.

Therefore, $P_{\mathrm{Ca}}$ can be calculated as

$P_{\mathrm{Ca}}=\frac{{kQ}_{\mathrm{Ca}}Z_{\mathrm{Cl}}}{V}$. (1.6)

$P_{e}$ is determined from the integration of conductive electrons as

$P_{e}=\frac{1}{V}\int(\rho-\rho_{0})zdxdydz$, (1.7)

where *ρ* and *ρ*_0_ are the densities of conductive electrons with and without relative strains, respectively.

Substituting Eqs. (1.4), (1.6) and (1.7) in (1.3), we obtain

$P=\frac{{(kQ}_{\mathrm{Ca}}+Q_{\mathrm{Cl}})Z_{\mathrm{Cl}}}{V}+\frac{1}{V}\int(\rho-\rho_{0})zdxdydz$. (1.8)

$h$, $Q_{\mathrm{Ca}}$, $Q_{\mathrm{Cl}}$, *V*, *ρ* and *ρ*_0_ were numerically calculated using DFT computation for a given value of $Z_{\mathrm{Cl}}$. $S$ and $P$ were correspondingly determined based on Eqs. (1.1) and (1.8), with the results as shown in Fig. S32. According to Eq. (1.2), we obtained the best fitting for the piezoelectric polarizability *D*, which is 0.030 e^–^/Å^2^ (i.e., 0.480 C/m^2^). We note that this calculated value is comparable to the calculated values of piezoelectric polarizability of boron-nitride and monolayer MoS_2_ of ~0.163 C/m^2^ and ~0.646 C/m^2^, respectively [57].


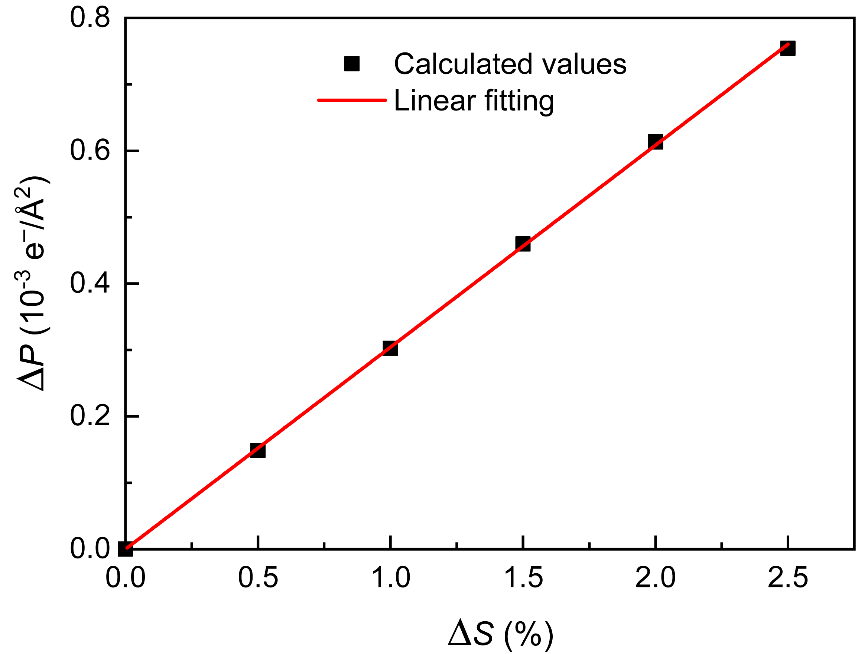


**Fig. S32. Change of the electric dipole moment Δ*P* versus relative strain Δ*S*.** The black squares are the computation results. The red line is the linear fitting.

In a word, the conductive electrons and electric dipoles coexist but are spatially separated, which is the origin of the piezoelectricity in the Ca–Cl@rGO membranes.

To verify the above theoretical prediction, we have prepared the dried Ca–Cl@rGO membranes by first incubating the GO membranes in 5.0 M CaCl_2_ solutions overnight and then centrifuging to remove the free solution and drying at 180 °C for more than 4 hours. The dried Ca–Cl@rGO or dried rGO membranes were then connected by two Cu foil electrodes and fixed by two flexible plastic plates (Fig. S33a).The voltages between the top and bottom of the membranes was measured when the two plastic plates were bent to a certain angle (Fig. S33b). Fig. S33c shows the piezoelectric-like open-circuit voltages of the dried Ca–Cl@rGO and rGO membranes with a bending angle of 90^°^. The maximum output voltage is ~4 mV when the membrane is bent, and the output voltage decreases, reaches zero and may even overshoot to negative values when we remove the bending force from the membrane. In contrast, we cannot detect a visible output voltage for the dried rGO membrane with the same bending angles. The output voltages of the dried Ca–Cl@rGO and rGO membranes as a function of the bending angle are shown in Fig. S33d. The output voltage of the dried Ca–Cl@rGO membrane increases with increasing bending degree, whereas the output voltage of the dried rGO membrane is always around 0 mV for various bending angles up to 180°.

The existence of the piezoelectricity-like property for the metallic CaCl is unexpected, because metallic materials generally have no piezoelectricity, as the sentences in the literature [58]: “piezoelectricity is only possible in materials which have non-centrosymmetric structures and a bandgap, i.e., are non-metallic” and “existing 2D materials are piezoelectric if and only if they fulfill two criteria: they must have a non-symmetric crystal structure and they must be non-metallic (i.e., have a band gap)”. Such unexpected behavior is induced by the abnormal 2D CaCl structure that the structure is metallic due to the monovalent behavior of the Ca ions on the one hand, and on the other hand the structure has two elements (Ca and Cl) with different electric effects under compressive or tensile strain. Thus, the 2D Ca–Cl crystals are a completely novel material that has both metallic character and piezoelectric-like property.


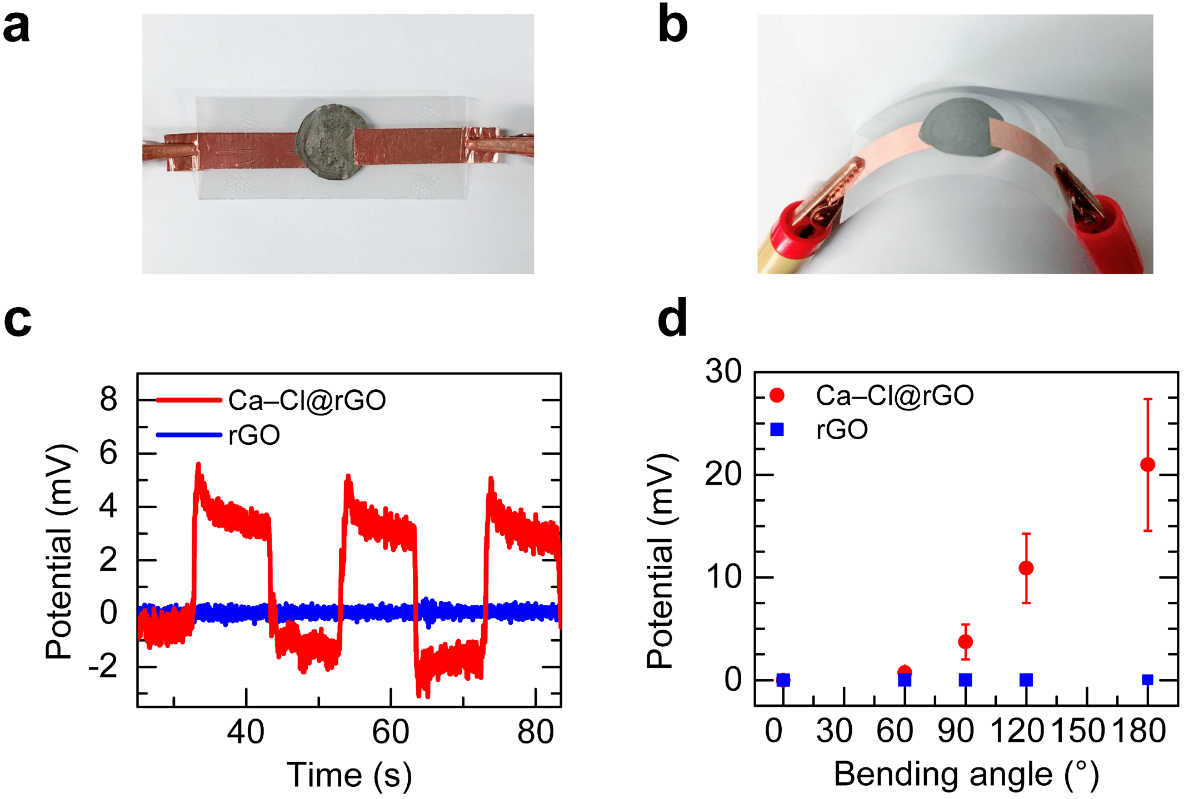


**Fig. S33. Piezoelectricity-like property of the dried Ca–Cl@rGO membrane. a**, Photo of the system in piezoelectricity-like property measurement. A dried Ca–Cl@rGO or a dried rGO membrane is connected by two Cu foil electrodes fixed by flexible plastic plates. **b**, Photo of the system in piezoelectricity-like property measurement of the dried membrane bent to a certain angle **c**, Typical voltage responses from a dried Ca–Cl@rGO membrane and a rGO membrane under periodic strain as a function of time for bending angles up to 90°. **d**, Piezoelectric-like property outputs from a dried Ca–Cl@rGO membrane (red dots) and a dried rGO membrane (blue squares). The mean values and standard deviations of the output potentials were calculated from ten technical replicates.

**PS9: Room-temperature ferromagnetism measurement and calculation**

The ferromagnetism of the dried Ca–Cl@rGO membrane was measured by using a quantum design MPMS-SQUID VSM-094 magnetometer. Fig. S34a shows that the saturation magnetic moment (*M*_s_) of the dried Ca–Cl@rGO membrane is ~2.1 emu/g at 300 K, whereas *M*_s_ = ~0.4 emu/g for the dried rGO membrane at the same temperature. Considering the mass percentage of calcium is only ~4.7% (Fig. S9), the existence of CaCl crystals greatly enhances the ferromagnetism of the membrane with an averaged saturation magnetic moment of ~0.24 μ_B_ per calcium atom. For other metals, such as Cu, room-temperature ferromagnetism have also been experimentally observed in the dried Cu–Cl@rGO membrane (Fig. S34b), in which there are also monovalent Cu^+^ ions, prepared by incubating a rGO membrane in CuCl_2_ solution under ambient conditions.

**
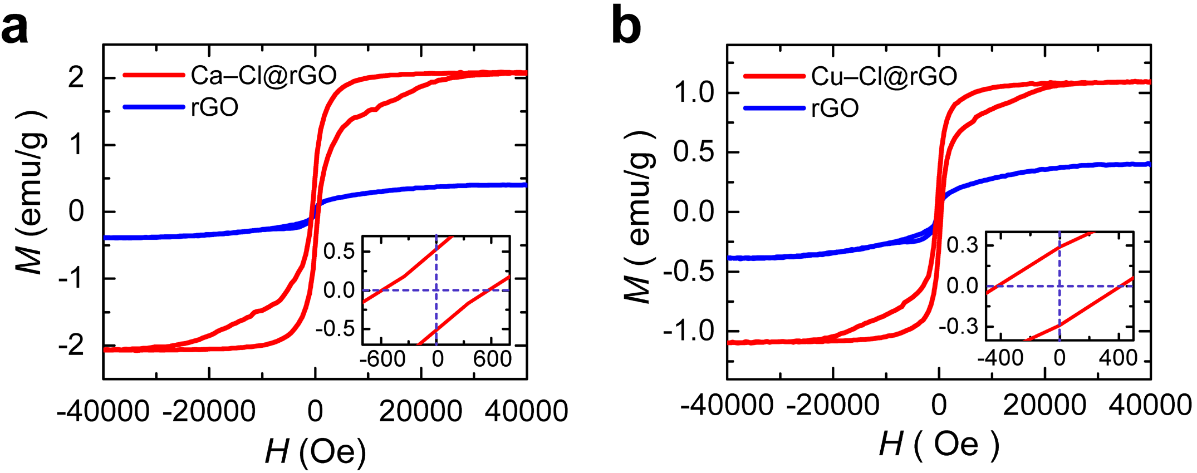
**

**Fig. S34. Magnetic moment *M* versus applied magnetic field *H* of the dried rGO, Ca–Cl@rGO, and Cu–Cl@rGO membranes at 300 K after subtraction of the linear diamagnetic background.** The inset shows a low-field zoom of the curves from the main panel where excess moments and coercive force are seen clearly. The magnetic field is set to be perpendicular to the sample surface.

To discuss the possible origin of such strong ferromagnetism in the dried Ca–Cl@rGO membrans, we performed spin polarized calculation and found no net spin polarization in a system with periodic model I configuration. As shown in Fig. S35, the spin-resolved band structure shows that the electron energy bands of spin up and down are completely coincident, indicating that there is no ferromagnetism in a system with periodic model I configuration (Figs. S35a and S35b). Furthermore, the difference in the spin-polarized density of states (Fig. S35c) suggests that the net magnetic moment *M* of the system is clearly zero. However, the energy shift $\Delta E$ between the peaks of the spin-resolved density of states is zero from the DFT computation. This result implies that the Stoner exchange parameter (*I_s_*$=\Delta E/M$) is close to zero, which is too small to induce ferromagnetism because the electron states around the Fermi level are generally of *s* or *p* character. In view of the Stoner criterion, the Stoner parameter *I_s_* ~0 eV and the state density *N*(*E_F_*) in this case is approximately 4 states/eV (Fig. 2 in the main text), which is the same as the state density of the non-spin polarized case, resulting in

*N*(*E_F_*) *I_s_* << 1.

Therefore, the Stoner criterion, i.e., *N*(*E_F_*) *I_s_* > 1, is not satisfied, implying that the origin of ferromagnetism is not Stoner's mechanism.


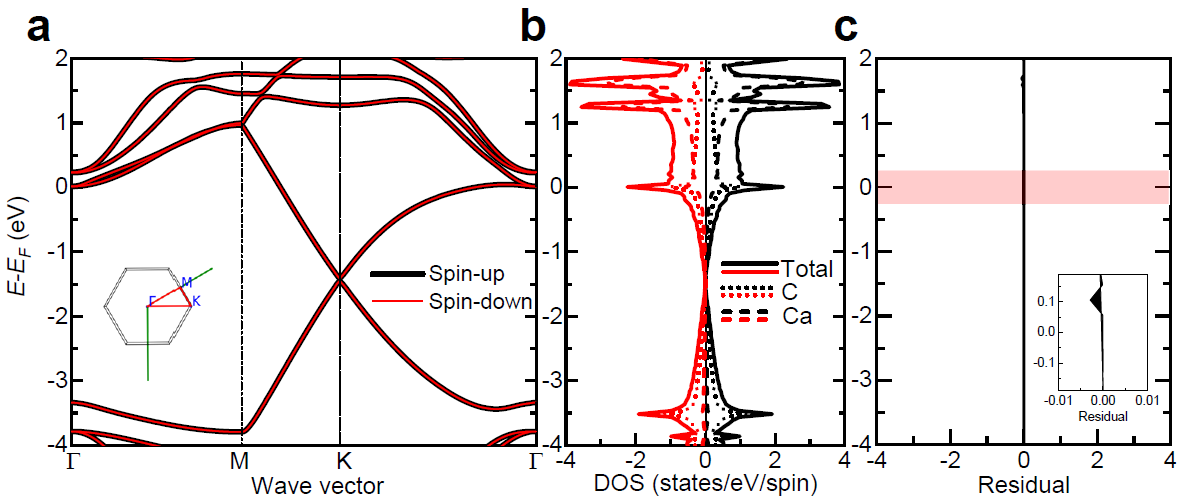


**Fig. S35. Spin-resolved band structures (a), spin-polarized density of states of model I (b), and the difference of spin-polarized density of states (c).**

The DFT calculations on antiferromagnetism configuration of Ca–Cl@rGO were also performed. We constructed 2×2×1 and 1×2×1 supercells of model I to investigate the antiferromagnetism by assigning opposite initial magnetic moments in adjacent unit cells (Fig. S36), respectively. After DFT optimization, the energy band was also calculated, in which there remained no spin split in the 2×2×1 and 1×2×1 systems. This finding suggests that there is no antiferromagnetism in a system with the periodic model I configuration.


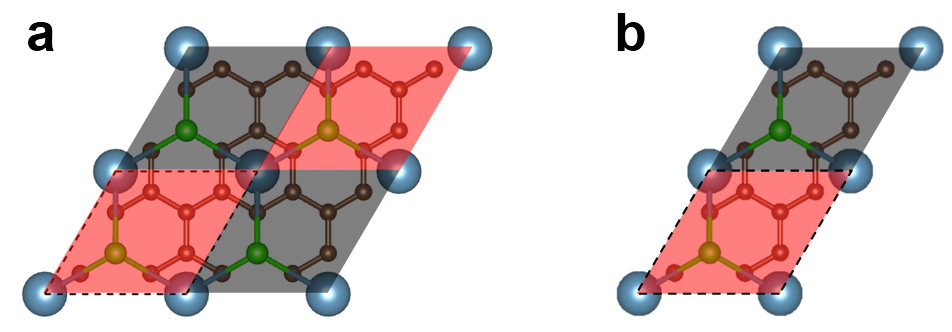


**Fig. S36. Spin-resolved band structures of supercells of model I.** (**a**) and (**b**) Structures of 2×2×1 and 1×2×1 supercell; opposite initial magnetic moments are located in black (spin-up) and red (spin-down) translucent regions.

Considering the finite sizes of the Ca–Cl crystals in the dried Ca–Cl@rGO membrane owing to the complex morphology features in the rGO membrane (Figs. S1–S3, and S7), the edge effect may cause magnetism [59,60]. Therefore, we performed further theoretical calculations of a CaCl cluster with model I configuration and adsorbed on a periodic graphene substrate. As shown in Fig. S37 and Table S7, such clusters indeed show relatively strong magnetism with an averaged magnetic moment per Ca ranging from 0.1553 μ_B_ to 0.7677 μ_B_. In order to obtain insight into the magnetic origin, we further calculated the spin density of a unit cell containing two CaCl molecules with different distances. As shown in Fig. S38, the spin of CaCl molecules are parallel to each other with a total magnetic moment of 1.9984 μ_B_ and 0.9984 μ_B_ with two different distances, both showing strong spin polarization around Ca and therefore ferromagnetic characters. For the case of periodic clusters containing one and two CaCl molecules along one dimension (Fig. S39), the spin of CaCl molecules are also parallel after DFT optimization, showing ferromagnetism.

In the case of point defect which is also considered as a major origin of magnetism [61,62], we calculated the spin density of a unit cell with model I configuration and Ca, Cl, and CaCl defects (Fig. S40a). After DFT optimization, it is found that the unit cell containing only one Ca defect or one Cl defect is nonmagnetic, while defects of one CaCl or two CaCl molecules can induce magnetism (Table S8). In the case of the one CaCl defect, the total magnetic moment is ~0.8972 μ_B_ (Table S8) and the spin density is majorly localized around Ca with suspended bonds (Fig. S40b), indicating that the magnetism is induced mainly by Ca. While in the case of two CaCl defects, the total magnetic moment of the system is doubled (~1.9989 μ_B_) with parallel spin distribution majorly around Ca (Fig. S40c and Table S8), indicating the system is ferromagnetic.


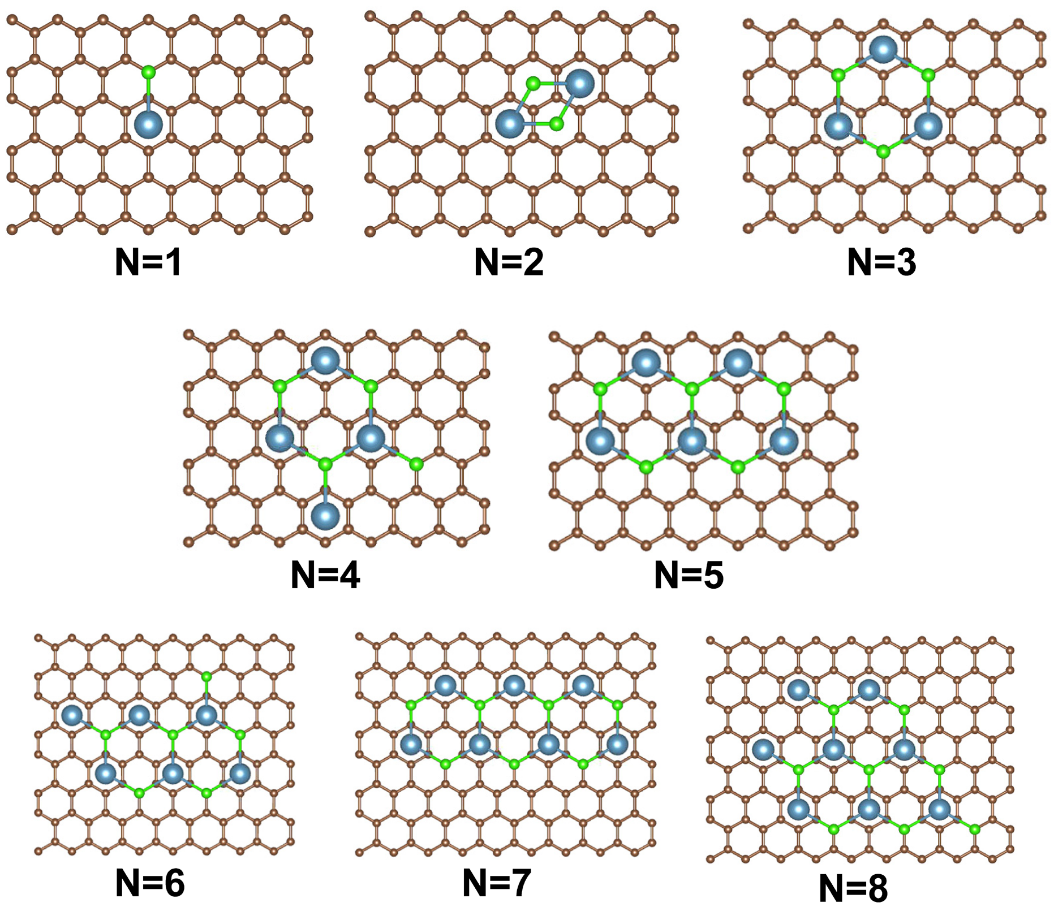


**Fig. S37. Configurations of (CaCl)_N_ clusters with N-pairs of CaCl molecules having the structure as model I on graphene substrate.**

**Table S7. Magnetic moments of (CaCl)_N_ clusters with the configurations as shown in Fig. S36.**

|  | CaCl | (CaCl)_2_ | (CaCl)_3_ | (CaCl)_4_ | (CaCl)_5_ | (CaCl)_6_ | (CaCl)_7_ | (CaCl)_8_ |
| --- | --- | --- | --- | --- | --- | --- | --- | --- |
| Total moment *M* (μ_B_) | 0.7677 | 1.4706 | 0.4659 | 1.4136 | 0.8665 | 0.9768 | 1.5687 | 1.3728 |
| *M*/N (μ_B_) | 0.7677 | 0.7353 | 0.1553 | 0.3534 | 0.1733 | 0.1628 | 0.2241 | 0.1716 |


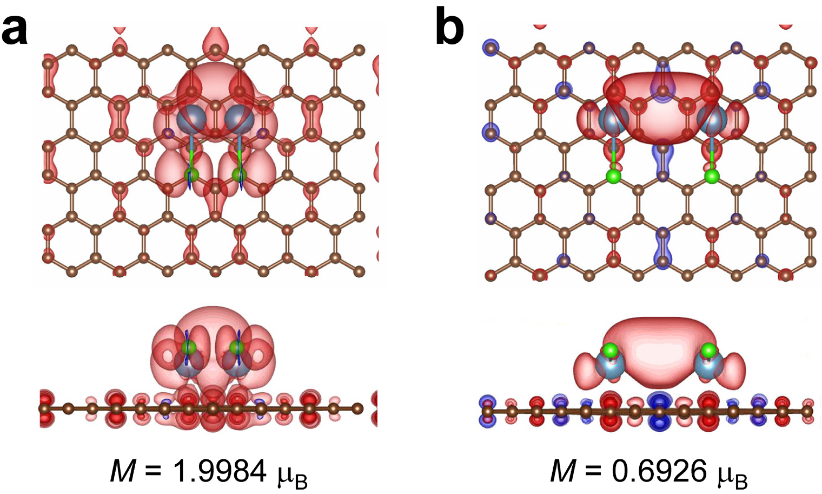


**Fig. S38. Iso-surface spin density plots and total magnetic moments of two CaCl molecules with different distances in one unit cell on graphene substrate.** Top and bottom plots are top views and side views of the cell in **a** and **b**. The red and blue iso-surfaces represent positive and negative spin density of 0.0005 e/bohr^3^.


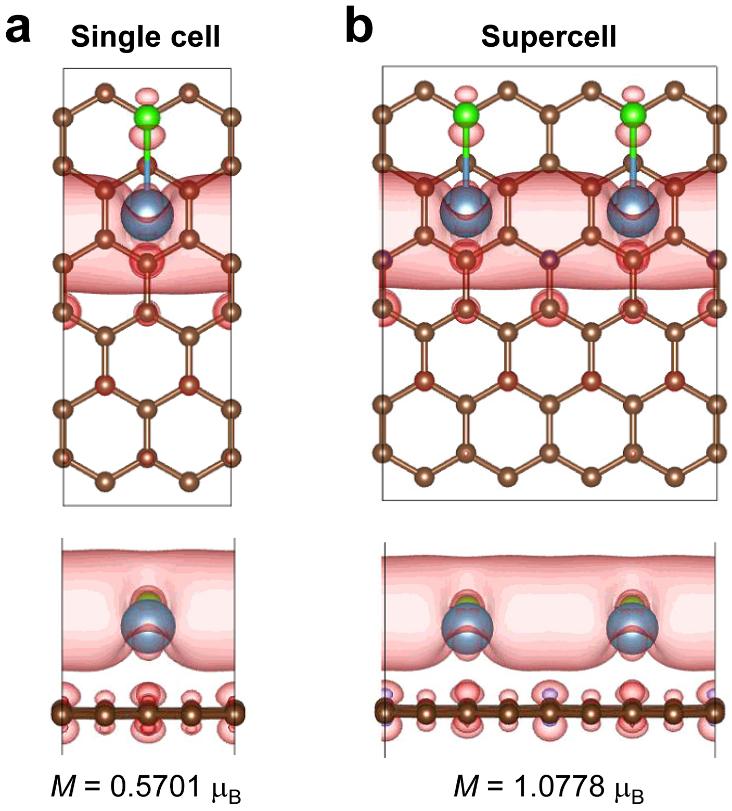


**Fig. S39. Iso-surface spin density plots and total magnetic moments of a single cell containing one CaCl molecule (a) and a supercell containing two CaCl molecules (b) on graphene substrate.** Top and bottom plots are top views and side views of the cell, respectively. The red and blue iso-surfaces represent positive and negative spin density of 0.0005 e/bohr^3^, respectively.


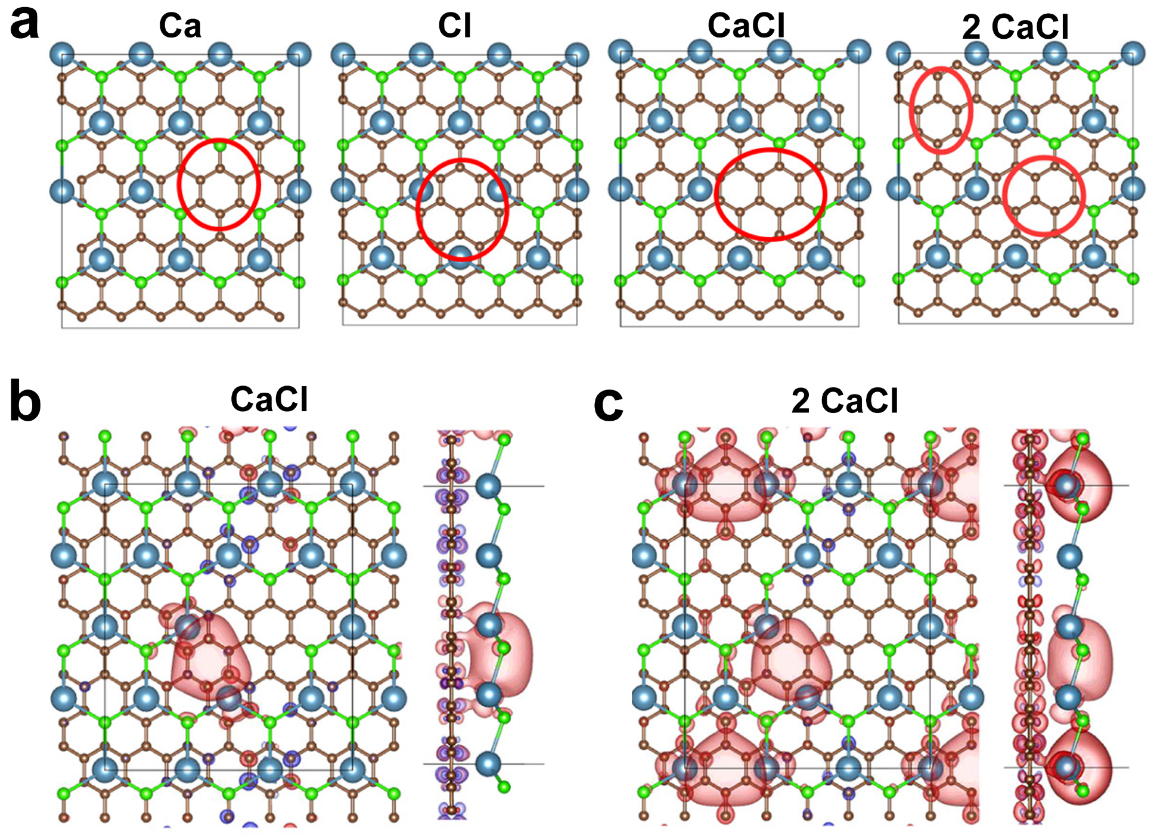


**Fig. S40. Magnetic properties of point defects. a**, Configurations of point defects in the case of one Ca, one Cl, one CaCl, and two CaCl in one unit cell with model I configuration, respectively. **b**, Iso-surface spin density plots of a unit cell containing one CaCl point defect. **c,** Iso-surface spin density plots of a unit cell containing two CaCl point defects. Left and right plots in b and c are top views and side views, respectively. The red and blue iso-surfaces represent positive and negative spin density of 0.0005 e/bohr^3^, respectively.

**Table S8. Magnetic moments of a unit cell containing the point defects as shown in Fig. S40a.**

|  | Ca | Cl | CaCl | 2 CaCl |
| --- | --- | --- | --- | --- |
| Total moment *M* (μ_B_) | 0 | 0 | 0.8972 | 1.9989 |

**PS10: Hydrogen storage capacity calculation and measurement**

To explore the potential application of the system in hydrogen storage, we first calculated a single H_2_ molecule adsorbed on a 2×2 supercell of model I. The optimized atomic configuration is shown in Fig. S41, which shows that H_2_ molecule is adsorbed above the calcium ion at a distance of D = 2.61 Å, with an adsorption energy of 0.23 eV. In order to examine whether the H_2_ molecule can be easily adsorbed on the CaCl, its binding process has been simulated. By decreasing the distance between H_2_ molecule and Ca, a relationship of total energy against the distance can be obtained (Fig. S41b), which shows that the relative energy decreases significantly while the distance is smaller than 6.50 Å and increases dramatically while the distance is smaller than 2.61 Å. Further, it clear shows that there is no energy barrier presented during the binding process, which implies that the CaCl surface can easily attract H_2_ molecules.

High storage capacity is a key factor for a promising hydrogen storage material. By adding H_2_ molecules to this system, a maximal number of 3 H_2_ molecules stably adsorbed on each calcium ion in the super cell had been determined after geometric optimization (Fig. S41c), with a adsorption energy of 0.15 eV per H_2_ molecule. To study the volume storage capacity, a periodic AA stacking model I had been built and investigated. After geometric optimization, the lattice constant along the vertical direction is determined to be 7.20 Å (Fig. S41d), yielding a high H_2_ storage capacity of ~65.8 g/L. This capacity meets the U.S. Department of Energy (DOE) year 2020 volumetric density target (40 g/L) for onboard hydrogen storage for light-duty fuel cell vehicles [63].


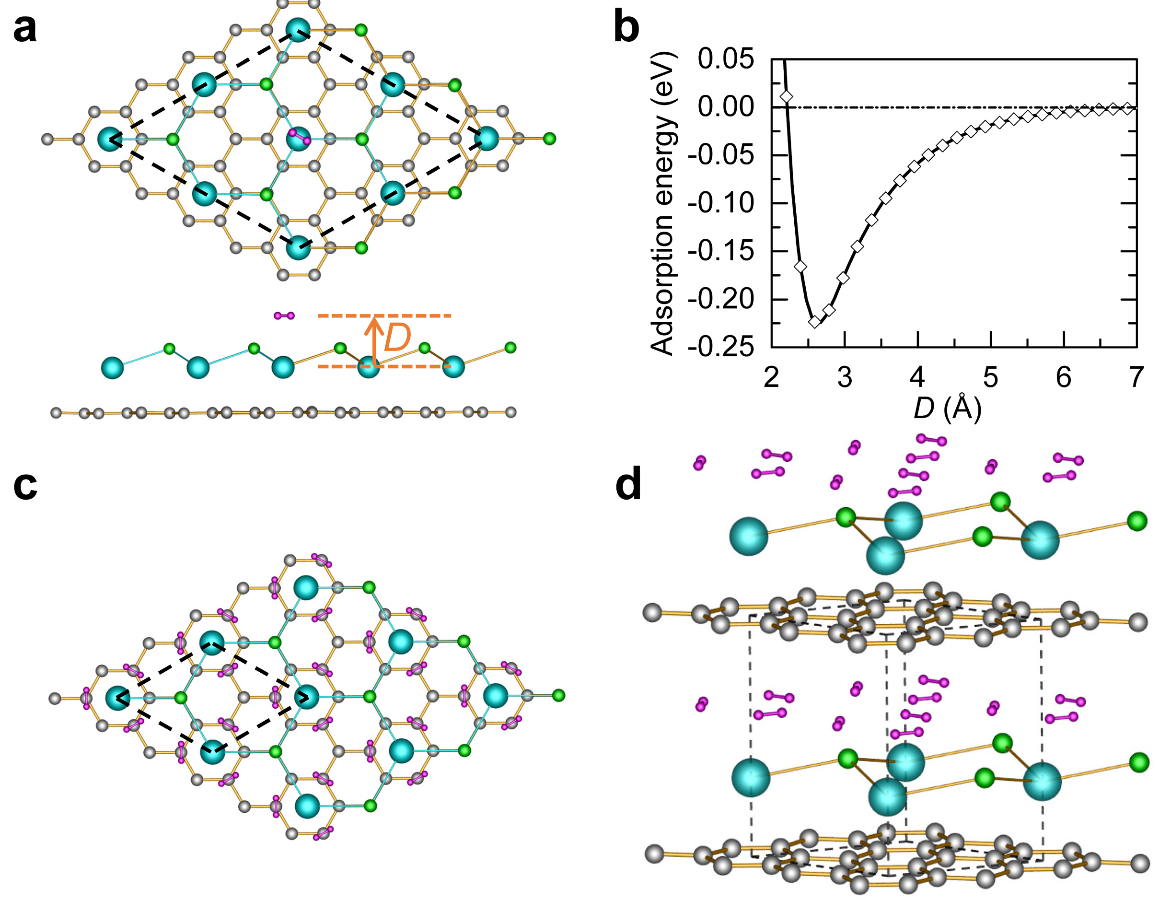


**Fig. S41. Atomic configurations and adsorption energy of hydrogen storage on graphene surface with CaCl crystals.** **a**, The top (up) and side (down) view of a H_2_ molecule absorbed on a Ca in model I. **b**, Relative adsorption energy of the H_2_ molecule against the distance between H_2_ molecule and Ca surface. **c**, The top view of 3 H_2_ molecules adsorbed on each Ca in model I. **d**, The side view of H_2_ storage in a stacked model I after structural optimization.

To study the thermodynamic stability of such hydrogen storage system and estimate the critical temperature for hydrogen storage and release, AIMD calculations were performed on a 2×2×2 supercell of this stacked model I within VASP and the results are plotted in Fig. S42. The system maintains good stability at rang of 0–200 K. While the temperature exceeds 200 K, the root-mean-square deviation (RMSD) of H_2_ molecules rise dramatically, suggesting that such stacked structure are stable up to 200 K while the hydrogen molecules are released above this temperature and CaCl crystals remained for further recycling. Considering such rGO + CaCl nanostructures could be easily prepared, this graphene-based system could be severed as a practically efficient medium for hydrogen storage.


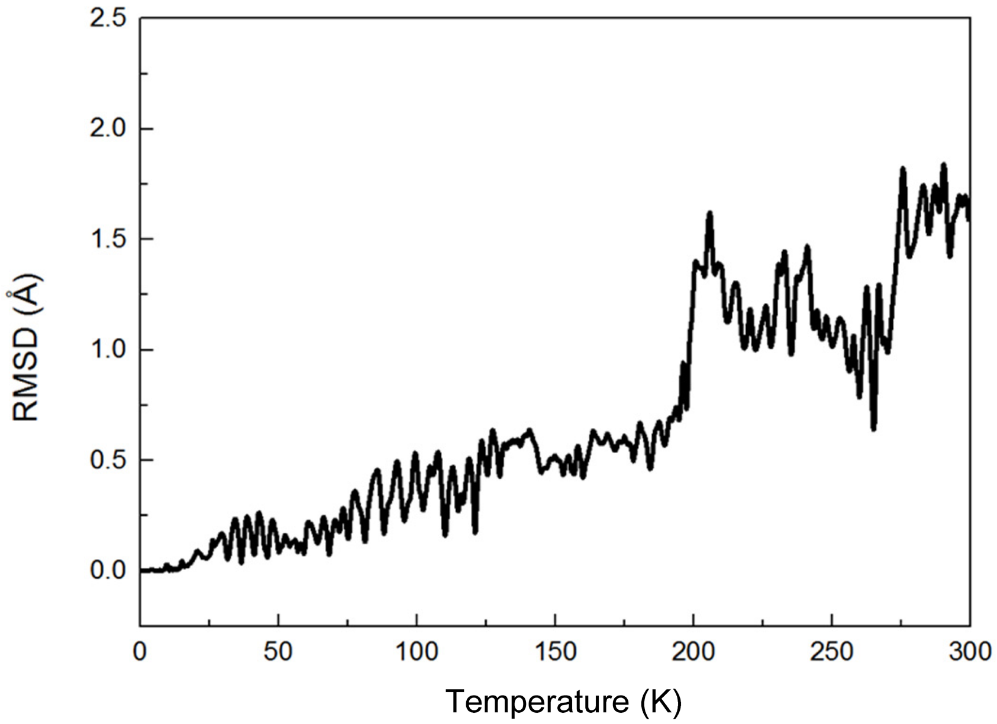


**Fig. S42. Thermodynamic stability analysis of hydrogen stored in the stacked model I.**

To demonstrate the hydrogen storage and release capability of the Ca–Cl crystals in the dried rGO membranes, we have performed new experiments and measured the hydrogen adsorption isotherms at 77 K and with pressure from vacuum to standard atmospheric pressure with a surface area and porosity analyzer (ASAP 2020) (Fig. S43). The amount of hydrogen adsorbed to the dried Ca–Cl@rGO membrane is ~0.4 mmol/g under 1 atmospheric pressure, whereas the rGO membrane only stores ~0.1 mmol/g under the same conditions. These results show that there is an increase of ~300% in the hydrogen storage capability of the dried Ca–Cl@rGO membrane compared with pure rGO, indicating significant enhancement in the hydrogen storage capability induced by existence of Ca–Cl crystals in the rGO membrane, i.e., the hydrogen storage capacity for the CaCl crystals is ~ 3.4 mmol/g assuming all the calcium in the rGO membrane is in the form of CaCl crystals. Notably, the desorption curve of the dried Ca–Cl@rGO membrane is below the adsorption curve, indicating that the Ca–Cl@rGO membrane not only has enhanced hydrogen storage capacity, but it also possesses the property of easy hydrogen release. We note that Ca^2+^ has a relatively high capacity to store hydrogen but the high binding energy of H_2_ [64] causes high energy costs and operational difficulties for hydrogen release. Thus, the +1 valence state in these Ca–Cl crystals provides distinct hydrogen storage and release abilities, which not only enhance adsorption compared with metallic calcium atoms, but also overcomes the strong adsorption of +2 calcium ions.


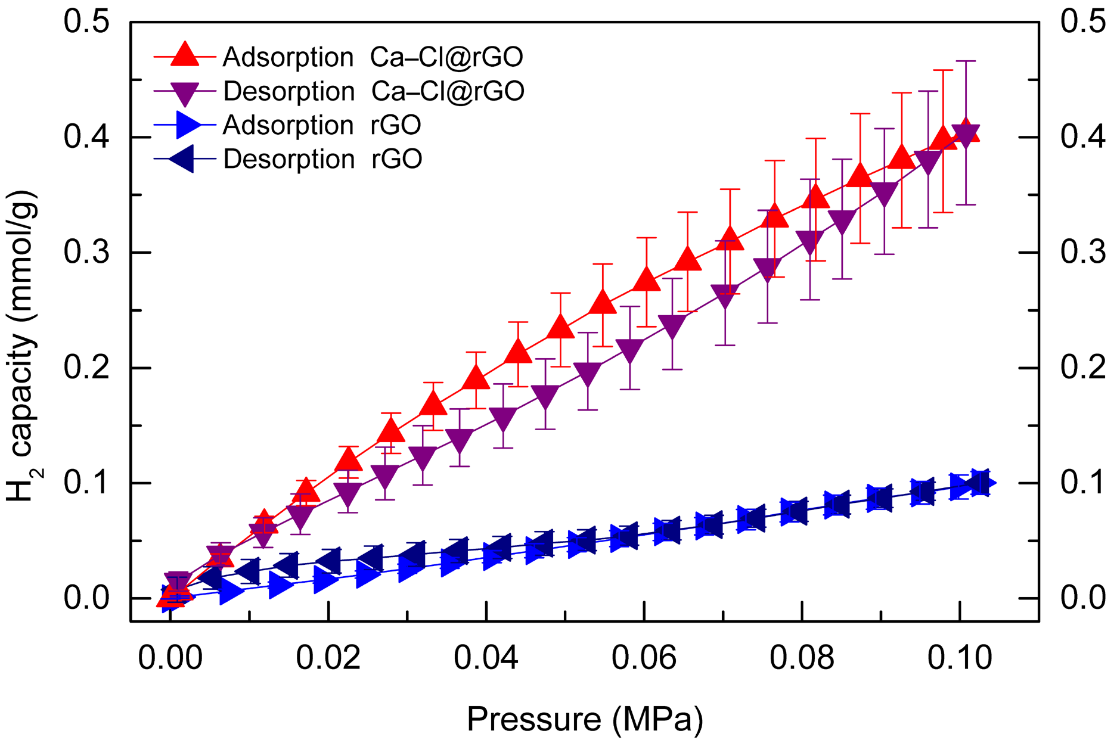


**Fig. S43. H_2_ adsorption and desorption isotherms of the dried Ca–Cl@rGO and rGO membranes at 77 K.** The hydrogen adsorption and desorption curves of the rGO membrane are shown in blue and navy, respectively. The hydrogen adsorption and desorption curves of the dried Ca–Cl@rGO membrane are shown in red and purple, respectively.

**References**

34. Kresse G and Furthmuller J. Efficient iterative schemes for ab initio total-energy calculations using a plane-wave basis set. *Phys Rev B* 1996; **54**: 11169-86.

35. Blochl PE. Projector augmented-wave method. *Phys Rev B* 1994; **50**: 17953-79.

36. Kresse G and Joubert D. From ultrasoft pseudopotentials to the projector augmented-wave method. *Phys Rev B* 1999; **59**: 1758-75.

37. Perdew JP, Burke K and Ernzerhof M. Generalized gradient approximation made simple. *Phys Rev Lett* 1996; **77**: 3865-8.

38. Grimme S, Antony J, and Ehrlich S *et al.* A consistent and accurate ab initio parametrization of density functional dispersion correction (DFT-D) for the 94 elements H-Pu. *J Chem Phys* 2010; **132**: 154104.

39. Monkhorst HJ and Pack JD. Special points for Brillouin-zone integrations. *Phys Rev B* 1976; **13**: 5188-92.

40. Perdew JP and Wang Y. Accurate and Simple Analytic Representation of the Electron-Gas Correlation-Energy. *Phys Rev B* 1992; **45**: 13244-9.

41. Nose S. A molecular dynamics method for simulations in the canonical ensemble. *Mol Phys* 2002; **100**: 191-8.

42. Henkelman G, Arnaldsson A and Jonsson H. A fast and robust algorithm for Bader decomposition of charge density. *Comp Mater Sci* 2006; **36**: 354-60.

43. Sanville E, Kenny SD and Smith R *et al.* Improved grid-based algorithm for Bader charge allocation. *J Comput Chem* 2007; **28**: 899-908.

44. Tang W, Sanville E and Henkelman G. A grid-based Bader analysis algorithm without lattice bias. *J Phys-Condens Mat* 2009; **21**: 084204.

45. Schwarz K, Blaha P and Madsen GKH. Electronic structure calculations of solids using the WIEN2k package for material sciences. *Comput Phys Commun* 2002; **147**: 71-6.

46. Ambrosch-Draxl C and Sofo JO. Linear optical properties of solids within the full-potential linearized augmented planewave method. *Comput Phys Commun* 2006; **175**: 1-14.

47. Oganov AR and Glass CW. Crystal structure prediction using ab initio evolutionary techniques: Principles and applications. *J Chem Phys* 2006; **124**: 244704.

48. Orozco CA, Chun BW and Geng GQ *et al.* Characterization of the Bonds Developed between Calcium Silicate Hydrate and Polycarboxylate-Based Superplasticizers with Silyl Functionalities. *Langmuir* 2017; **33**: 3404-12.

49. Stöhr Joachim. *NEXAFS spectroscopy*. New York: Springer, 1996.

50. Tsao C, Yu PT and Lo CH *et al.* Anhydrous amorphous calcium carbonate (ACC) is structurally different from the transient phase of biogenic ACC. *Chem Commun* 2019; **55**: 6946-9.

51. Kubin M, Kern J and Guo MY *et al.* X-ray-induced sample damage at the Mn L-edge: a case study for soft X-ray spectroscopy of transition metal complexes in solution. *Phys Chem Chem Phys* 2018; **20**: 16817-27.

52. Engerer LK and Hanusa TP. Geometric Effects in Olefinic Cation-π Interactions with Alkali Metals: A Computational Study. *J Org Chem* 2011; **76**: 42-9.

53. Lucas X, Quinonero D and Frontera A *et al.* Counterintuitive Substituent Effect of the Ethynyl Group in Ion-π Interactions. *J. Phys. Chem. A* 2009; **113**: 10367-75.

54. Moffett JW and Zika RG. Reaction-Kinetics of Hydrogen-Peroxide with Copper and Iron in Seawater. *Environ Sci Technol* 1987; **21**: 804-10.

55. Gawande MB, Goswami A and Felpin FX *et al.* Cu and Cu-Based Nanoparticles: Synthesis and Applications in Review Catalysis. *Chem Rev* 2016; **116**: 3722-811.

56. Paulson S, Helser A and Nardelli MB *et al.* Tunable resistance of a carbon nanotube-graphite interface. *Science* 2000; **290**: 1742-4.

57. Zhuang XY, He B and Javvaji B *et al.* Intrinsic bending flexoelectric constants in two-dimensional materials. *Phys Rev B* 2019; **99**: 054105.

58. Hinchet R, Khan U and Falconi C *et al.* Piezoelectric properties in two-dimensional materials: Simulations and experiments. *Mater Today* 2018; **21**: 611-30.

59. Magda GZ, Jin XZ and Hagymasi I *et al.* Room-temperature magnetic order on zigzag edges of narrow graphene nanoribbons. *Nature* 2014; **514**: 608-11.

60. Fernandez-Rossier J and Palacios JJ. Magnetism in graphene nanoislands. *Phys Rev Lett* 2007; **99**: 177204.

61. Yazyev OV. Emergence of magnetism in graphene materials and nanostructures. *Rep Prog Phys* 2010; **73**: 056501.

62. Nair RR, Sepioni M and Tsai IL *et al.* Spin-half paramagnetism in graphene induced by point defects. *Nat Phys* 2012; **8**: 199-202.

63. Zhang B and Wu Y. Recent advances in improving performances of the lightweight complex hydrides Li-Mg-N-H system. *Prog Nat Sci-Mater* 2017; **27**: 21-33.

64. Kim YH, Sun YY and Zhang SB. Ab initio calculations predicting the existence of an oxidized calcium dihydrogen complex to store molecular hydrogen in densities up to 100 g/L. *Phys Rev B* 2009; **79**: 115424.
